# Multivariate Time Series Imputation: An Approach Based on Dictionary Learning

**DOI:** 10.3390/e24081057

**Published:** 2022-07-31

**Authors:** Xiaomeng Zheng, Bogdan Dumitrescu, Jiamou Liu, Ciprian Doru Giurcăneanu

**Affiliations:** 1 Department of Statistics, University of Auckland, Auckland 1142, New Zealand; xzhe229@aucklanduni.ac.nz; 2 Department of Automatic Control and Computers, University Politehnica of Bucharest, 060042 Bucharest, Romania; bogdan.dumitrescu@upb.ro; 3 School of Computer Science, University of Auckland, Auckland 1142, New Zealand; jiamou.liu@auckland.ac.nz

**Keywords:** multivariate time series, missing data, imputation, dictionary learning, information theoretic criteria

## Abstract

The problem addressed by dictionary learning (DL) is the representation of data as a sparse linear combination of columns of a matrix called dictionary. Both the dictionary and the sparse representations are learned from the data. We show how DL can be employed in the imputation of multivariate time series. We use a structured dictionary, which is comprised of one block for each time series and a common block for all the time series. The size of each block and the sparsity level of the representation are selected by using information theoretic criteria. The objective function used in learning is designed to minimize either the sum of the squared errors or the sum of the magnitudes of the errors. We propose dimensionality reduction techniques for the case of high-dimensional time series. For demonstrating how the new algorithms can be used in practical applications, we conduct a large set of experiments on five real-life data sets. The missing data (MD) are simulated according to various scenarios where both the percentage of MD and the length of the sequences of MD are considered. This allows us to identify the situations in which the novel DL-based methods are superior to the existing methods.

## 1. Introduction

### 1.1. Background

It is well-known from the classical literature on time series that a multivariate time series data set is obtained by measuring K>1 variables at time points 1,…,T. The observations are stored in a matrix with *T* rows and *K* columns. For ease of writing, we use the notation Z for this matrix. In our notation, the bold letters are used for vectors and matrices. Due to recent technological advances, both *T* and *K* are very large for the data sets that are collected nowadays. The massive amount of data poses difficulties for both the storage and the processing. Another challenge comes from the fact that some of the entries of the big matrix Z are missing. The data are incomplete for various reasons: malfunction of the sensors, problems with the transmission of the measurements between devices, or the fact that it is practically impossible to collect all the data (for example, this happens often in astronomy).

The conventional approach is to estimate the missing data and then to use the resulting complete data set in statistical inference. The estimation methods span a wide range from the simple ones that perform imputation for each time series individually by considering the mean or the median, or employ the last value carried forward or the next value carried backward, to the more advanced ones that involve the evaluation of the (Gaussian) likelihood, see for example [1].

In here we do not discuss the imputation methods that can be easily found in the time series textbooks, but we briefly present the newer methods that have been compared in [2]. For instance, we consider the method DynaMMO from [3], which is closer to the traditional methods in the sense that it uses a technique akin to the Kalman filter (see again [1]) to estimate the missing values. The other methods that we outline below are based on the decomposition of the matrix Z; hence, it is not surprising that they have a certain relationship with the singular value decomposition (SVD). In fact, SVDImp from [4] explicitly uses the SVD factorization $U\Sigma V^{⊤}$ of the matrix Z, after replacing the missing values with the mean computed for the corresponding row. Note that the symbol ${\left(\cdot \right)}^{⊤}$ denotes transposition. The most significant κ rows of $V^{⊤}$ are selected, where the value of κ is chosen empirically. Then the linear regression is used to express each row of Z as a linear combination of the most significant κ rows of $V^{⊤}$. In this way, new estimates are obtained for the missing data, and the procedure above is applied again to the matrix Z that contains these imputed values. The iterations are continued until the change of the magnitudes of the imputed values between two consecutive iterations is smaller than a threshold selected by the user.

In [5], the imputation problem is formulated as a matrix completion problem. The obtained estimate $\hat{Z}$ has the same entries as Z at the locations for which the measurements are available. The matrix $\hat{Z}$ is found by solving a penalized least-squares problem whose expression contains a coefficient λ that balances the two terms involved: (i) half of the sum of the squares of the approximation errors, or equivalently $\frac{1}{2}\left|\right|\hat{Z}-Z{\left|\right|}_{F}^{2}$ (the notation $\left|\right|\cdot {\left|\right|}_{F}$ stands for the Frobenius norm), and (ii) the sum of the singular values of $\hat{Z}$, or equivalently the nuclear norm of $\hat{Z}$. The solution is given by a soft-thresholded SVD of Z, where the soft threshold is λ. This suggests the name Soft-Impute of the method for which we use the acronym SoftImp. In SoftImp, the solutions are obtained in a computationally efficient manner for all the values of λ on a predefined grid. Another algorithm that solves the same penalized least-squares problem by computing the soft-thresholded SVD at each iteration is the Singular Value Thresholding (SVT) from [6]. A particular attribute of SVT is that it automatically finds the optimal rank of the estimated matrix.

An approximation of SVD, which is called centroid decomposition (CD), is employed in [7,8] for representing the matrix Z as $Z=LR^{⊤}$, where $L\in R^{T\times K}$ and $R\in R^{K\times K}$. Either interpolation or extrapolation is applied for estimating the missing entries of Z and then a vector $s\in {\left\{-1,1\right\}}^{T}$ is found such that the Euclidean norm $\left|\right|Z^{⊤}s{\left|\right|}_{2}$ is maximized. The vector $c=Z^{⊤}s$ is obtained and is further used to obtain the first column of R, $R_{:1}=c/{\left||c|\right|}_{2}$, and the first column of L, $L_{:1}=ZR_{:1}$. For a better understanding of why the method is named CD, we mention that c is the first centroid vector. The “new” matrix Z is taken to be $Z-L_{:1}R_{:1}^{⊤}$, and the algorithm continues until all *K* columns of L and R are obtained. Only the first κ columns are used to obtain the approximation of Z given by $\sum_{i=1}^{\kappa }L_{:i}R_{:i}^{⊤}$, and this approximation yields the estimates for the missing values. It is interesting that the selection of κ is performed by an entropy-based criterion. When CD is employed in time series recovery we call it CDRec (as in [2]).

Another imputation method is dubbed Grassmannian Rank-One Update Subspace Estimation (GROUSE), see for example [9,10]. The name comes from the set of all subspaces of $R^{T}$ of dimension κ that is called Grassmannian. It is evident that an element of the Grassmanian can be represented by any matrix $U\in R^{T\times \kappa }$ with the property that $U^{⊤}U=I$, where I denotes the identity matrix of appropriate dimension. GROUSE finds the matrix U that minimizes the objective function $\sum_{j=1}^{K}\left|\right|\Delta_{j}\left(Z_{:j}-UU^{⊤}Z_{:j}\right){\left|\right|}_{2}^{2}$, where $\Delta_{j}\in R^{T\times T}$ is a diagonal matrix which has on the main diagonal ones on the locations corresponding to the data that are available for the *j*th column of Z and zeros otherwise. The presence of $\Delta_{j}$ in the formula above shows that the algorithm can work directly with the columns of Z that have missing data. In fact, GROUSE optimizes the cost function by considering one column of Z at a time, and at each such step, the matrix U is updated by adding a rank-one matrix to the matrix U obtained at the previous step. Once the “final” U is found at the last step, the incomplete columns are projected onto the low-rank subspace that was identified to complete the matrix.

In robust principal component analysis (RPCA), the data matrix (which is supposed to be complete) is represented as a low-rank matrix plus a sparse matrix [11]. Because the recovery of the low-rank matrix is computationally intensive, an efficient algorithm called Robust Orthogonal Subspace Learning (ROSL) was proposed in [12]. The algorithm was altered in [2] to be applied to an incomplete data matrix for estimating the missing values. We use the acronym ROSL for the version of the algorithm from [2].

The imputation method from [13] relies on the nonnegative matrix factorization (NMF) technique and, to be suitable for electricity consumption, uses temporal aggregates. The optimization problem solved by the algorithm proposed in [13] takes into consideration the correlation between time series. As in [2], we call this method temporal NMF (TeNMF). The matrix factorization is also used in [14], but in contrast to TeNMF, the entries of the two factor matrices are not constrained to be nonnegative. An important feature of the method is that the regularization term of the objective function takes explicitly into consideration the temporal structures, and this is why the method is termed Temporal Regularized Matrix Factorization (TRMF).

Another matrix factorization that can be instrumental in time series imputation is the one generated by dictionary learning (DL).

### 1.2. Organization of the Paper and the Main Contributions

In this article, we extend the DL-based solution for time series imputation which we proposed in our earlier work [15]. Our previous results from [15] consist of two imputation methods: DLU (for univariate time series) and DLM (for multivariate time series). However, because of the computational complexity, the original version of DLM can be utilized only when the number of the time series involved is very small. In contrast, the variants of DLM that we introduce in this study can be applied to data sets that contain tens of time series.

DLM is presented in Section 2.2 after briefly discussing the DL optimization problem in Section 2.1. An important characteristic of DLM is that it solves the optimization problem by minimizing the Frobenius norm of the errors. In many practical situations, the imputation should be performed to minimize the $\ell_{1}$-norm of the errors and not the sum of the squared errors. In Section 3, we demonstrate how the optimization problem can be solved when the Frobenius norm is replaced with the sum of the magnitudes of errors. The method that involves the $\ell_{1}$-norm is dubbed ${\mathrm{DLM}}_{1}$. Another characteristic of DLM (which is also inherited by ${\mathrm{DLM}}_{1}$) is the use of a structured dictionary. In Section 4, we present the expressions of the IT criteria that are employed to select the size of the structured dictionary, the size for each of its blocks as well as the sparsity level of the representation. Section 5 is focused on the techniques that we propose for dimensionality reduction. It allows us to apply DLM and ${\mathrm{DLM}}_{1}$ to data sets that comprise tens of time series with thousands of measurements. For demonstrating how the new algorithms can be used in practice, we conduct a large set of experiments with real-life data. The experimental settings are presented in Section 6, and the empirical results are discussed in Section 7. Section 8 concludes the paper.

Hence, the main contributions of this work are the following:

- A flexible approach that allows the user to choose the norm of the errors (Frobenius norm or $\ell_{1}$-norm) minimized in the optimization problem.
- An automatic method for selecting the sparsity as well as the size for each block of the dictionary.
- The exemplification of two techniques for dimensionality reduction that enable DLM to impute values on multivariate time series for which *K* is large.
- An extensive empirical study which compares DLM with nine other imputation methods on data sets with various characteristics. On many of these data sets, DLM has the best performance among the considered methods when the missing data are simulated by sampling without replacement.

## 2. Dictionary Learning for Data Sets with Missing Values

### 2.1. Preliminaries

The DL problem is formulated as follows. Given *N* signals of length *m* that are grouped into the matrix $Y\in R^{m\times N}$, we approximate Y by the product DX, where $D\in R^{m\times n}$ is the *dictionary*, and its columns are usually named *atoms*. The Euclidean norm of each atom equals one. The matrix $X\in R^{n\times N}$ is sparse in the sense that each of its columns contains at most *s* non-zero entries, the parameter *s* being named sparsity level. We emphasize that both D and X are learned from the signals by solving the following optimization problem [16]:

(1) $$\begin{array}{ccc} {\mathrm{minimize}}_{D,X} & & {∥Y-\mathrm{DX}∥}_{F}\\ \mathrm{subject}\;\mathrm{to} & & \left|\right|X_{:\ell }{\left|\right|}_{0}\leq s,\;\;\;\ell \in \left\{1,\ldots ,N\right\}\;\\ & & \left|\right|D_{:j}{\left|\right|}_{2}=1,\;\;\;j\in \left\{1,\ldots ,n\right\} \end{array}$$

the *ℓ*-th column of X is denoted $X_{:\ell }$, and the *j*-th column of D is denoted $D_{:j}$. The symbol ${∥\cdot ∥}_{0}$ represents the number of the non-zero entries for the vector in the argument.

The algorithm that solves the optimization problem in (1) is initialized with a dictionary D, which is generally randomly generated. The user selects the number of iterations, and the following steps are executed at each iteration:

(i) The current dictionary D is used to find the matrix X, which provides a representation for the signals in Y. This goal is achieved by employing the Orthogonal Matching Pursuit (OMP).
(ii) The dictionary D is updated by using the current sparse representation X. This is performed by using the Approximate K-Singular Value Decomposition (AK-SVD) algorithm.

The two steps of the main algorithm are presented in [17].

There are other ways of posing the DL problem. For example, one may add a sparsity enhancing term to the objective and thus impose sparsity globally, not on each representation; thus, one can obtain a matrix X that has around sN nonzeros without the explicit constraint that each of its columns has *s* nonzeros. A representative of this approach is [18]. Convolutional DL [19] does not split the time series into signals of size *m*, but works with a single long signal that is approximated as a linear combination of atoms of length *m* that may be placed at any position; the same atom can be used repeatedly. These approaches may provide more flexibility, but they require more fine tuning of the parameters and adaptation of dictionary size criteria. We prefer AK-SVD because it is one of the fastest DL-algorithms that have been proposed in the previous literature. Another important feature of the algorithm is its conceptual simplicity, which allows it to be easily modified for solving particular formulations of DL that appear in the context of imputation.

### 2.2. Optimization Problem for Incomplete Data: Formulation, Solution and Applications

When some of the entries of the matrix Y are missing, the optimization problem in (1) becomes [20]:

(2) $$\begin{array}{ccc} {\mathrm{minimize}}_{D,X} & & {∥M⊙\left(Y-\mathrm{DX}\right)∥}_{F}\\ \mathrm{subject}\;\mathrm{to} & & \left|\right|X_{:\ell }{\left|\right|}_{0}\leq s,\;\;\;\ell \in \left\{1,\ldots ,N\right\}\;\\ & & \left|\right|D_{:j}{\left|\right|}_{2}=1,\;\;\;j\in \left\{1,\ldots ,n\right\} \end{array}$$

where M is a mask matrix with the same size as Y. Its entries are equal to zero for the positions in Y that correspond to the missing data. All other entries of M are equal to one. The operator ⊙ is the element-wise product. The role of M is to guarantee that only the available data are used in learning. Note that the missing data are replaced with zeros in Y. For the optimization problem in (2), a specialized version of the AK-SVD algorithm is applied [16] [Section 5.9]; the representation matrix X is found using OMP by ignoring the missing samples and working only with the present ones; the atom update formulas are simple adaptations of AK-SVD rules to the incomplete data case.

An important application of (2) consists of filling the gaps of an incomplete image and is called image inpainting. In the case of this application, the matrix Y is generated as follows (see, for example, [16] [Section 2.3.1]). A patch of pixels of size $\sqrt{m}\times \sqrt{m}$ is selected from a random location in the image. Then its columns are stacked to generate the signal y, which is a column of Y. The procedure continues until all *N* signals are produced. Obviously, it is not allowed to select the same patch twice, but it is highly recommended to select patches that overlap. In what concerns the sizes of the patches, the value $\sqrt{m}=8$ is often used. Values such as $\sqrt{m}=12$ and $\sqrt{m}=16$ have also been used, but they are not commonly employed because of the increased computational burden. Once the dictionary is learned, the product DX yields an estimate $\hat{Y}$ of Y. Any missing pixel is obtained by averaging its values from all entries of $\hat{Y}$ where it appears. More details about image inpainting can be found in [21,22,23,24]. The use of the inpainting was extended from images to audio signals in [20,25].

Our main goal is to show how DL can be employed for estimating the values of the missing data in multivariate time series. As we have already pointed out, the use of DL in the imputation of time series was discussed in [15]. The approach adopted in the multivariate case should take into consideration the dynamic interrelationships between K>1 variables whose measurements collected at time points 1,…,T are stored in matrix Z. Suppose that some of the entries of Z are missing. As the positions of the missing data are not necessarily the same for all the time series, we use the symbol $\Psi_{k}$ to denote the indexes of the measurements that are available for the *k*-th time series, where 1≤k≤K. Obviously, the set of indexes of missing data for the *k*-th time series is ${\bar{\Psi }}_{k}=\left\{1,\ldots ,T\right\}\setminus \Psi_{k}$.

Let z be one of the columns of the data matrix Z in which the missing data indexed by $\bar{\Psi }$ are replaced with zeros. We define a matrix Y as follows:

$$\begin{array}{c} Y={\left[z_{1:m}\;z_{1+h:m+h}\cdots z_{1+qh:m+qh}\right]}_{.} \end{array}$$

For an arbitrary vector v, $v_{a:b}$ denotes the entries of the vector whose indexes belong to the set {a,a+1,…,b}, where a<b. The number of rows of the matrix Y is *m*, and its choice depends on the sampling period. The parameter *h* is called signal shift and controls the overlapping between the columns of Y, and the value of *q* is given by ⌊(T−m)/h⌋. It follows that the number of columns of the matrix Y is N=q+1. Herein we take h=1, which leads to q=T−m and N=T−m+1.

If $t\in \bar{\Psi }$, then $z_{t}$ is a missing value, and this will be represented as a zero-entry in Y. As there is an overlap between the columns of Y, the missing value $z_{t}$ leads to several zero-entries in Y. We collect all the values of these entries from $\hat{Y}=DX$ and compute an estimate for $z_{t}$ by averaging them.

For example, suppose $4\in \bar{\Psi }$, which means that $z_{4}$ is missing. Then the matrix Y is given by:

$$Y={\left[\begin{array}{cccccc} z_{1} & z_{2} & z_{3} & 0 & \ldots & z_{T-m+1}\\ z_{2} & z_{3} & 0 & z_{5} & \ldots & z_{T-m+2}\\ z_{3} & 0 & z_{5} & z_{6} & \ldots & z_{T-m+3}\\ 0 & z_{5} & z_{6} & z_{7} & \ldots & z_{T-m+4}\\ z_{5} & z_{6} & z_{7} & z_{8} & \ldots & z_{T-m+5}\\ \vdots & \vdots & \vdots & \vdots & \ddots & \vdots \\ z_{m} & z_{m+1} & z_{m+2} & z_{m+3} & \ldots & z_{T} \end{array}\right]}_{.}$$

In addition, the entries of the matrix $\hat{Y}$ are:

$$\hat{Y}={\left[\begin{array}{cccccc} {\hat{y}}_{1,1} & {\hat{y}}_{1,2} & {\hat{y}}_{1,3} & {\hat{y}}_{1,4} & \ldots & {\hat{y}}_{1,N}\\ {\hat{y}}_{2,1} & {\hat{y}}_{2,2} & {\hat{y}}_{2,3} & {\hat{y}}_{2,4} & \ldots & {\hat{y}}_{2,N}\\ {\hat{y}}_{3,1} & {\hat{y}}_{3,2} & {\hat{y}}_{3,3} & {\hat{y}}_{3,4} & \ldots & {\hat{y}}_{3,N}\\ {\hat{y}}_{4,1} & {\hat{y}}_{4,2} & {\hat{y}}_{4,3} & {\hat{y}}_{4,4} & \ldots & {\hat{y}}_{4,N}\\ \vdots & \vdots & \vdots & \vdots & \ddots & \vdots \\ {\hat{y}}_{m,1} & {\hat{y}}_{m,2} & {\hat{y}}_{m,3} & {\hat{y}}_{m,4} & \ldots & {\hat{y}}_{m,N} \end{array}\right]}_{,}$$

where N=T−m+1. It results that the estimate of the missing value $z_{4}$ is computed by averaging the red-colored entries of $\hat{Y}$, whose positions are the same as the positions of $z_{4}$ in Y. Thus, we obtain ${\hat{z}}_{4}$ as:

$$\begin{array}{c} {\hat{z}}_{4}=\frac{1}{4}{\left({\hat{y}}_{4,1}+{\hat{y}}_{3,2}+{\hat{y}}_{2,3}+{\hat{y}}_{1,4}\right)}_{.} \end{array}$$

This imputation method was introduced in [15]. As it can be easily seen from the description above, the method is suitable for univariate time series, and for this reason it is named DLU (see again Section 1.2). In the multivariate case, the data matrix is

(3) $$\begin{array}{c} Y={\left[Y_{1}\;\ldots \;Y_{K}\right]}_{,} \end{array}$$

where $Y_{i}\in R^{m\times (T-m+1)}$ is made of data measured for the *i*-th time series for i∈{1,…,K}. In [15], it was pointed out that in the DLM algorithm designed for the multivariate case, a structured dictionary should be used:

(4) $$\begin{array}{c} D={\left[D_{1}\;\ldots \;D_{K}\;D_{K+1}\right]}_{,} \end{array}$$

where $D_{1},\ldots ,D_{K}\in R^{m\times n_{d}}$ and $D_{K+1}\in R^{m\times n_{K+1}}$. The dictionary $D_{i}$ is dedicated to the representation of the *i*-th time series, while $D_{K+1}$ is common for all time series. It follows that the number of atoms used in the representation of each time series is $n_{u}=n_{d}+n_{K+1}$. The values of $n_{u}$ and $n_{K+1}$ as well as the sparsity level *s* are selected by using information theoretic (IT) criteria [26]. The main advantage is that the procedure for choosing the triple $\left(n_{u},n_{K+1},s\right)$ does not rely on prior knowledge. We take the sizes of dictionaries $D_{1},\ldots ,D_{K}$ to be equal to simplify the decision process, but also to use similar representation power for all times series (or groups of time series, as we will see later).

We mention that there are many DL algorithms that choose the size of the dictionary. Most of them are based on heuristics, such as, for example, growing a small dictionary [27] or removing atoms from a large one [28], with the general purpose of achieving parsimony. Other approaches are more principled, using Bayesian learning [29] or an Indian Buffet Process [30]. We have used IT criteria in [26], where we also presented a more detailed view of the topic, including bibliographic references. IT criteria offer a sound evaluation of the trade-off between dictionary size and representation error.

Next we show how the new algorithm ${\mathrm{DLM}}_{1}$ can be devised.

## 3. **${\mathrm{DLM}}_{1}$**: DL-Algorithm for Incomplete Data (with **$\ell_{1}$**-Norm)

We solve the $\ell_{1}$-norm version of (2):

(5) $$\begin{array}{ccc} {\mathrm{minimize}}_{D,X} & & {∥M⊙\left(Y-\mathrm{DX}\right)∥}_{1,1}\\ \mathrm{subject}\;\mathrm{to} & & \left|\right|X_{:\ell }{\left|\right|}_{0}\leq s,\;\;\;\ell \in \left\{1,\ldots ,N\right\}\;\\ & & \left|\right|D_{:j}{\left|\right|}_{2}=1,\;\;\;j\in \left\{1,\ldots ,n\right\} \end{array}$$

where for a matrix $G\in R^{m\times N}$ we denote ${∥G∥}_{1,1}=\sum_{i=1}^{m}\sum_{\ell =1}^{N}\left|g_{i\ell }\right|$, the $\ell_{1}$-equivalent of the Frobenius norm. So, the aim of (5) is to optimize the sparse $\ell_{1}$-norm representation of the signals, whereas (2) targets the $\ell_{2}$-norm.

We modify the algorithm proposed in [31] such that it is suitable for the missing data case. The algorithm is an adaptation of the AK-SVD [17] idea and consists of iterations containing the usual two steps, sparse representation and dictionary update, as described in Section 2.1. We will next discuss these steps in detail.

### 3.1. $\ell_{1}$-Norm OMP with Missing Data

We present a $\ell_{1}$-norm version of the greedy approach whose most prominent representative is OMP [32]. It is enough to consider a single signal $y\in R^{m}$, for which we have to minimize ${∥m⊙\left(y-\mathrm{Dx}\right)∥}_{1}$, where $D\in R^{m\times n}$ is the given dictionary, $x\in R^{n}$ must have at most *s* nonzero elements, and $m\in R^{m}$ is the mask whose entries are zeros and ones.

We denote $\bar{y}\in R^{\mu }$ (μ≤m) the vector that results from y by keeping only the elements that correspond to nonzero values in m. Similarly, $\bar{D}\in R^{\mu \times n}$ is the matrix obtained from D by keeping only the rows corresponding to nonzero values in m. We are thus left with the problem $∥\bar{y}-\bar{D}{x∥}_{1}$, which is a usual $\ell_{1}$-norm sparse representation problem for which the algorithm was described in [31].

For the sake of completeness, we revisit the main operations here. The algorithm has *s* steps. Denoting $\tilde{x}$ the representation at the beginning of the current step and $r=\bar{y}-\bar{D}\tilde{x}$ the current residual, the next selected atom ${\bar{d}}_{j}$ is that for which

(6) $$\min_{j\in \{1,\ldots ,n\}}\min_{\xi }{∥r-\xi {\bar{d}}_{j}∥}_{1}$$

is attained. Thus, we follow the idea of finding the atom with the best projection on the residual.

The problem $\min_{\xi }{∥r-\xi d∥}_{1}$ (we lighten the notation for the sake of simplicity) can be easily solved. It is not only convex, but its solution can be found by inspection [33]. Denote $c_{i}=r_{i}/d_{i}$, for i∈{1,…,μ}. Denote $\tilde{c}$ the vector containing the elements of c sorted increasingly and π(·) the permutation for which ${\tilde{c}}_{i}=c_{\pi (i)}$. Denote ${\tilde{d}}_{i}=d_{\pi (i)}$. The desired minimum is $\xi =c_{\pi (k)}$, where the index *k* is the largest for which

$$\begin{array}{c} \sum_{i=1}^{k-1}|{\tilde{d}}_{j}|\leq \sum_{i=k}^{\mu }\left|{\tilde{d}}_{j}\right|. \end{array}$$

So, finding the solution essentially requires only a sort operation. Moreover, in solving (6), some atoms can be ignored if their scalar product (usual orthogonal projection) with the residual is small.

Once the current atom has been found, it is added to the support, and the optimal $\ell_{1}$-norm representation with that support is computed. This is a convex problem and can be solved by several nonlinear optimization algorithms (we have used a few coordinate descent iterations). Moreover, a good initialization is available in the representation at the previous step. (In OMP, these operations correspond to finding a least-squares solution.)

### 3.2. Dictionary Update with Missing Data in the $\ell_{1}$-Norm

The update stage of AK-SVD optimizes the atoms one by one, also updating the coefficients of the corresponding representations. Denote $d_{j}$ the current atom and $I_{j}$ the set of signals where this atom contributes to the representation. Denote E=Y−DX the current residual and

$$\begin{array}{c} R={\left[Y-\sum_{i\neq j}d_{i}x_{i}^{⊤}\right]}_{I_{j}}={\left[E+d_{j}x_{j}^{⊤}\right]}_{I_{j}}{}_{,} \end{array}$$

the error without the contribution of $d_{j}$, keeping only the columns where $d_{j}$ appears in the representation.

With lighter notation, namely d for the current atom, x for the vector of its nonzero representation coefficients and M for the mask (even though the signals where d is not used are removed), the atom update problem becomes

(7) $$\min_{d}{∥M⊙(R-{\mathrm{dx}}^{⊤})∥}_{1,1}.$$

Denoting $M\subset N^{2}$ the indexes of available data, the problem can be written as

(8) $$\min_{d}\sum_{(i,j)\in M}\left|r_{ij}-d_{i}x_{j}\right|.$$

The minimization can be performed on each $d_{i}$ separately and has the form $\min_{d_{i}}{∥\hat{r}-d_{i}\hat{x}∥}_{1}$, where $\hat{r}$ and $\hat{x}$ are vectors that can be easily built. We thus end up with a problem similar to that described after (6).

Keeping the updated atom fixed, we can now optimize the associated representation coefficients by solving

$$\begin{array}{c} \min_{x}{∥M⊙(R-{\mathrm{>dx}}^{⊤})∥}_{1,1}, \end{array}$$

which can be written

$$\begin{array}{c} \min_{x}\sum_{(i,j)\in M}\left|r_{ij}-d_{i}x_{j}\right| \end{array}$$

like (7) was written as (8). The problem is separable on each $x_{j}$ and can be solved as above.

We note that we use the same basic algorithm in the $\ell_{1}$-norm OMP, atom and representation update. The approach can be extended to $\ell_{p}$-norms, with p≠1, transforming the $\ell_{p}$-norm AK-SVD from [31] to the missing data case, similarly to the transformations described in this section.

## 4. Information Theoretic Criteria

We have already mentioned in Section 2.2 that we employ IT criteria for selecting the triple $\left(n_{u},n_{K+1},s\right)$. More precisely, the criteria that we use are derived from the well-known Bayesian information criterion (BIC) [34]. For evaluating the complexity of the model, we need to calculate the number of parameters (NoP). We have that NoP=sN+(m−1)n. The first term is given by the number of the non-zero entries for the representation matrix $\hat{X}$, which is estimated from the available data by solving the optimization problem (2). The second term is equal to the number of the entries of the estimated dictionary $\hat{D}$; for each column of $\hat{D}$, we count m−1 entries (and not *m* entries) because each column is constrained to have the Euclidean norm equal to one. Furthermore, we define the matrix of residuals $\hat{U}=M⊙\left(Y-\hat{D}\hat{X}\right)$. Note that the residuals located at the positions corresponding to the missing data are forced to be zero. With the understanding that η is the number of the entries of M that are equal to one, the expression of the first IT criterion that we employ is:

(9) $$\begin{array}{c} \mathrm{BIC}\left(Y;n_{u},n_{K+1},s\right)=\frac{\eta }{2}\ln\frac{\left|\right|\hat{U}{\left|\right|}_{F}^{2}}{\eta }+\frac{\mathrm{NoP}}{2}\ln\eta , \end{array}$$

where ln(·) denotes the natural logarithm. This criterion was proposed in [15], where its “extended” variant was also used (see [26,35]):

(10) EBIC(Y;nu,nK+1,s)=BIC(Y;nu,nK+1,s)+Nlnns.

However, because of the way in which they have been derived, the formulas in (9) and (10) can only be used when $\hat{D}$ and $\hat{X}$ are outputs of the DLM algorithm. When the estimation is performed by applying the ${\mathrm{DLM}}_{1}$ algorithm from Section 3, we employ the following formula for BIC:

(11) $$\begin{array}{c} \mathrm{BIC}\left(Y;n_{u},n_{K+1},s\right)=\frac{\eta }{2}\ln\frac{2||\hat{U}{\left|\right|}_{1,1}^{2}}{\eta^{2}}+\frac{\mathrm{NoP}}{2}\ln\left(2\eta \right). \end{array}$$

The expression above is based on the criterion obtained in [31] for signals in additive Laplacian noise and is altered to be suitable for the missing data case. Its “extended” variant is easily constructed by adding the term Nlnns to the expression above (see again [31]).

For clarifying the notation, we mention that when we write DLM+BIC, it means that the criterion in (9) is applied for model selection. At the same time, DLM_1_+BIC involves the criterion from (11). A similar convention is used for the “extended” criteria.

Whenever DLM is applied to multivariate time series with missing values, 10 random initializations of the dictionary are considered. For each initialization, 50 iterations of the two-step algorithm that involves OMP and AK-SVD for incomplete data are executed for each possible combination of $n_{u}$, $n_{K+1}$ and *s*. Then the triple ($n_{u}$, $n_{K+1}$, *s*) which minimizes BIC/EBIC is selected. The procedure is the same when ${\mathrm{DLM}}_{1}$ is used instead of DLM.

## 5. Dimensionality Reduction

### 5.1. Dimensionality Reduction via Clustering

When *K* is large, we group the time series to reduce the dimensionality. For exemplification, we refer to the particular case in which *K* is an even number, and the time series are clustered into two groups such that each group contains K/2 columns of Z. The set of the indexes of the time series that belong to the first group is $\Phi =\{\phi_{1},\ldots ,\phi_{K/2}\}$, whereas the set of the indexes corresponding to the second group is $\bar{\Phi }=\left\{{\bar{\phi }}_{1},\ldots ,{\bar{\phi }}_{K/2}\right\}$. It is evident that $\Phi \cup \bar{\Phi }=\left\{1,\ldots ,K\right\}$ and $\Phi \cap \bar{\Phi }=\emptyset$. Furthermore, we re-arrange the columns of the matrix $Z\in R^{T\times K}$ into the matrix $Z^{\left(c\right)}$ as follows:

$$Z^{\left(c\right)}={\left[\begin{array}{cc} z_{\phi_{1}} & z_{{\bar{\phi }}_{1}}\\ \vdots & \vdots \\ z_{\phi_{K/2}} & z_{{\bar{\phi }}_{K/2}} \end{array}\right]}_{.}$$

The newly obtained matrix $Z^{\left(c\right)}$ is regarded as a multivariate time series that contains $K^{\left(c\right)}=2$ time series observed at time points $1,\ldots ,T^{\left(c\right)}$, where $T^{\left(c\right)}=\left(K/2\right)\times T$. Hence, the data matrix Y used by the DL algorithm (see (3)) has the expression $Y=[Y_{1}\;Y_{2}]$ where, for i∈{1,2}, $Y_{i}\in R^{m\times (T^{\left(c\right)}-m+1)}$ is constructed from the entries of the *i*-th column of $Z^{\left(c\right)}$ as in Section 2.2. According to the convention from (4), the structure of the dictionary D is given by $D=[D_{1}\;D_{2}\;D_{3}]$. It follows that the block $D_{1}$ is for the time series in group Φ, the block $D_{2}$ is for the time series in group $\bar{\Phi }$ and the block $D_{3}$ is common for all the time series. The estimation of the missing values is performed as it was described in the previous sections.

### 5.2. Time Series Grouping

When deciding what time series to assign to the group Φ, we should take into consideration that all these time series are represented by using atoms from the same blocks: $D_{1}$ and $D_{3}$. Hence, it is desirable (i) to minimize the overlap of the sequences of missing data for the columns of Z that belong to Φ, and (ii) to maximize the linear dependence between any two time series in Φ. Because it is non-trivial to combine the two requirements, we first focused on the condition (i). After some preliminary experiments, we came to the conclusion that the approach does not lead to good results. Then we investigated more carefully the condition (ii). The result of this investigation is the heuristic for cluster selection that we present below and which is based on the evaluation of the absolute value of the Pearson correlation between the pairs of columns of the matrix Z.

For ease of exposition, we introduce the following notation. If $W\in R^{p\times p}$ is symmetric, then $\alpha \left(W\right)=\sum_{1\leq i<j\leq p}\left|w_{ij}\right|$, where $|w_{ij}|$ is the magnitude of the entry of W located at the intersection of *i*-th row and the *j*-th column. Let $C\in R^{K\times K}$ be the matrix of the pairwise correlations between the columns of Z. For a subset $\Phi^{\left(g\right)}\subset \left\{1,\ldots ,K\right\}$ whose cardinality equals K/2, we take $C^{\left(g\right)}$ to be the block of C that corresponds to the rows and the columns indexed by $\Phi^{\left(g\right)}$. Then we select Φ as follows:

(12) $$\begin{array}{c} \Phi =\underset{\Phi^{\left(g\right)}}{\mathrm{argmin}}\left|\alpha \left(C^{\left(g\right)}\right)-\frac{1}{2}\alpha \left(C\right)\right|. \end{array}$$

We note that the cardinality of $\bar{\Phi }$ is also K/2. The formula implies that we find Φ with the property that the sum of the absolute pairwise correlations of the time series in cluster Φ is as close as possible to the sum of the absolute pairwise correlations of the time series in cluster $\bar{\Phi }$ plus the sum of the absolute correlations of the pairs that contain a time series from Φ and a time series from $\bar{\Phi }$. Remark that, in the particular case when the correlations for all the pairs that contain a time series from Φ and a time series from $\bar{\Phi }$ are zero, the sum of the absolute pairwise correlations of the time series in cluster Φ are approximately equal to the sum of the absolute pairwise correlations of the time series in cluster $\bar{\Phi }$. This approach has two limitations: (i) it can be applied only when the number of groups is two, and (ii) the computational burden is too high when *K* is large.

An alternative solution is a greedy algorithm which can be employed when the number of groups, $K^{\left(c\right)}$, is greater than two. For simplicity, we assume that *K* is a multiple integer of $K^{\left(c\right)}$. The algorithm constructs the groups as follows. Initially, the two time series that have the largest absolute correlation are included in the first group $\Phi_{1}$. In other words, we take

$$\begin{array}{c} \left(\phi_{1},\phi_{2}\right)=\underset{1\leq i<j\leq K}{\mathrm{argmax}}\left|c_{ij}\right|, \end{array}$$

and then $\Phi_{1}=\left\{\phi_{1},\phi_{2}\right\}$. At the next step, it is included in $\Phi_{1}$ the time series that increases the sum of the absolute correlations in the group the most:

$$\begin{array}{c} \phi_{3}=\underset{i\in \left\{1,\ldots ,K\right\}\setminus \Phi_{1}}{\mathrm{argmax}}\left(|c_{i\phi_{1}}\left|+\right|c_{i\phi_{2}}|\right), \end{array}$$

and $\Phi_{1}$ becomes $\Phi_{1}=\left\{\phi_{1},\phi_{2},\phi_{3}\right\}$. In general, after it was decided that $\Phi_{1}=\left\{\phi_{1},\ldots ,\phi_{r}\right\}$, where $2\leq r<K/K^{\left(c\right)}$, the (r+1)th time series is selected as follows:

$$\begin{array}{c} \phi_{r+1}=\underset{i\in \left\{1,\ldots ,K\right\}\setminus \Phi_{1}}{\mathrm{argmax}}\sum_{q=1}^{r}\left|c_{i\phi_{q}}\right|. \end{array}$$

Once the first cluster is built, the second one is initialized with the two time series from $\left\{1,\ldots ,K\right\}\setminus \Phi_{1}$ that have the largest absolute correlation, and the steps described above are applied for obtaining the second cluster. The procedure continues until $K^{\left(c\right)}-1$ groups are produced. The last group results automatically.

## 6. Experimental Settings

### 6.1. Simulation of the Missing Data

When we conduct experiments on a real-life multivariate time series $Z\in R^{T\times K}$, we randomly select the positions of the missing data. The selection is performed such that the number of missing data, $M_{\mathrm{miss}}$, is the same for each of the *K* time series. Hence, all the sets $\Psi_{1},\ldots ,\Psi_{K}$ have the same cardinality. It follows that the percentage of missing data is

(13) $$\begin{array}{c} \rho =100\frac{M_{\mathrm{miss}}}{T} \end{array}$$

for each time series. In our experiments, we consider ρ=5%, ρ=10%, ρ=15% and ρ=20%.

The indexes of the missing data for a particular time series are independent of the positions of the missing data in the other time series from the same data set. They are selected by either sampling without replacement $M_{\mathrm{miss}}$ integers from the set {1,…,T} or by using the Polya urn model (with finite memory), which was introduced in [36]. The Polya urn model is well-known, and it was employed in various applications. Some of these applications are: modeling of the communication channels [36,37,38,39], image segmentation [40] and modeling of epidemics on networks [41]. In [15], we have proposed the use of the Polya urn model for simulating the missing data in time series.

In the Polya model, the urn initially contains *R* red balls and *S* black balls (R<S). At each time moment t≥1, a ball is drawn from the urn and, after each draw, (1+Δ) balls of the same color as the drawn ball are returned to the urn. We take Δ>0. More details about the selection of Δ are provided below after the presentation of the most important properties of the model. Since we want the model to have finite memory, the experiment is performed as described above only for 1≤t≤M, where the parameter *M* is a positive integer (see the discussion in [36]). At each time moment t>M, a ball is drawn from the urn and, after each draw, two operations are executed: (i) (1+Δ) balls of the same color as the drawn ball are returned to the urn and (ii) Δ balls of the same color as the ball picked at time t−M are removed from the urn.

A sequence of random variables ${\left\{\Xi_{t}\right\}}_{1\leq t\leq T}$ is defined as follows: $\Xi_{t}=1$ if the ball drawn at time *t* is red and $\Xi_{t}=0$ if the ball drawn at time *t* is black. It was proven in [36,39] that the sequence $\left\{\Xi_{t}\right\}$ is a Markov process of order *M*. For t>M, let ${\underline{S}}_{t}$ denote the state $\left(\Xi_{t-M},\ldots ,\Xi_{t-1}\right)$. The Polya urn model has the remarkable property that the probability of having $\Xi_{t}=1$ after the state ${\underline{S}}_{t}$ was observed depends on the number of ones in $\left(\Xi_{t-M},\ldots ,\Xi_{t-1}\right)$, but not on their locations. We mention that M=5 in our settings.

The indexes of the missing data correspond to the positions of ones in the sequence ${\left\{\Xi_{t}\right\}}_{1\leq t\leq T}$. It is known that $P\left(\Xi_{t}=1\right)=\frac{R}{R+S}$, where the symbol P(·) denotes probability. In our simulations, *R* and *S* are chosen such that $P\left(\Xi_{t}=1\right)=\frac{M_{\mathrm{miss}}}{T}$. According to [39], the correlation $\mathrm{Corr}(\Xi_{t},\Xi_{t-i})$ is equal to $\frac{\delta }{1+\delta }$, where 0<i<M and $\delta =\frac{\Delta }{R+S}$. This property allows us to simulate bursts of missing data by taking δ=1. Obviously, this is different from the situation when the sampling without replacement is applied and when it is more likely to have isolated missing data. At the same time, the simulation of the missing data by using the Polya urn model is different from the approach in [2], where blocks of missing data are considered.

### 6.2. Data Pre-Processing

After the missing values are simulated, each time series is decomposed into trend, seasonal component and remainder. Then the DLM imputation method is applied on the T×K matrix of the remainder components. For each time series, both the trend and the seasonal components are added to the estimates produced by DLM to obtain the estimates for the missing data.

The decomposition uses the implementation for the R package imputeTS [42,43], which is available at https://github.com/SteffenMoritz/imputeTS/blob/master/R/na_seadec.R (accessed on 28 February 2022). The implementation returns a specific output when it cannot detect a seasonal pattern for the analyzed time series. From the package imputeTS, we only use the decomposition technique and not the imputation methods because all the imputation methods are designed for univariate time series; thus they are sub-optimal for the multivariate case.

### 6.3. Performance Evaluation

Let z be a column of the data matrix $Z\in R^{T\times K}$. We collect in the vector $z_{\bar{\Psi }}$ the entries of the time series z that are indexed by the elements of the set $\bar{\Psi }$. With the convention that ${\hat{z}}_{\bar{\Psi }}$ is the vector of estimates produced by DLM for the missing values of z, we calculate the following normalized error:

(14) $$\begin{array}{c} E=\frac{\left|\right|z_{\bar{\Psi }}-{\hat{z}}_{\bar{\Psi }}{\left|\right|}_{2}}{\left|\right|z_{\bar{\Psi }}{\left|\right|}_{2}}. \end{array}$$

The normalized errors are computed similarly for the estimates yielded by the imputation methods from [2]. To rank the methods, we calculate scores as follows. For each time series z, the imputation method that achieves the minimum normalized error yields two points, the method that leads to the second smallest normalized error yields one point, and all other methods yield zero points. The number of points accumulated by each method from the experiment with all time series in Z are divided by 2K to ensure that the scores take values in the interval [0,1].

When the imputation is performed by using ${\mathrm{DLM}}_{1}$, the expression in (14) is replaced with

(15) $$\begin{array}{c} E_{1}=\frac{\left|\right|z_{\bar{\Psi }}-{\hat{z}}_{\bar{\Psi }}{\left|\right|}_{1}}{\left|\right|z_{\bar{\Psi }}{\left|\right|}_{1}}, \end{array}$$

and the scores are calculated as explained above.

In the empirical comparison of the methods, we have used the code available at https://github.com/eXascaleInfolab/bench-vldb20.git (accessed on 3 October 2021) for the imputation methods that have been assessed in [2]. Short descriptions of these methods have been given in Section 1.1.

In the next section, we present the results obtained by DLM on five data sets that have been also used in [2]. For the sake of conciseness, we report the scores for ${\mathrm{DLM}}_{1}$ only for three data sets. The experimental results can be reproduced by using the Matlab code available at https://www.stat.auckland.ac.nz/%7Ecgiu216/PUBLICATIONS.htm (accessed on 17 June 2022).

## 7. Experimental Results

### 7.1. Climate Time Series (K = 10, T = 5000)

The data set comprises monthly climate measurements that have been recorded at various locations in North America from 1990 to 2002. We do not transform the time series with the method from Section 6.2 because it does not improve the quality of the imputation. As *K* is relatively small, we cluster the time series into $K^{\left(c\right)}=2$ groups that are found by using (12): Φ={5,6,7,8,10} and $\bar{\Phi }=\left\{1,2,3,4,9\right\}$. It is interesting that the rule in (12) yields the same grouping for all percentages of the missing data, for both sampling without replacement and for the Polya model.

Since the data are sampled monthly, it is natural to take the signal length m=12. We have $n_{u}\in \left\{5\times 2m,5\times 3m,5\times 4m\right\}$, and for each value of $n_{u}$, we take $n_{3}\in \left\{5\times m,5\times 2m,\ldots ,n_{u}-5\times m\right\}$. Observe that $n_{3}=n_{K^{\left(c\right)}+1}$, and it denotes the size of the block of the structured dictionary which contains atoms that are common for all time series. The sparsity level *s* is selected from the set {2,3,4}. It follows that the total number of triples $\left(n_{u},n_{3},s\right)$ that we consider is 18.

**Figure 1:**
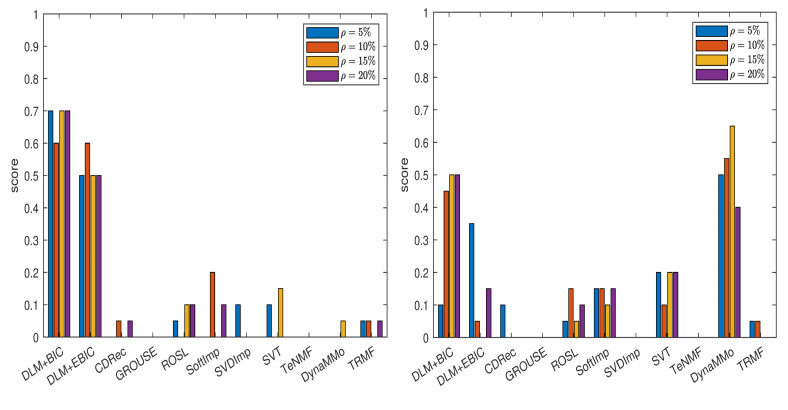
Scores for various imputation methods applied to the Climate time series: The missing data are simulated by sampling without replacement (**left** panel) and by using the Polya model (**right** panel). Note that the DLM algorithm is used.

**Figure 2:**
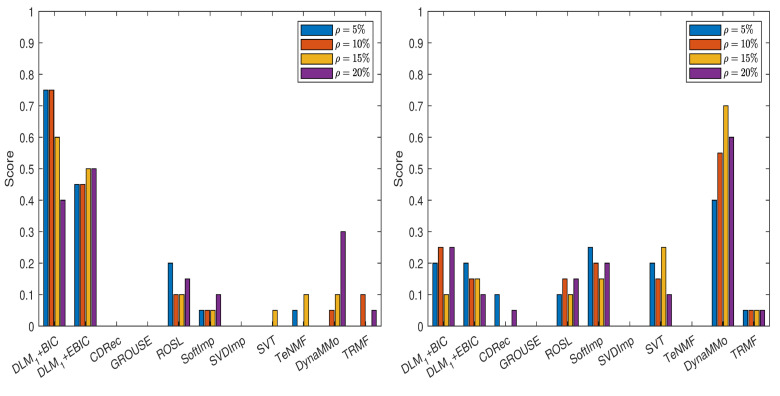
Scores for various imputation methods applied to the Climate time series: The missing data are simulated by sampling without replacement (**left** panel) and by using the Polya model (**right** panel). Note that the ${\mathrm{DLM}}_{1}$ algorithm is used.

We compute the normalized errors (see Table A1, Table A2, Table A3, Table A4, Table A5, Table A6, Table A7 and Table A8 in Appendix A.1.1), which lead to the scores shown in Figure 1. From the plot in the left panel of the figure, it is evident that both DLM+BIC and DLM+EBIC work better than other methods when the missing data are simulated by sampling without replacement. The method DLM+BIC is slightly superior to DLM+EBIC for all missing percentages, except for ρ=10%. In the right panel of the figure, where the Polya urn model is employed for simulating the missing data, we observe the following: the method DynaMMo is ranked the best for all missing percentages, except for ρ=20%, where DLM+BIC works better than DynaMMo. In Figure 2, we can see that DLM_1_+BIC and DLM_1_+EBIC are also very good when sampling without replacement is employed, but their performance diminishes in the case of the Polya model (for more details, see Table A9, Table A10, Table A11, Table A12, Table A13, Table A14, Table A15 and Table A16 in Appendix A.1.2).

### 7.2. Meteoswiss Time Series (K = 10, T = 10,000)

The measurements represent weather data collected in Swiss cities from 1980 to 2018. After simulating the missing data, we remove both the trend and the seasonal components for each time series (see Section 6.2). The transformed time series are clustered into $K^{\left(c\right)}=2$ groups as follows: Φ={1,2,3,4,5} and ${\bar{\Phi }}_{=}\left\{6,7,8,9,10\right\}$ (see (12)).

As the time interval between successive observations of these time series is 10 min, the most suitable value for *m* would be 6×24=144 (which corresponds to 24 h), but this makes the computational complexity too high. For keeping the computational burden at a reasonable level, we conduct experiments for three different values of *m*: m=6×4=24, m=6×6=36 and m=6×8=48. Note that each value of *m* corresponds to a time interval (in hours) that is a divisor of 24. For each value of *m*, $n_{u}$ is selected from the set {5×2m,5×3m,5×4m} and for each value of $n_{u}$, we have that $n_{3}\in \left\{5\times m,5\times 2m,\cdots ,n_{u}-5\times m\right\}$. The sparsity level *s* is chosen from the set {3,4,6}. We use these settings for the case when the missing data are generated by sampling without replacement and ρ=5%. The results can be found in Table A17. It can be easily noticed that both DLM+BIC and DLM+EBIC yield very good results for all values of *m*.

**Figure 3:**
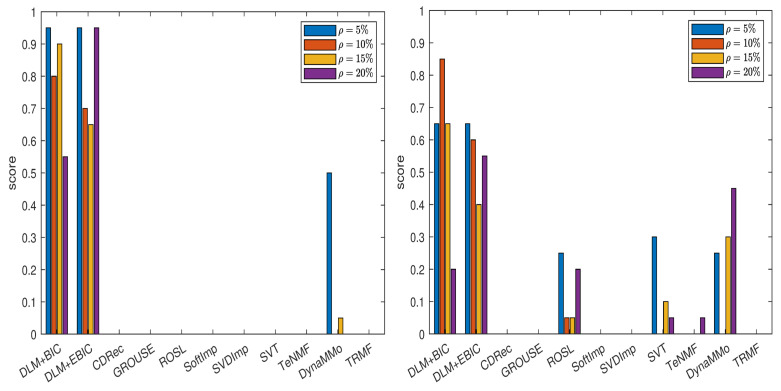
Scores for various imputation methods applied to the MeteoSwiss time series: The missing data are simulated by sampling without replacement (**left** panel) and by using the Polya model (**right** panel). Note that the DLM algorithm is used.

**Figure 4:**
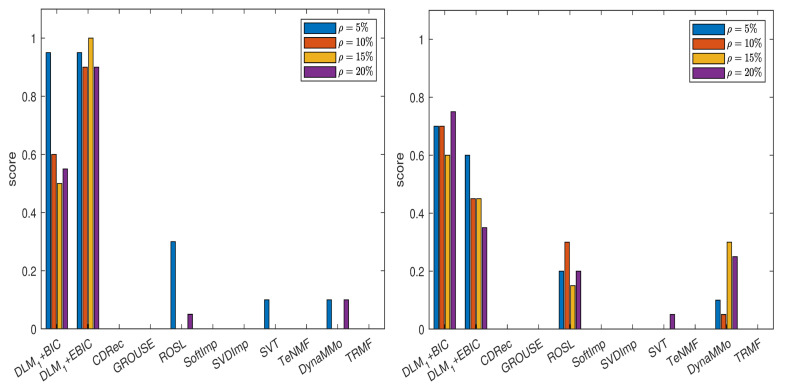
Scores for various imputation methods applied to the MeteoSwiss time series: The missing data are simulated by sampling without replacement (**left** panel) and by using the Polya model (**right** panel). Note that the ${\mathrm{DLM}}_{1}$ algorithm is used.

Taking into consideration these results, we further conduct the experiments for all cases of missing data simulations by setting m=24 and s=3. Hence, we have six candidates for $\left(n_{u},n_{3},s\right)$. The grouping for which Φ={1,2,3,4,5} is employed. The results are reported in Table A18, Table A19, Table A20, Table A21, Table A22, Table A23, Table A24 and Table A25, in Appendix A.2.1, and in Figure 3. Both DLM+BIC and DLM+EBIC have outstanding performance for sampling without replacement, and they are very good when the Polya model is used. In the latter case, DLM+BIC is less successful when ρ=20%. According to the scores shown in Figure 4, which are based on Table A26, Table A27, Table A28, Table A29, Table A30, Table A31, Table A32 and Table A33 (see Appendix A.2.2), the ranking of the imputation methods does not change significantly when the algorithm DLM is replaced with ${\mathrm{DLM}}_{1}$.

### 7.3. BAFU Time Series (K = 10, T = 50,000)

These are water discharge time series recorded by BAFU (BundesAmt Für Umwelt - Swiss Federal Office for the Environment) on Swiss rivers from 2010 to 2015. We do not remove the seasonality from the simulated incomplete time series because the method from [42] does not detect seasonal components for 3 out of 10 time series. Hence, the imputation is performed on the original time series. The grouping Φ={1,3,6,7,9} and $\bar{\Phi }=\left\{2,4,5,8,10\right\}$ is used to cluster the time series into $K^{\left(c\right)}=2$ groups when the missing data are simulated by sampling without replacement (for all missing percentages) or by the Polya model with ρ=15%. For the other three cases of missing data simulations, the grouping Φ={2,3,6,7,9} and $\bar{\Phi }=\left\{1,4,5,8,10\right\}$ is applied.

Relying on some of the empirical observations that we made for the MeteoSwiss time series and taking into consideration the fact that the sampling period for the BAFU time series is equal to 30 min, we set m=12 (which corresponds to a time interval of 6 h). The possible values for $n_{u}$ and $n_{3}$ are calculated by using the same formulas as in the experiments with climate and MeteoSwiss time series. In those experiments, we have noticed that the small values are preferred for the sparsity level, hence we take s=3. This implies that the number of candidates for $\left(n_{u},n_{3},s\right)$ is the same as in the case of MeteoSwiss time series.

**Figure 5:**
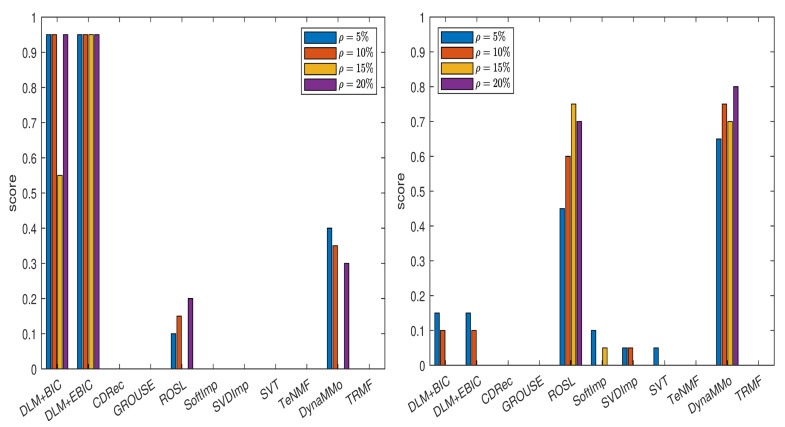
Scores for various imputation methods applied to the BAFU time series: The missing data are simulated by sampling without replacement (**left** panel) and by using the Polya model (**right** panel).

The normalized errors for this data set are given in Appendix A.3 (see Table A34, Table A35, Table A36, Table A37, Table A38, Table A39, Table A40 and Table A41). In Figure 5, we show the scores computed for various imputation methods. From the left panel of the figure, it is clear that both DLM+BIC and DLM+EBIC are the best for ρ=5%,10%,20%, where their scores are approximately one. The imputation method DLM+BIC does not work as well as DLM+EBIC when the percentage of the missing data is ρ=15%. In the right panel of the figure, we observe that both DLM+BIC and DLM+EBIC have modest performance when the missing data are simulated by the Polya model. In this case, DynaMMo and **ROSL** are the winners.

### 7.4. Temperature Time Series (K = 50, T = 5000)

The data set contains average daily temperatures recorded at various sites in China from 1960 to 2012. After removing the trend and the seasonal components from all 50 time series with simulated missing data, we cluster them by applying the greedy algorithm which was introduced in Section 5.2. The resulting groups of time series are listed in Table A42 (see Appendix A.4). Note that the total number of groups is $K^{\left(c\right)}=5$, and there are 10 time series in each group. It is interesting that the way in which the time series are assigned to the clusters depends very much on the method employed for simulating the missing data and on the percentage of missing data.

Mainly based on the lessons learned from the experiments with the data sets that have been analyzed in the previous sections, we have decided to take the signal length m=12. We opt for relatively small values for $n_{u}$, thus $n_{u}$ is selected from the set $\left\{10\times \frac{1}{2}m,10\times m,10\times \frac{3}{2}m\right\}$. For $n_{u}=10\times \frac{1}{2}m$, we have that $n_{6}=n_{u}$, which means that all the atoms are common for all the time series, and atoms that are specific for a certain group do not exist. For the other two values of $n_{u}$, we allow $n_{6}$ to be selected from the set $\left\{n_{u}-5m,n_{u}\right\}$. The sparsity level *s* is selected from the set {2,3}. Simple calculations lead to the conclusion that the total number of triples $\left(n_{u},n_{6},s\right)$ equals 10.

**Figure 6:**
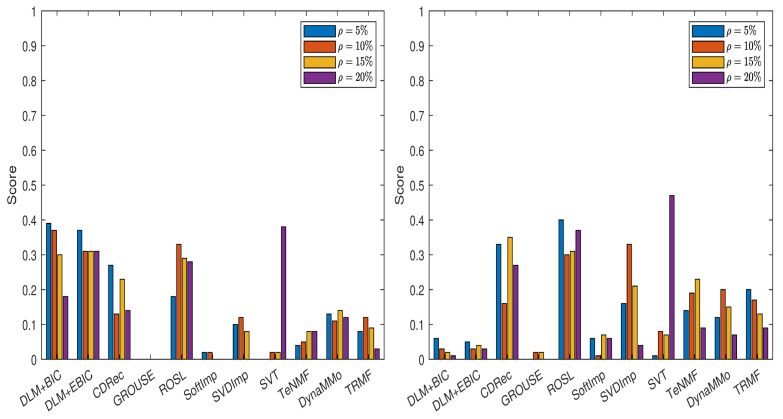
Scores for various imputation methods applied to the Temperature time series: The missing data are simulated by sampling without replacement (**left** panel) and by using the Polya model (**right** panel).

The normalized errors from Table A43, Table A44, Table A45, Table A46, Table A47, Table A48, Table A49 and Table A50 (see Appendix A.4) lead to the scores displayed in Figure 6. For the case of sampling without replacement, DLM+BIC, DLM+EBIC and ROSL have similar performance, and they are followed by CDRec. It is interesting that although SVT has a very modest rank when the percentage of the missing data is small, it becomes superior to all other methods when ρ=20%. As we have already observed for the BAFU time series, DLM+BIC and DLM+EBIC are not well ranked when the data are simulated by the Polya model. The difference compared with the results for the BAFU time series comes from the fact that ROSL and DynaMMo are not clear winners. This time, ROSL and CDRec are better than the other competitors. Similar to the case when the missing data are simulated by sampling without replacement, SVT outperforms all other imputation methods when ρ=20%.

### 7.5. Air Time Series (K = 10, T = 1000)

The data set comprises hourly sampled air quality measurements that have been recorded at monitoring stations in China from 2014 to 2015. For the case when the missing data are simulated by sampling without replacement (with ρ=5%,10%,15%) and the Polya model (with ρ=5%,10%,20%), the groups produced by the criterion in (12) are Φ={2,3,5,9,10} and $\bar{\Phi }=\left\{1,4,6,7,8\right\}$. For the other two cases of missing data simulations, the clustering is: Φ={1,2,4,5,9} and $\bar{\Phi }=\left\{3,6,7,8,10\right\}$. We do not remove the trend and seasonal components since, for each missing data simulation, the seasonal patterns are not detected in more than half of the time series.

We have applied DLM and ${\mathrm{DLM}}_{1}$ algorithms on this data set. In both cases, we take the signal length m=24 because we analyze hourly data. The parameter $n_{u}$ is selected from the set {5×2m,5×3m,5×4m}. For each value of $n_{u}$, we have that $n_{3}\in \left\{5\times m,5\times 2m,\ldots ,n_{u}-5\times m\right\}$. The sparsity level *s* is selected from the set {2,3,4}.

**Figure 7:**
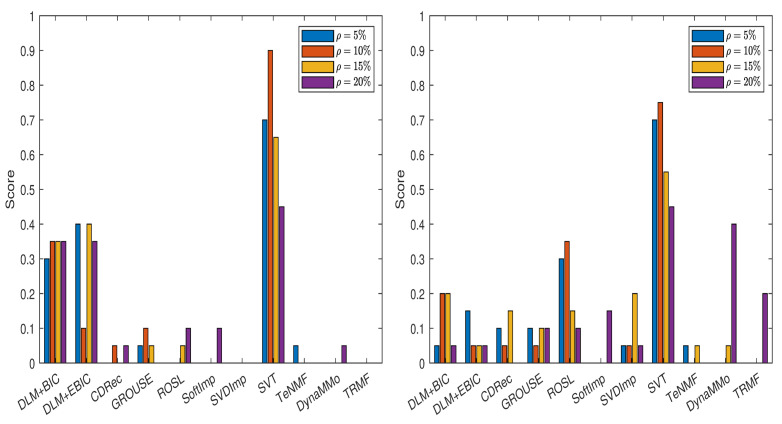
Scores for various imputation methods applied to the Air time series: The missing data are simulated by sampling without replacement (**left** panel) and by using the Polya model (**right** panel). Note that the DLM algorithm is used.

**Figure 8:**
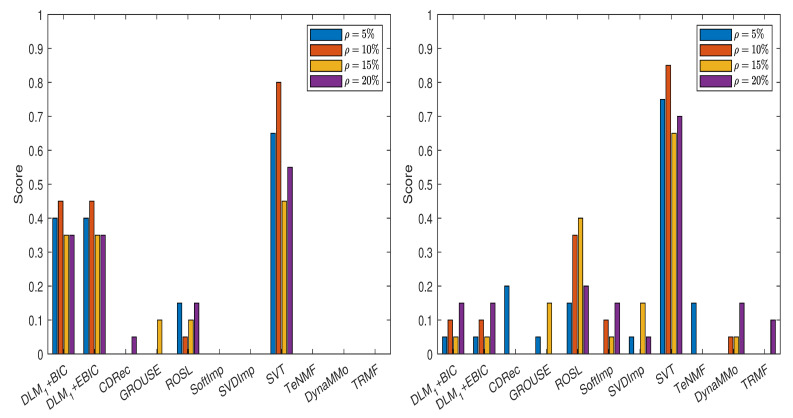
Scores for various imputation methods applied to the Air time series: The missing data are simulated by sampling without replacement (**left** panel) and by using the Polya model (**right** panel). Note that the ${\mathrm{DLM}}_{1}$ algorithm is used.

In Figure 7, we show the scores obtained by various imputation methods when the DLM algorithm is applied, and the normalized errors are computed with the formula from (14), see also Table A51, Table A52, Table A53, Table A54, Table A55, Table A56, Table A57 and Table A58 in Appendix A.5.1. It is evident that both DLM+BIC and DLM+EBIC have better performance when sampling without replacement is employed to simulate the missing data. SVT is clearly the best method for both cases of missing data simulations and for all percentages ρ. The ranking of the imputation methods is the same in Figure 8, where the scores have been calculated by using (15), see Table A59, Table A60, Table A61, Table A62, Table A63, Table A64, Table A65 and Table A66 in Appendix A.5.2. An important difference between the two figures is that ${\mathrm{DLM}}_{1}$ and the corresponding IT criteria are employed in Figure 8.

## 8. Final Remarks

In this work, we have exemplified how two techniques for dimensionality reduction can be employed for extending the use of the DLM algorithm to data sets that contain tens of time series. It remains to be investigated by the future research how the previous results on time series clustering can be utilized for grouping the time series and finding the number of groups (see, for example [44], for the definition of a metric that evaluates the dissimilarity between two time series). We have also derived the novel ${\mathrm{DLM}}_{1}$ algorithm and the corresponding BIC and EBIC criteria. We have conducted a large set of experiments for comparing DLM and ${\mathrm{DLM}}_{1}$ with nine algorithms that represent the state-of-the-art in the multivariate time series imputation.

Our empirical study confirms a fact already observed in the previous literature: There is no imputation method which always yields the best results. Although not always the best, our method clearly outperforms the other studied methods for some missing data models. So, it appears to be a useful addition to the existing methods. DLM tends to work better when the missing data are isolated (see the results for simulation by sampling without replacement) than in the case when the sequences of missing data are long (see the results for simulation by using the Polya model).

Both BIC and EBIC are effective in selecting the best structure of the dictionary and the sparsity. It is interesting that small values are chosen for the sparsity *s*, and this supports the idea that sparse models are appropriate for the multivariate time series. The values selected for $n_{u}$ are also relatively small. Recall that $n_{u}$ equals the number of atoms used in the representation of a specific time series (or a group of time series) plus the number of atoms that are common for all the time series in the data set.

Our imputation method can also be applied when the percentage of the missing data is not the same for all the time series in the data set. Based on the experimental results, it is easy to see that the percentage of missing data does not have an important influence on how DLM is ranked in comparison with other methods; thus we expect the same to be true when the number of missing data varies across the time series.

## Data Availability

Not applicable.

## Appendix A

The tables below contain the normalized errors (see (14) and (15)) for the imputation methods that are assessed in this work. Note that the errors are computed for each time series in the data sets used in the empirical study. For each row in the tables, the best result is written in red and the second best result is written with bold font. For the methods DLM and ${\mathrm{DLM}}_{1}$, we also show the values of the triple ($n_{u}$, $n_{K+1}$, *s*) selected by BIC (see (9) and (11)) and EBIC (10). In the caption of each table, we provide information about the missing data: how they have been simulated and what is the value of ρ (13). The only exception is Table A42 in Appendix A.4, which does not contain normalized errors, but shows the clustering of the time series.

### Appendix A.1. Climate Time Series: Numerical Results

#### Appendix A.1.1. Results Obtained by Using DLM

**Table A1:** Climate time series: Sampling without replacement (ρ=5%).

Time	DLM+BIC	DLM+EBIC	CDRec	GROUSE	ROSL	SoftImp	SVDImp	SVT	TeNMF	DynaMMo	TRMF
Series	$n_{u}$ = 240	$n_{u}$ = 240									
	s = 2	s = 2									
	$n_{3}$ = 60	$n_{3}$ = 180									
TS1	0.2206	0.2378	0.9076	0.9206	1.1368	0.8791	0.8968	0.7794	0.9739	0.5496	0.8835
TS2	0.3859	0.4220	0.6436	0.7692	0.7993	0.6225	0.6357	0.6140	0.6931	0.5437	0.6271
TS3	0.4527	0.4836	0.9099	0.9346	0.8571	0.8732	0.8811	0.7824	0.8789	0.7286	0.8764
TS4	0.3806	0.4138	0.9901	1.5210	1.0613	1.0005	1.0104	0.8415	0.9580	0.8624	1.0043
TS5	0.5997	0.6453	0.5979	0.7721	0.5650	0.5862	0.5918	0.5597	0.5723	0.6048	0.5894
TS6	0.4955	0.5227	0.6212	0.7566	0.5856	0.6037	0.6057	0.6812	0.6690	0.5529	0.6027
TS7	0.1928	0.2078	0.6914	0.8858	0.7510	0.6683	0.6746	0.7331	1.0271	0.5201	0.6696
TS8	0.7010	0.7225	0.6346	0.7127	0.6369	0.6260	0.6252	0.6748	0.6424	0.6557	0.6253
TS9	0.4412	0.4232	0.9046	1.0713	0.9132	0.9305	0.9432	0.8629	1.0128	0.7983	0.9340
TS10	0.5064	0.4963	0.7300	0.9366	0.8476	0.7216	0.7288	0.7219	1.1201	0.6585	0.7245

**Table A2:** Climate time series: Sampling without replacement (ρ=10%).

Time	DLM+BIC	DLM+EBIC	CDRec	GROUSE	ROSL	SoftImp	SVDImp	SVT	TeNMF	DynaMMo	TRMF
Series	$n_{u}$ = 240	$n_{u}$ = 240									
	s = 2	s = 2									
	$n_{3}$ = 60	$n_{3}$ = 180									
TS1	0.3141	0.3079	0.8854	0.9501	1.1632	0.8625	0.8793	0.7762	0.9942	0.5658	0.8676
TS2	0.3910	0.4051	0.6454	0.8826	0.8076	0.6152	0.6312	0.6144	0.7761	0.5334	0.6226
TS3	0.4310	0.4611	1.0124	0.9482	0.8484	0.9483	0.9636	0.7913	0.9159	0.7569	0.9514
TS4	0.3739	0.4019	0.9442	1.2165	1.1301	0.9557	0.9660	0.8399	1.1305	0.8120	0.9574
TS5	0.6285	0.6536	0.5630	0.7562	0.5655	0.5552	0.5575	0.5868	0.5659	0.5763	0.5556
TS6	0.5302	0.5295	0.6570	1.0571	0.6188	0.6430	0.6481	0.6910	0.6838	0.5787	0.6439
TS7	0.1716	0.1882	0.7024	0.8766	0.7107	0.6760	0.6866	0.7225	1.0463	0.5045	0.6772
TS8	0.7443	0.7381	0.6309	0.7141	0.6395	0.6299	0.6344	0.6453	0.6517	0.6434	0.6321
TS9	0.4504	0.4478	0.9088	1.0934	0.9006	0.9515	0.9659	0.8561	1.0037	0.7834	0.9546
TS10	0.5064	0.4863	0.7862	0.9547	0.7982	0.7744	0.7803	0.7735	1.2519	0.7048	0.7760

**Table A3:** Climate time series: Sampling without replacement (ρ=15%).

Time	DLM+BIC	DLM+EBIC	CDRec	GROUSE	ROSL	SoftImp	SVDImp	SVT	TeNMF	DynaMMo	TRMF
Series	$n_{u}$ = 240	$n_{u}$ = 240									
	s = 2	s = 2									
	$n_{3}$ = 60	$n_{3}$ = 180									
TS1	0.2762	0.2636	0.9645	1.0027	1.1502	0.8850	0.9142	0.7859	0.9377	0.5533	0.9007
TS2	0.4363	0.4590	0.7113	1.0861	0.8171	0.6471	0.6697	0.6635	0.7396	0.5612	0.6638
TS3	0.4786	0.5362	1.1201	0.9592	0.8838	0.9889	1.0180	0.8024	0.9362	0.7771	1.0032
TS4	0.4216	0.4314	0.9939	1.6014	1.0838	1.0061	1.0198	0.8520	1.0660	0.8295	1.0139
TS5	0.6733	0.6931	0.6606	0.9957	0.6302	0.6377	0.6517	0.6237	0.6289	0.6256	0.6497
TS6	0.4903	0.5191	0.6771	0.9616	0.5994	0.6407	0.6543	0.6831	0.6865	0.5465	0.6496
TS7	0.2039	0.2113	0.7841	0.8774	0.7193	0.7026	0.7239	0.7247	0.9732	0.5125	0.7212
TS8	0.7403	0.7623	0.6706	0.8888	0.6265	0.6628	0.6738	0.6560	0.6814	0.6598	0.6732
TS9	0.5364	0.5081	0.9338	1.0657	0.9134	0.9537	0.9737	0.8587	1.0472	0.8068	0.9665
TS10	0.4992	0.5110	0.7761	1.1871	0.8277	0.7526	0.7604	0.7704	1.1942	0.6669	0.7602

**Table A4:** Climate time series: Sampling without replacement (ρ=20%).

Time	DLM+BIC	DLM+EBIC	CDRec	GROUSE	ROSL	SoftImp	SVDImp	SVT	TeNMF	DynaMMo	TRMF
Series	$n_{u}$ = 240	$n_{u}$ = 240									
	s = 2	s = 2									
	$n_{3}$ = 60	$n_{3}$ = 180									
TS1	0.2684	0.2989	1.0392	1.2251	1.1457	0.8858	0.9379	0.8019	0.9823	0.5830	0.9257
TS2	0.4676	0.4778	0.8930	1.4169	0.8577	0.7134	0.7775	0.6847	0.8436	0.5846	0.7721
TS3	0.4978	0.5197	1.0142	0.9721	0.8461	0.9177	0.9422	0.8207	0.9307	0.7349	0.9261
TS4	0.4291	0.4642	1.0404	1.6239	1.0929	0.9672	1.0113	0.8516	1.2570	0.7892	1.0085
TS5	0.6579	0.6485	0.5818	0.8984	0.6054	0.5689	0.5759	0.6225	0.5911	0.5892	0.5719
TS6	0.5604	0.5611	0.6622	0.9214	0.6053	0.6393	0.6498	0.7008	0.6920	0.5674	0.6425
TS7	0.2014	0.2298	0.7425	0.8486	0.7410	0.7011	0.7237	0.7442	1.0899	0.5137	0.7074
TS8	0.7687	0.7896	0.6668	0.7766	0.6605	0.6699	0.6760	0.7012	0.6939	0.6744	0.6722
TS9	0.5123	0.5026	0.9051	1.1512	0.9104	0.9247	0.9470	0.8688	1.0367	0.7791	0.9312
TS10	0.5210	0.5197	0.7173	1.0914	0.8534	0.7105	0.7172	0.7688	1.3176	0.6497	0.7123

**Table A5:** Climate time series: Polya model (ρ=5%).

Time	DLM+BIC	DLM+EBIC	CDRec	GROUSE	ROSL	SoftImp	SVDImp	SVT	TeNMF	DynaMMo	TRMF
Series	$n_{u}$ = 240	$n_{u}$ = 240									
	s = 2	s = 2									
	$n_{3}$ = 60	$n_{3}$ = 180									
TS1	1.5339	0.5999	1.0875	0.9079	1.0849	1.0033	1.0402	0.7438	0.9397	0.6961	1.0238
TS2	0.5148	0.5452	0.5847	0.8760	0.8409	0.5870	0.6050	0.6449	0.7186	0.5397	0.5936
TS3	2.4690	0.8270	0.9446	0.9894	0.9088	0.8706	0.8825	0.7442	0.9026	0.7754	0.8759
TS4	1.5878	0.7257	0.9449	1.5592	1.0753	0.9622	0.9736	0.8660	1.0507	0.8517	0.9706
TS5	0.9075	0.8044	0.4867	0.6671	0.5474	0.4791	0.4821	0.5227	0.5037	0.5441	0.4805
TS6	2.8231	0.8023	0.6350	1.0170	0.6313	0.6155	0.6214	0.6638	0.6435	0.5985	0.6175
TS7	2.4865	0.6010	0.6483	0.8625	0.7472	0.6380	0.6443	0.7206	1.0270	0.5224	0.6376
TS8	2.6843	0.7976	0.5815	0.7659	0.5559	0.6009	0.6145	0.5403	0.6135	0.5830	0.6093
TS9	1.2003	0.7649	0.8788	1.0036	0.9431	0.9064	0.9186	0.8477	1.0531	0.8149	0.9097
TS10	2.1281	0.7243	0.5734	0.8083	0.8292	0.5865	0.5845	0.7346	0.9960	0.5809	0.5842

**Table A6:** Climate time series: Polya model (ρ=10%).

Time	DLM+BIC	DLM+EBIC	CDRec	GROUSE	ROSL	SoftImp	SVDImp	SVT	TeNMF	DynaMMo	TRMF
Series	$n_{u}$ = 240	$n_{u}$ = 240									
	s = 2	s = 2									
	$n_{3}$ = 60	$n_{3}$ = 180									
TS1	0.4906	1.0668	0.9704	0.8799	0.9600	0.9315	0.9523	0.7768	0.9242	0.6934	0.9396
TS2	0.8093	1.2025	0.6750	0.8925	0.8561	0.6409	0.6653	0.6263	0.7540	0.5569	0.6490
TS3	0.7706	1.1866	0.9941	0.9725	0.9234	0.9300	0.9471	0.8002	0.9133	0.7674	0.9396
TS4	0.7488	1.3568	0.9728	1.3394	1.0630	0.9883	1.0031	0.8413	1.1103	0.8606	0.9951
TS5	0.7862	0.7885	0.5766	0.7556	0.5999	0.5676	0.5732	0.5918	0.5703	0.5985	0.5689
TS6	0.7162	0.7336	0.6631	0.8485	0.6282	0.6425	0.6496	0.6843	0.6464	0.5855	0.6447
TS7	0.5634	0.5756	0.7029	0.7593	0.7823	0.6718	0.6845	0.7146	1.0417	0.5254	0.6798
TS8	0.8454	0.8214	0.6738	0.8109	0.6639	0.6713	0.6738	0.6864	0.6929	0.6891	0.6725
TS9	0.8455	1.1703	0.8723	0.9082	0.8947	0.8863	0.8975	0.8535	1.0771	0.8048	0.8900
TS10	0.5941	0.6240	0.7183	0.9915	0.8048	0.7061	0.7150	0.7308	1.2304	0.6546	0.7091

**Table A7:** Climate time series: Polya model (ρ=15%).

Time	DLM+BIC	DLM+EBIC	CDRec	GROUSE	ROSL	SoftImp	SVDImp	SVT	TeNMF	DynaMMo	TRMF
Series	$n_{u}$ = 240	$n_{u}$ = 240									
	s = 2	s = 2									
	$n_{3}$ = 60	$n_{3}$ = 180									
TS1	0.6623	1.0546	1.0798	1.0494	1.0891	0.9497	0.9925	0.7989	1.0618	0.7023	0.9922
TS2	0.7268	0.9331	0.8410	1.0543	0.8370	0.6970	0.7368	0.6759	0.8074	0.6127	0.7424
TS3	0.7846	0.7951	1.0928	0.9645	0.9332	0.9453	0.9920	0.8166	0.9172	0.7520	0.9678
TS4	0.7257	1.0285	0.9472	1.5868	1.0632	0.9127	0.9289	0.8620	1.1940	0.7932	0.9260
TS5	0.8505	0.8842	0.6870	1.1035	0.6246	0.6239	0.6502	0.6193	0.6271	0.6503	0.6464
TS6	0.7178	0.9969	0.6951	1.2827	0.6276	0.6476	0.6689	0.7163	0.7032	0.5953	0.6624
TS7	0.6162	0.6748	0.8578	0.9729	0.8338	0.7813	0.8334	0.7350	1.2379	0.5920	0.8106
TS8	0.8303	0.9381	0.7326	1.0616	0.7029	0.6996	0.7242	0.7087	0.7411	0.6986	0.7206
TS9	0.7807	1.0360	0.8964	0.9612	0.9182	0.9116	0.9321	0.8651	1.0274	0.8242	0.9203
TS10	0.7365	0.8499	0.9079	1.7080	0.9540	0.8317	0.8692	0.7640	1.5698	0.7800	0.8616

**Table A8:** Climate time series: Polya model (ρ=20%).

Time	DLM+BIC	DLM+EBIC	CDRec	GROUSE	ROSL	SoftImp	SVDImp	SVT	TeNMF	DynaMMo	TRMF
Series	$n_{u}$ = 240	$n_{u}$ = 180									
	s = 2	s = 2									
	$n_{3}$ = 60	$n_{3}$ = 120									
TS1	0.5773	0.6877	0.9661	1.2687	1.2944	0.8826	0.9185	0.8051	0.9959	0.6391	0.9124
TS2	0.7705	0.8007	0.7518	1.0678	0.7904	0.6526	0.6809	0.6765	0.8030	0.5922	0.6834
TS3	0.7502	0.8509	1.0267	2.2326	0.9222	0.9188	0.9434	0.8187	0.9178	0.7690	0.9332
TS4	0.7871	0.8449	0.9917	1.1806	1.0639	0.9661	0.9810	0.8695	1.0951	0.8549	0.9832
TS5	0.8635	0.8803	0.6819	0.7345	0.6824	0.6583	0.6823	0.6464	0.6904	0.6637	0.6724
TS6	0.7028	0.6949	0.6683	1.0010	0.6070	0.6446	0.6576	0.7205	0.7234	0.5778	0.6490
TS7	0.7877	0.7843	0.7533	0.7838	0.8042	0.6952	0.7162	0.7537	1.0247	0.5683	0.7089
TS8	0.8987	0.9251	0.7212	0.8163	0.6920	0.7161	0.7372	0.6817	0.7375	0.7080	0.7283
TS9	0.7284	0.8182	0.9767	1.0495	0.9317	0.9989	1.0330	0.8746	1.0663	0.8515	1.0227
TS10	0.6367	0.6441	0.7893	0.8229	0.8219	0.7869	0.7953	0.8108	1.2228	0.7414	0.7905

#### Appendix A.1.2. Results obtained by using DLM_1_

**Table A9:** Climate time series: Sampling without replacement (ρ=5%).

Time	DLM${}_{1}$+BIC	DLM${}_{1}$+EBIC	CDRec	GROUSE	ROSL	SoftImp	SVDImp	SVT	TeNMF	DynaMMo	TRMF
Series	$n_{u}$ = 240	$n_{u}$ = 240									
	s = 2	s = 2									
	$n_{3}$ = 60	$n_{3}$ = 180									
TS1	0.2103	0.2245	0.8594	0.8644	1.0938	0.8345	0.8504	0.7708	0.9375	0.5400	0.8382
TS2	0.3741	0.4063	0.5777	0.7492	0.7730	0.5638	0.5723	0.5789	0.6826	0.4795	0.5654
TS3	0.4323	0.4799	0.8821	0.9186	0.8096	0.8356	0.8456	0.7495	0.8233	0.6960	0.8412
TS4	0.3703	0.4176	0.9966	1.4368	1.0705	1.0074	1.0206	0.8364	0.9442	0.8654	1.0132
TS5	0.6057	0.6258	0.5398	0.7132	0.5191	0.5287	0.5324	0.5410	0.5253	0.5683	0.5305
TS6	0.5005	0.5038	0.6040	0.7690	0.5685	0.5852	0.5867	0.6705	0.6514	0.5247	0.5839
TS7	0.2160	0.2031	0.6627	0.8886	0.7282	0.6454	0.6509	0.7230	1.0134	0.5068	0.6463
TS8	0.6125	0.5818	0.5516	0.6489	0.5281	0.5484	0.5511	0.5843	0.5715	0.5583	0.5494
TS9	0.3788	0.3953	0.9311	1.0529	0.8486	0.9635	0.9831	0.8033	0.9673	0.8036	0.9730
TS10	0.4958	0.5023	0.6211	0.8362	0.7875	0.6140	0.6210	0.6490	1.0691	0.5668	0.6161

**Table A10:** Climate time series: Sampling without replacement (ρ=10%).

Time	DLM${}_{1}$+BIC	DLM${}_{1}$+EBIC	CDRec	GROUSE	ROSL	SoftImp	SVDImp	SVT	TeNMF	DynaMMo	TRMF
Series	$n_{u}$ = 240	$n_{u}$ = 180									
	s = 2	s = 2									
	$n_{3}$ = 120	$n_{3}$ = 120									
TS1	0.2338	0.2356	0.8180	0.8209	1.0948	0.7942	0.8112	0.7599	0.9390	0.5353	0.7995
TS2	0.4073	0.4233	0.5987	0.7377	0.7471	0.5692	0.5840	0.5803	0.7119	0.4818	0.5768
TS3	0.5076	0.5258	0.9633	0.9281	0.7897	0.9106	0.9225	0.7723	0.8824	0.7390	0.9121
TS4	0.4399	0.4535	0.9402	1.1680	1.1288	0.9535	0.9659	0.8336	1.0758	0.8072	0.9561
TS5	0.6227	0.6139	0.5027	0.6744	0.5100	0.4950	0.4956	0.5566	0.5235	0.5189	0.4943
TS6	0.5269	0.5277	0.6272	1.0220	0.5946	0.6131	0.6175	0.6720	0.6591	0.5482	0.6135
TS7	0.2757	0.2761	0.6433	0.8941	0.6438	0.6280	0.6353	0.7057	0.9870	0.4718	0.6277
TS8	0.6525	0.6611	0.5904	0.6758	0.5598	0.5950	0.6043	0.5809	0.6216	0.5782	0.6003
TS9	0.4141	0.4051	0.9199	1.0825	0.8304	0.9719	0.9941	0.8054	0.9861	0.7764	0.9798
TS10	0.4810	0.4832	0.7195	0.8663	0.7312	0.7081	0.7130	0.7263	1.1506	0.6364	0.7088

**Table A11:** Climate time series: Sampling without replacement (ρ=15%).

Time	DLM${}_{1}$+BIC	DLM${}_{1}$+EBIC	CDRec	GROUSE	ROSL	SoftImp	SVDImp	SVT	TeNMF	DynaMMo	TRMF
Series	$n_{u}$ = 240	$n_{u}$ = 180									
	s = 2	s = 2									
	$n_{3}$ = 60	$n_{3}$ = 120									
TS1	0.2964	0.2865	0.8764	0.8690	1.0359	0.8144	0.8400	0.7722	0.8833	0.5355	0.8261
TS2	0.4662	0.4741	0.6515	0.9666	0.7651	0.6076	0.6277	0.6294	0.7032	0.5048	0.6193
TS3	0.5636	0.5786	1.0268	0.9438	0.8117	0.9307	0.9531	0.7861	0.8972	0.7504	0.9419
TS4	0.5144	0.5263	1.0045	1.5289	1.0757	1.0182	1.0313	0.8510	1.0383	0.8365	1.0273
TS5	0.6999	0.6877	0.5847	0.7622	0.5742	0.5709	0.5788	0.5988	0.5708	0.5827	0.5765
TS6	0.5305	0.5333	0.6295	0.7617	0.5681	0.6049	0.6138	0.6657	0.6479	0.5229	0.6079
TS7	0.3366	0.3226	0.7021	0.8438	0.6814	0.6577	0.6717	0.7041	0.9264	0.4836	0.6658
TS8	0.6694	0.6622	0.6048	0.7121	0.5327	0.6122	0.6244	0.5860	0.6336	0.5922	0.6205
TS9	0.5185	0.4977	0.9411	1.0714	0.8446	0.9787	1.0035	0.8060	1.0395	0.8033	0.9936
TS10	0.5308	0.5367	0.6911	0.8891	0.7710	0.6771	0.6832	0.7206	1.1344	0.6040	0.6810

**Table A12:** Climate time series: Sampling without replacement (ρ=20%).

Time	DLM${}_{1}$+BIC	DLM${}_{1}$+EBIC	CDRec	GROUSE	ROSL	SoftImp	SVDImp	SVT	TeNMF	DynaMMo	TRMF
Series	$n_{u}$ = 240	$n_{u}$ = 120									
	s = 2	s = 2									
	$n_{3}$ = 60	$n_{3}$ = 60									
TS1	0.3706	0.3792	0.9186	0.9562	1.0449	0.8315	0.8681	0.7895	0.9111	0.5624	0.8532
TS2	0.5608	0.5602	0.7367	1.1844	0.8174	0.6524	0.6899	0.6425	0.7893	0.5266	0.6810
TS3	0.6398	0.6370	0.9544	0.9660	0.7708	0.8830	0.9026	0.8013	0.9030	0.7158	0.8892
TS4	0.5846	0.5704	0.9830	1.4834	1.1016	0.9605	0.9926	0.8393	1.1260	0.7708	0.9832
TS5	0.6947	0.6742	0.5389	0.8171	0.5584	0.5284	0.5323	0.5959	0.5513	0.5458	0.5298
TS6	0.5979	0.5866	0.6363	0.9182	0.5795	0.6184	0.6291	0.6839	0.6640	0.5424	0.6215
TS7	0.4053	0.4338	0.6913	0.8504	0.6790	0.6564	0.6774	0.7257	1.0044	0.4728	0.6636
TS8	0.7012	0.6935	0.6120	0.7307	0.5772	0.6227	0.6316	0.6168	0.6502	0.5890	0.6263
TS9	0.5611	0.5855	0.9042	1.1170	0.8362	0.9351	0.9603	0.8247	1.0219	0.7658	0.9448
TS10	0.5785	0.5534	0.6326	0.9746	0.8048	0.6267	0.6331	0.7193	1.1550	0.5768	0.6282

**Table A13:** Climate time series: Polya model (ρ=5%).

Time	DLM${}_{1}$+BIC	DLM${}_{1}$+EBIC	CDRec	GROUSE	ROSL	SoftImp	SVDImp	SVT	TeNMF	DynaMMo	TRMF
Series	$n_{u}$ = 240	$n_{u}$ = 240									
	s = 2	s = 2									
	$n_{3}$ = 60	$n_{3}$ = 180									
TS1	0.6019	0.6286	1.0781	0.8916	1.0337	0.9956	1.0334	0.7390	0.9045	0.7050	1.0084
TS2	0.6786	0.6940	0.5419	0.8247	0.7931	0.5352	0.5483	0.6119	0.6680	0.4838	0.5357
TS3	0.8349	0.8430	0.9059	0.9763	0.8577	0.8413	0.8516	0.7393	0.8804	0.7465	0.8461
TS4	0.7421	0.7546	0.9245	1.5698	1.0730	0.9482	0.9619	0.8343	1.0510	0.8339	0.9562
TS5	0.8035	0.7982	0.4500	0.6195	0.5107	0.4418	0.4440	0.5061	0.4560	0.5069	0.4429
TS6	0.7918	0.7928	0.5804	0.9459	0.5751	0.5601	0.5641	0.6265	0.5883	0.5566	0.5611
TS7	0.6343	0.6429	0.6050	0.8494	0.7045	0.6001	0.6081	0.6968	1.0063	0.4972	0.6016
TS8	0.8325	0.8272	0.4869	0.6452	0.4359	0.5142	0.5324	0.4633	0.5274	0.4649	0.5260
TS9	0.7796	0.7597	0.9050	1.0376	0.8629	0.9409	0.9614	0.7744	1.0603	0.8168	0.9501
TS10	0.7898	0.8020	0.5199	0.7190	0.7784	0.5365	0.5333	0.7095	0.9513	0.5317	0.5329

**Table A14:** Climate time series: Polya model (ρ=10%).

Time	DLM${}_{1}$+BIC	DLM${}_{1}$+EBIC	CDRec	GROUSE	ROSL	SoftImp	SVDImp	SVT	TeNMF	DynaMMo	TRMF
Series	$n_{u}$ = 240	$n_{u}$ = 180									
	s = 2	s = 2									
	$n_{3}$ = 60	$n_{3}$ = 120									
TS1	0.6526	0.6667	0.8860	0.7867	0.8675	0.8564	0.8729	0.7541	0.8396	0.6533	0.8603
TS2	0.7942	0.7860	0.6225	0.8560	0.8206	0.5960	0.6150	0.5911	0.7180	0.5224	0.6037
TS3	0.7963	0.8049	0.9341	0.9576	0.8666	0.8795	0.8919	0.7843	0.8915	0.7389	0.8856
TS4	0.7353	0.7373	0.9788	1.3297	1.0548	0.9958	1.0131	0.8311	1.0491	0.8677	1.0024
TS5	0.8248	0.8448	0.5109	0.6593	0.5302	0.5023	0.5063	0.5622	0.5096	0.5459	0.5027
TS6	0.7517	0.7668	0.6397	0.8052	0.5986	0.6210	0.6270	0.6582	0.6115	0.5551	0.6222
TS7	0.6750	0.6689	0.6613	0.7468	0.7378	0.6317	0.6415	0.7031	0.9658	0.4918	0.6358
TS8	0.8206	0.8213	0.6168	0.7687	0.5870	0.6220	0.6312	0.6109	0.6585	0.6154	0.6268
TS9	0.7840	0.7774	0.8855	0.8903	0.8388	0.8976	0.9132	0.8116	1.0499	0.8027	0.9048
TS10	0.7720	0.7626	0.6588	0.8860	0.7420	0.6468	0.6542	0.6921	1.0963	0.6030	0.6491

**Table A15:** Climate time series: Polya model (ρ=15%).

Time	DLM${}_{1}$+BIC	DLM${}_{1}$+EBIC	CDRec	GROUSE	ROSL	SoftImp	SVDImp	SVT	TeNMF	DynaMMo	TRMF
Series	$n_{u}$ = 240	$n_{u}$ = 120									
	s = 2	s = 2									
	$n_{3}$ = 120	$n_{3}$ = 60									
TS1	0.7120	0.6969	0.9835	0.9528	1.0151	0.8908	0.9284	0.7858	0.9998	0.6792	0.9244
TS2	0.7840	0.7775	0.7319	0.9668	0.7852	0.6510	0.6813	0.6460	0.7557	0.5577	0.6814
TS3	0.8321	0.8318	0.9970	0.9484	0.8474	0.8827	0.9195	0.7970	0.8773	0.7077	0.9008
TS4	0.7928	0.7898	0.9036	1.5573	1.0580	0.8831	0.8957	0.8417	1.0299	0.7704	0.8934
TS5	0.8550	0.8477	0.5946	0.7583	0.5791	0.5642	0.5727	0.5928	0.5768	0.6134	0.5725
TS6	0.8186	0.8129	0.6568	0.8705	0.6329	0.6323	0.6415	0.7022	0.6994	0.5884	0.6376
TS7	0.7232	0.7261	0.7734	0.8449	0.7704	0.7267	0.7591	0.7190	1.1403	0.5531	0.7433
TS8	0.8704	0.8561	0.6497	0.8261	0.6203	0.6439	0.6582	0.6273	0.6971	0.6318	0.6553
TS9	0.7777	0.7823	0.9076	0.9681	0.8712	0.9332	0.9592	0.8160	1.0350	0.8200	0.9459
TS10	0.8141	0.8074	0.8027	1.1525	0.8468	0.7601	0.7805	0.7270	1.3561	0.7233	0.7741

**Table A16:** Climate time series: Polya model (ρ=20%).

Time	DLM${}_{1}$+BIC	DLM${}_{1}$+EBIC	CDRec	GROUSE	ROSL	SoftImp	SVDImp	SVT	TeNMF	DynaMMo	TRMF
Series	$n_{u}$ = 240	$n_{u}$ = 120									
	s = 2	s = 2									
	$n_{3}$ = 120	$n_{3}$ = 60									
TS1	0.7124	0.7168	0.8774	1.2102	1.1576	0.8115	0.8445	0.7937	0.9242	0.6061	0.8375
TS2	0.7834	0.7955	0.6768	1.0494	0.7561	0.6077	0.6318	0.6508	0.7583	0.5394	0.6310
TS3	0.8430	0.8503	0.9431	2.1351	0.8514	0.8745	0.8913	0.8084	0.8948	0.7453	0.8830
TS4	0.8271	0.8349	0.9937	1.1897	1.0652	0.9756	0.9902	0.8613	1.0680	0.8587	0.9892
TS5	0.8781	0.8797	0.5866	0.6817	0.6061	0.5705	0.5805	0.6101	0.6239	0.6047	0.5762
TS6	0.8210	0.8231	0.6394	0.9726	0.5960	0.6225	0.6286	0.7040	0.6970	0.5580	0.6232
TS7	0.7946	0.7833	0.6931	0.7721	0.7533	0.6589	0.6744	0.7438	0.9763	0.5410	0.6670
TS8	0.8855	0.8876	0.6334	0.7865	0.6014	0.6361	0.6523	0.6141	0.6693	0.6305	0.6455
TS9	0.8000	0.8022	0.9765	1.0490	0.8575	1.0251	1.0684	0.8287	1.0979	0.8470	1.0535
TS10	0.8267	0.8275	0.6779	0.7324	0.7951	0.6794	0.6842	0.7609	1.1394	0.6444	0.6807

### Appendix A.2. Meteoswiss Time Series: Numerical Results

#### Appendix A.2.1. Results obtained by using DLM

**Table A17:** MeteoSwiss time series: Sampling without replacement (ρ=5%). Comparison of the results obtained for three different values of *m*.

Time	DLM+BIC	DLM+EBIC	DLM+BIC	DLM+EBIC	DLM+BIC	DLM+EBIC
Series	m = 24	m = 24	m = 36	m = 36	m = 48	m = 48
	$n_{u}$ = 480	$n_{u}$ = 480	$n_{u}$ = 720	$n_{u}$ = 540	$n_{u}$ = 960	$n_{u}$ = 720
	s = 3	s = 3	s = 3	s = 3	s = 3	s = 3
	$n_{3}$ = 360	$n_{3}$ = 360	$n_{3}$ = 540	$n_{3}$ = 360	$n_{3}$ = 720	$n_{3}$ = 480
TS1	0.0131	0.0131	0.0127	0.0127	0.0132	0.0132
TS2	0.0072	0.0072	0.0074	0.0075	0.0077	0.0079
TS3	0.0221	0.0221	0.0217	0.0214	0.0226	0.0224
TS4	0.0168	0.0168	0.0158	0.0156	0.0162	0.0166
TS5	0.0175	0.0175	0.0167	0.0170	0.0178	0.0179
TS6	0.0307	0.0307	0.0294	0.0278	0.0276	0.0291
TS7	0.0837	0.0837	0.0836	0.0836	0.0831	0.0834
TS8	0.0796	0.0796	0.0791	0.0796	0.0798	0.0809
TS9	0.0811	0.0811	0.0791	0.0779	0.0811	0.0811
TS10	0.0892	0.0892	0.0866	0.0855	0.0853	0.0849

**Table A18:** MeteoSwiss time series: Sampling without replacement (ρ=5%).

Time	DLM+BIC	DLM+EBIC	CDRec	GROUSE	ROSL	SoftImp	SVDImp	SVT	TeNMF	DynaMMo	TRMF
Series	$n_{u}$ = 480	$n_{u}$ = 480									
	s = 3	s = 3									
	$n_{3}$ = 360	$n_{3}$ = 360									
TS1	0.0131	0.0131	0.0632	0.0842	0.0568	0.0600	0.0600	0.0543	0.0589	0.0538	0.0589
TS2	0.0072	0.0072	0.0443	0.0847	0.0428	0.0429	0.0430	0.0419	0.0642	0.0407	0.0426
TS3	0.0221	0.0221	0.0932	0.1155	0.0734	0.0896	0.0910	0.0849	0.0864	0.0718	0.0873
TS4	0.0168	0.0168	0.1001	0.2101	0.1044	0.0999	0.1002	0.1003	0.0960	0.0809	0.0975
TS5	0.0175	0.0175	0.0747	0.1572	0.0626	0.0714	0.0718	0.0629	0.0654	0.0588	0.0695
TS6	0.0307	0.0307	0.0898	0.1270	0.0900	0.0892	0.0899	0.0846	0.0933	0.0841	0.0883
TS7	0.0837	0.0837	0.2110	0.1861	0.1327	0.1577	0.1604	0.1498	0.1531	0.1302	0.1511
TS8	0.0796	0.0796	0.2266	0.2984	0.1465	0.1967	0.2052	0.1827	0.1841	0.1360	0.1809
TS9	0.0811	0.0811	0.1555	0.2963	0.1335	0.1546	0.1565	0.1561	0.1516	0.1302	0.1514
TS10	0.0892	0.0892	0.4508	0.5071	0.1088	0.3451	0.6489	0.1778	0.5598	0.0914	0.2171

**Table A19:** MeteoSwiss time series: Sampling without replacement (ρ=10%).

Time	DLM+BIC	DLM+EBIC	CDRec	GROUSE	ROSL	SoftImp	SVDImp	SVT	TeNMF	DynaMMo	TRMF
Series	$n_{u}$ = 480	$n_{u}$ = 360									
	s = 3	s = 3									
	$n_{3}$ = 360	$n_{3}$ = 240									
TS1	0.0137	0.0133	0.0578	0.1057	0.0535	0.0570	0.0569	0.0524	0.0678	0.0508	0.0564
TS2	0.0068	0.0069	0.0432	0.1585	0.0417	0.0424	0.0424	0.0415	0.0693	0.0397	0.0421
TS3	0.0212	0.0213	0.1035	0.1145	0.0822	0.1006	0.1038	0.0903	1.0991	0.0797	0.0975
TS4	0.0161	0.0159	0.0980	0.2848	0.0981	0.0990	0.1002	0.0924	0.0972	0.0794	0.0965
TS5	0.0249	0.0254	0.0835	0.1637	0.0677	0.0808	0.0818	0.0713	0.0816	0.0665	0.0787
TS6	0.0217	0.0210	0.0817	0.1328	0.0854	0.0822	0.0826	0.0795	0.0923	0.0771	0.0814
TS7	0.0698	0.0692	0.2144	0.2144	0.1248	0.1677	0.1743	0.1695	0.1819	0.1304	0.1588
TS8	0.0741	0.0757	0.2603	0.3124	0.1413	0.2138	0.2305	0.1814	0.2184	0.1299	0.1897
TS9	0.0756	0.0770	0.1617	0.3264	0.1327	0.1600	0.1631	0.1652	0.1739	0.1345	0.1555
TS10	0.0818	0.0825	0.4701	0.5596	0.1168	0.3541	0.6148	0.2073	0.2140	0.0883	0.2412

**Table A20:** MeteoSwiss time series: Sampling without replacement (ρ=15%).

Time	DLM+BIC	DLM+EBIC	CDRec	GROUSE	ROSL	SoftImp	SVDImp	SVT	TeNMF	DynaMMo	TRMF
Series	$n_{u}$ = 480	$n_{u}$ = 360									
	s = 3	s = 3									
	$n_{3}$ = 360	$n_{3}$ = 240									
TS1	0.0140	0.0141	0.0600	0.1126	0.0543	0.0595	0.0600	0.0526	0.0706	0.0504	0.0580
TS2	0.0079	0.0079	0.0440	0.1901	0.0418	0.0437	0.0439	0.0420	0.0741	0.0412	0.0433
TS3	0.0273	0.0278	0.1059	0.1144	0.0775	0.1010	0.1041	0.0904	1.3698	0.0781	0.0973
TS4	0.0178	0.0181	0.0968	0.3018	0.0922	0.0962	0.0972	0.0888	0.0988	0.0798	0.0945
TS5	0.0202	0.0197	0.0789	0.1820	0.0639	0.0771	0.0781	0.0658	0.0808	0.0616	0.0745
TS6	0.0236	0.0237	0.0807	0.1393	0.0838	0.0798	0.0804	0.0767	0.0862	0.0726	0.0785
TS7	0.0821	0.0814	0.2798	0.2311	0.1378	0.2181	0.2335	0.1828	0.1938	0.1432	0.1915
TS8	0.0698	0.0703	0.2473	0.3242	0.1295	0.2150	0.2495	0.1707	0.2061	0.1181	0.1802
TS9	0.0763	0.0766	0.1599	0.3366	0.1235	0.1543	0.1609	0.1520	0.1693	0.1247	0.1451
TS10	0.0801	0.0808	0.4100	0.5457	0.1105	0.3144	0.4666	0.1997	0.2029	0.0905	0.2465

**Table A21:** MeteoSwiss time series: Sampling without replacement (ρ=20%).

Time	DLM+BIC	DLM+EBIC	CDRec	GROUSE	ROSL	SoftImp	SVDImp	SVT	TeNMF	DynaMMo	TRMF
Series	$n_{u}$ = 480	$n_{u}$ = 240									
	s = 3	s = 3									
	$n_{3}$ = 360	$n_{3}$ = 120									
TS1	0.0150	0.0148	0.0599	0.1525	0.0550	0.0586	0.0587	0.0530	0.0583	0.0501	0.0581
TS2	0.0082	0.0078	0.0473	0.1513	0.0447	0.0467	0.0468	0.0448	0.0634	0.0432	0.0464
TS3	0.0255	0.0254	0.0978	0.1047	0.0675	0.0939	0.0981	0.0824	0.0872	0.0722	0.0899
TS4	0.0185	0.0178	0.1071	0.2660	0.1038	0.1078	0.1090	0.0983	0.0998	0.0823	0.1060
TS5	0.0262	0.0264	0.0805	0.2220	0.0677	0.0778	0.0787	0.0686	0.0723	0.0629	0.0763
TS6	0.0262	0.0258	0.0838	0.1916	0.0817	0.0821	0.0831	0.0778	0.0882	0.0761	0.0808
TS7	0.0857	0.0837	0.2612	0.2160	0.1322	0.1914	0.1965	0.1710	0.2318	0.1366	0.1801
TS8	0.0753	0.0749	0.2758	0.3150	0.1409	0.2204	0.2543	0.1844	0.2695	0.1334	0.1846
TS9	0.0751	0.0747	0.1670	0.3036	0.1297	0.1576	0.1640	0.1581	0.2140	0.1311	0.1530
TS10	0.0849	0.0841	0.4239	0.6182	0.1152	0.3196	0.5181	0.1989	0.4389	0.0916	0.2393

**Table A22:** MeteoSwiss time series: Polya model (ρ=5%).

Time	DLM+BIC	DLM+EBIC	CDRec	GROUSE	ROSL	SoftImp	SVDImp	SVT	TeNMF	DynaMMo	TRMF
Series	$n_{u}$ = 480	$n_{u}$ = 480									
	s = 3	s = 3									
	$n_{3}$ = 360	$n_{3}$ = 360									
TS1	0.0662	0.0662	0.0596	0.0761	0.0504	0.0581	0.0588	0.0468	0.0531	0.0512	0.0572
TS2	0.0339	0.0339	0.0342	0.0698	0.0340	0.0336	0.0338	0.0334	0.0579	0.0330	0.0335
TS3	0.0343	0.0343	0.0769	0.0816	0.0806	0.0774	0.0803	0.0640	0.0696	0.0669	0.0755
TS4	0.0475	0.0475	0.1082	0.2101	0.1103	0.1070	0.1082	0.1042	0.1037	0.0951	0.1056
TS5	0.0503	0.0503	0.0760	0.1304	0.0722	0.0758	0.0769	0.0620	0.0706	0.0719	0.0753
TS6	0.0287	0.0287	0.0526	0.0977	0.0471	0.0514	0.0520	0.0463	0.0592	0.0485	0.0511
TS7	0.1435	0.1435	0.2788	0.2602	0.1652	0.2182	0.2208	0.2027	0.1809	0.1751	0.2101
TS8	0.1302	0.1302	0.1816	0.2066	0.1001	0.1637	0.1776	0.1199	0.1436	0.1175	0.1511
TS9	0.1119	0.1119	0.1612	0.2597	0.1435	0.1630	0.1645	0.1589	0.1652	0.1483	0.1611
TS10	0.1388	0.1388	0.3856	0.5716	0.1954	0.3284	0.5085	0.2159	0.5496	0.1697	0.2791

**Table A23:** MeteoSwiss time series: Polya model (ρ=10%).

Time	DLM+BIC	DLM+EBIC	CDRec	GROUSE	ROSL	SoftImp	SVDImp	SVT	TeNMF	DynaMMo	TRMF
Series	$n_{u}$ = 480	$n_{u}$ = 360									
	s = 3	s = 3									
	$n_{3}$ = 360	$n_{3}$ = 240									
TS1	0.0494	0.0469	0.0687	0.0967	0.0574	0.0640	0.0660	0.0602	0.0906	0.0595	0.0643
TS2	0.0106	0.0098	0.0363	0.0818	0.0322	0.0353	0.0357	0.0346	0.0761	0.0340	0.0356
TS3	0.0414	0.0436	0.0819	0.0841	0.0687	0.0744	0.0843	0.0664	1.1017	0.0653	0.0758
TS4	0.0371	0.0408	0.1135	0.2003	0.1137	0.1085	0.1118	0.1028	0.1164	0.0951	0.1088
TS5	0.0503	0.0535	0.0890	0.1923	0.0735	0.0860	0.0847	0.0763	0.1058	0.0750	0.0835
TS6	0.0250	0.0252	0.0679	0.1027	0.0598	0.0628	0.0673	0.0590	0.0888	0.0601	0.0650
TS7	0.1053	0.1848	0.2267	0.1806	0.1294	0.1621	0.1757	0.1439	0.1461	0.1402	0.1632
TS8	0.0999	0.0976	0.2089	0.2503	0.1340	0.1845	0.2061	0.1577	0.1627	0.1316	0.1765
TS9	0.0948	0.0970	0.1397	0.2586	0.1147	0.1560	0.1350	0.1384	0.1400	0.1165	0.1303
TS10	0.1283	0.1371	0.4732	0.4846	0.2142	0.3564	0.6150	0.1764	0.1781	0.1611	0.3046

**Table A24:** MeteoSwiss time series: model (ρ=15%).

Time	DLM+BIC	DLM+EBIC	CDRec	GROUSE	ROSL	SoftImp	SVDImp	SVT	TeNMF	DynaMMo	TRMF
Series	$n_{u}$ = 480	$n_{u}$ = 240									
	s = 3	s = 3									
	$n_{3}$ = 360	$n_{3}$ = 120									
TS1	0.0636	0.1029	0.0654	0.1085	0.0591	0.0645	0.0645	0.0557	0.0621	0.0558	0.0622
TS2	0.0206	0.0268	0.0512	0.1120	0.0501	0.0510	0.0510	0.0504	0.0605	0.0477	0.0503
TS3	0.0562	0.0689	0.0971	0.1097	0.0750	0.0954	0.0986	0.0830	0.0870	0.0786	0.0915
TS4	0.0445	0.0497	0.1057	0.1876	0.0955	0.1147	0.1120	0.0909	0.0966	0.0915	0.1108
TS5	0.0579	0.0662	0.0846	0.1901	0.0765	0.0846	0.0838	0.0787	0.0759	0.0708	0.0813
TS6	0.0560	0.0528	0.0903	0.1275	0.0966	0.0890	0.0888	0.0881	0.0908	0.0848	0.0880
TS7	0.1439	0.1460	0.2224	0.2122	0.1387	0.2002	0.1948	0.1660	0.1811	0.1337	0.1670
TS8	0.1821	0.1628	0.3414	0.3165	0.1844	0.2314	0.2947	0.2059	0.2376	0.1684	0.2199
TS9	0.1176	0.1424	0.1514	0.2577	0.1339	0.1521	0.1517	0.1515	0.1647	0.1232	0.1389
TS10	0.1184	0.1976	0.4239	0.5578	0.1603	0.3077	0.4959	0.1865	0.4601	0.1557	0.2561

**Table A25:** MeteoSwiss time series: Polya model (ρ=20%).

Time	DLM+BIC	DLM+EBIC	CDRec	GROUSE	ROSL	SoftImp	SVDImp	SVT	TeNMF	DynaMMo	TRMF
Series	$n_{u}$ = 480	$n_{u}$ = 240									
	s = 3	s = 3									
	$n_{3}$ = 360	$n_{3}$ = 120									
TS1	0.4647	0.1228	0.0649	0.2441	0.0579	0.0668	0.0660	0.0574	0.0635	0.0563	0.0653
TS2	0.3674	0.0354	0.0421	0.1161	0.0392	0.0429	0.0422	0.0397	0.0559	0.0394	0.0425
TS3	0.1817	0.0649	0.1071	0.1151	0.0886	0.1033	0.1051	0.0912	0.0915	0.0826	0.1002
TS4	2.2741	0.2579	0.1095	0.3647	0.1123	0.1123	0.1134	0.1075	0.1065	0.1007	0.1111
TS5	0.2996	0.0372	0.0707	0.2183	0.0651	0.0712	0.0720	0.0618	0.0641	0.0588	0.0692
TS6	0.0497	0.0541	0.0802	0.2665	0.0809	0.0789	0.0798	0.0747	0.0875	0.0756	0.0789
TS7	0.1491	0.1553	0.3252	0.2187	0.1312	0.2684	0.2354	0.1697	0.2019	0.1418	0.2120
TS8	0.1292	0.1283	0.3217	0.2758	0.1482	0.2013	0.2085	0.1614	0.2303	0.1446	0.1768
TS9	0.1575	0.1632	0.2079	0.2847	0.1510	0.2002	0.2070	0.1704	0.1972	0.1474	0.1772
TS10	0.1196	0.1184	0.3875	0.5614	0.1741	0.2957	0.5101	0.1741	0.5137	0.1365	0.2499

#### Appendix A.2.2. Results Obtained by Using DLM_1_

**Table A26:** MeteoSwiss time series: Sampling without replacement (ρ=5%).

Time	DLM${}_{1}$+BIC	DLM${}_{1}$+EBIC	CDRec	GROUSE	ROSL	SoftImp	SVDImp	SVT	TeNMF	DynaMMo	TRMF
Series	$n_{u}$ = 480	$n_{u}$ = 480									
	s = 3	s = 3									
	$n_{3}$ = 360	$n_{3}$ = 360									
TS1	0.0089	0.0089	0.0443	0.0620	0.0356	0.0426	0.0429	0.0338	0.0410	0.0365	0.0416
TS2	0.0059	0.0059	0.0290	0.0635	0.0256	0.0270	0.0272	0.0249	0.0424	0.0251	0.0266
TS3	0.0142	0.0142	0.0703	0.0697	0.0446	0.0661	0.0674	0.0554	0.0593	0.0508	0.0640
TS4	0.0099	0.0099	0.0641	0.1469	0.0573	0.0661	0.0669	0.0572	0.0627	0.0533	0.0644
TS5	0.0117	0.0117	0.0552	0.1079	0.0402	0.0526	0.0531	0.0410	0.0461	0.0415	0.0509
TS6	0.0131	0.0131	0.0588	0.0956	0.0502	0.0583	0.0588	0.0517	0.0635	0.0537	0.0576
TS7	0.0683	0.0683	0.1743	0.1398	0.1045	0.1310	0.1336	0.1160	0.1261	0.1081	0.1253
TS8	0.0688	0.0688	0.1847	0.2337	0.1099	0.1609	0.1693	0.1388	0.1475	0.1104	0.1472
TS9	0.0688	0.0688	0.1272	0.2200	0.1075	0.1290	0.1312	0.1196	0.1246	0.1078	0.1263
TS10	0.0713	0.0713	0.3877	0.4032	0.0906	0.2934	0.5471	0.1396	0.4733	0.0768	0.1862

**Table A27:** MeteoSwiss time series: Sampling without replacement (ρ=10%).

Time	DLM${}_{1}$+BIC	DLM${}_{1}$+EBIC	CDRec	GROUSE	ROSL	SoftImp	SVDImp	SVT	TeNMF	DynaMMo	TRMF
Series	$n_{u}$ = 480	$n_{u}$ = 240									
	s = 3	s = 3									
	$n_{3}$ = 360	$n_{3}$ = 120									
TS1	0.0093	0.0091	0.0409	0.0709	0.0334	0.0404	0.0406	0.0325	0.0503	0.0351	0.0399
TS2	0.0058	0.0057	0.0291	0.0714	0.0255	0.0277	0.0278	0.0255	0.0498	0.0259	0.0274
TS3	0.0141	0.0135	0.0784	0.0683	0.0473	0.0748	0.0774	0.0578	1.1717	0.0564	0.0720
TS4	0.0112	0.0111	0.0700	0.1509	0.0572	0.0731	0.0740	0.0570	0.0716	0.0575	0.0715
TS5	0.0151	0.0146	0.0608	0.1205	0.0440	0.0598	0.0608	0.0470	0.0597	0.0476	0.0581
TS6	0.0142	0.0143	0.0554	0.0936	0.0480	0.0557	0.0560	0.0488	0.0633	0.0513	0.0552
TS7	0.0620	0.0628	0.1730	0.1525	0.1017	0.1374	0.1422	0.1243	0.1369	0.1074	0.1304
TS8	0.0665	0.0661	0.2201	0.2336	0.1111	0.1805	0.1953	0.1379	0.1628	0.1097	0.1601
TS9	0.0714	0.0701	0.1343	0.2302	0.1070	0.1338	0.1367	0.1275	0.1348	0.1110	0.1297
TS10	0.0696	0.0692	0.3895	0.4286	0.0925	0.2977	0.5114	0.1490	0.1610	0.0737	0.2037

**Table A28:** MeteoSwiss time series: Sampling without replacement (ρ=15%).

Time	DLM${}_{1}$+BIC	DLM${}_{1}$+EBIC	CDRec	GROUSE	ROSL	SoftImp	SVDImp	SVT	TeNMF	DynaMMo	TRMF
Series	$n_{u}$ = 480	$n_{u}$ = 240									
	s = 3	s = 3									
	$n_{3}$ = 360	$n_{3}$ = 120									
TS1	0.0105	0.0100	0.0419	0.0735	0.0338	0.0416	0.0421	0.0325	0.0513	0.0347	0.0406
TS2	0.0068	0.0063	0.0290	0.0777	0.0250	0.0283	0.0285	0.0254	0.0549	0.0265	0.0280
TS3	0.0185	0.0177	0.0788	0.0676	0.0444	0.0751	0.0776	0.0574	1.3254	0.0554	0.0721
TS4	0.0136	0.0130	0.0666	0.1430	0.0514	0.0667	0.0679	0.0533	0.0690	0.0542	0.0656
TS5	0.0161	0.0157	0.0565	0.1186	0.0418	0.0550	0.0561	0.0430	0.0569	0.0430	0.0532
TS6	0.0158	0.0156	0.0557	0.0910	0.0480	0.0548	0.0555	0.0483	0.0641	0.0492	0.0541
TS7	0.0735	0.0726	0.2244	0.1622	0.1096	0.1790	0.1892	0.1317	0.1460	0.1147	0.1577
TS8	0.0684	0.0676	0.2036	0.2241	0.1019	0.1803	0.2069	0.1331	0.1538	0.0990	0.1527
TS9	0.0674	0.0667	0.1295	0.2148	0.0979	0.1257	0.1308	0.1172	0.1297	0.1029	0.1196
TS10	0.0720	0.0707	0.3317	0.3993	0.0859	0.2581	0.3819	0.1412	0.1455	0.0748	0.2023

**Table A29:** MeteoSwiss time series: Sampling without replacement (ρ=20%).

Time	DLM${}_{1}$+BIC	DLM${}_{1}$+EBIC	CDRec	GROUSE	ROSL	SoftImp	SVDImp	SVT	TeNMF	DynaMMo	TRMF
Series	$n_{u}$ = 480	$n_{u}$ = 240									
	s = 3	s = 3									
	$n_{3}$ = 360	$n_{3}$ = 120									
TS1	0.0137	0.0135	0.0422	0.0812	0.0349	0.0417	0.0417	0.0337	0.0416	0.0349	0.0414
TS2	0.0089	0.0089	0.0310	0.0864	0.0272	0.0302	0.0300	0.0275	0.0443	0.0277	0.0301
TS3	0.0217	0.0211	0.0729	0.0631	0.0415	0.0692	0.0710	0.0537	0.0581	0.0515	0.0668
TS4	0.0183	0.0179	0.0709	0.1617	0.0582	0.0735	0.0752	0.0571	0.0652	0.0558	0.0708
TS5	0.0209	0.0202	0.0584	0.1385	0.0427	0.0562	0.0569	0.0441	0.0495	0.0439	0.0547
TS6	0.0192	0.0190	0.0577	0.1064	0.0464	0.0560	0.0570	0.0484	0.0623	0.0513	0.0551
TS7	0.0781	0.0776	0.2131	0.1541	0.1054	0.1593	0.1616	0.1278	0.1697	0.1115	0.1504
TS8	0.0782	0.0789	0.2267	0.2318	0.1072	0.1832	0.2112	0.1390	0.1958	0.1080	0.1516
TS9	0.0744	0.0738	0.1356	0.2153	0.1006	0.1292	0.1349	0.1201	0.1641	0.1067	0.1251
TS10	0.0816	0.0811	0.3491	0.4397	0.0922	0.2656	0.4321	0.1416	0.3671	0.0764	0.1985

**Table A30:** MeteoSwiss time series: Polya model (ρ=5%).

Time	DLM${}_{1}$+BIC	DLM${}_{1}$+EBIC	CDRec	GROUSE	ROSL	SoftImp	SVDImp	SVT	TeNMF	DynaMMo	TRMF
Series	$n_{u}$ = 480	$n_{u}$ = 360									
	s = 3	s = 3									
	$n_{3}$ = 360	$n_{3}$ = 240									
TS1	0.0248	0.0254	0.0437	0.0573	0.0328	0.0418	0.0425	0.0301	0.0381	0.0357	0.0410
TS2	0.0118	0.0115	0.0232	0.0518	0.0202	0.0224	0.0227	0.0207	0.0372	0.0213	0.0222
TS3	0.0293	0.0296	0.0610	0.0507	0.0502	0.0607	0.0636	0.0431	0.0492	0.0517	0.0590
TS4	0.0441	0.0436	0.0713	0.1485	0.0609	0.0720	0.0734	0.0593	0.0683	0.0630	0.0709
TS5	0.0360	0.0366	0.0627	0.1078	0.0514	0.0618	0.0629	0.0466	0.0552	0.0561	0.0610
TS6	0.0191	0.0189	0.0388	0.0709	0.0277	0.0382	0.0389	0.0311	0.0470	0.0370	0.0381
TS7	0.1404	0.1415	0.2331	0.1864	0.1345	0.1853	0.1875	0.1515	0.1535	0.1459	0.1781
TS8	0.0889	0.0893	0.1545	0.1757	0.0889	0.1422	0.1534	0.1044	0.1189	0.1001	0.1318
TS9	0.1064	0.1081	0.1379	0.2048	0.1182	0.1394	0.1405	0.1286	0.1412	0.1251	0.1376
TS10	0.1496	0.1482	0.3241	0.4329	0.1547	0.2839	0.4276	0.1704	0.4547	0.1357	0.2366

**Table A31:** MeteoSwiss time series: Polya model (ρ=10%).

Time	DLM${}_{1}$+BIC	DLM${}_{1}$+EBIC	CDRec	GROUSE	ROSL	SoftImp	SVDImp	SVT	TeNMF	DynaMMo	TRMF
Series	$n_{u}$ = 480	$n_{u}$ = 240									
	s = 3	s = 3									
	$n_{3}$ = 360	$n_{3}$ = 120									
TS1	0.0352	0.0355	0.0515	0.0751	0.0387	0.0470	0.0491	0.0398	0.0743	0.0431	0.0475
TS2	0.0129	0.0131	0.0239	0.0574	0.0190	0.0225	0.0231	0.0207	0.0562	0.0221	0.0229
TS3	0.0331	0.0327	0.0632	0.0525	0.0440	0.0576	0.0649	0.0455	1.1859	0.0502	0.0589
TS4	0.0392	0.0394	0.0745	0.1398	0.0596	0.0766	0.0755	0.0573	0.0852	0.0633	0.0731
TS5	0.0535	0.0534	0.0697	0.1503	0.0530	0.0677	0.0666	0.0547	0.0884	0.0583	0.0650
TS6	0.0205	0.0210	0.0453	0.0733	0.0313	0.0409	0.0450	0.0351	0.0664	0.0389	0.0433
TS7	0.1039	0.1057	0.1896	0.1357	0.1040	0.1248	0.1458	0.1097	0.1140	0.1080	0.1346
TS8	0.1133	0.1132	0.1740	0.1901	0.1035	0.1491	0.1720	0.1163	0.1220	0.1086	0.1473
TS9	0.0923	0.0939	0.1139	0.1891	0.0932	0.1288	0.1119	0.1052	0.1085	0.0960	0.1079
TS10	0.1203	0.1200	0.3914	0.3770	0.1487	0.2934	0.5150	0.1366	0.1433	0.1305	0.2606

**Table A32:** MeteoSwiss time series: Polya model (ρ=15%).

Time	DLM${}_{1}$+BIC	DLM${}_{1}$+EBIC	CDRec	GROUSE	ROSL	SoftImp	SVDImp	SVT	TeNMF	DynaMMo	TRMF
Series	$n_{u}$ = 480	$n_{u}$ = 240									
	s = 3	s = 3									
	$n_{3}$ = 360	$n_{3}$ = 120									
TS1	0.0334	0.0337	0.0476	0.0762	0.0398	0.0463	0.0469	0.0364	0.0468	0.0410	0.0455
TS2	0.0225	0.0229	0.0322	0.0760	0.0286	0.0314	0.0313	0.0294	0.0412	0.0294	0.0310
TS3	0.0459	0.0455	0.0711	0.0660	0.0467	0.0706	0.0722	0.0548	0.0605	0.0583	0.0684
TS4	0.0365	0.0369	0.0701	0.1252	0.0532	0.0750	0.0754	0.0536	0.0634	0.0596	0.0734
TS5	0.0451	0.0445	0.0610	0.1297	0.0490	0.0602	0.0596	0.0507	0.0520	0.0496	0.0582
TS6	0.0381	0.0382	0.0585	0.0920	0.0524	0.0579	0.0577	0.0520	0.0618	0.0537	0.0573
TS7	0.1163	0.1182	0.1831	0.1488	0.1118	0.1670	0.1580	0.1204	0.1414	0.1102	0.1394
TS8	0.1358	0.1380	0.2807	0.2418	0.1337	0.1843	0.2387	0.1462	0.1785	0.1333	0.1777
TS9	0.1099	0.1094	0.1210	0.1935	0.1031	0.1208	0.1206	0.1149	0.1276	0.0985	0.1120
TS10	0.1073	0.1084	0.3307	0.3787	0.1254	0.2411	0.3898	0.1257	0.3848	0.1155	0.1974

**Table A33:** MeteoSwiss time series: Polya model (ρ=20%).

Time	DLM${}_{1}$+BIC	DLM${}_{1}$+EBIC	CDRec	GROUSE	ROSL	SoftImp	SVDImp	SVT	TeNMF	DynaMMo	TRMF
Series	$n_{u}$ = 480	$n_{u}$ = 240									
	s = 3	s = 3									
	$n_{3}$ = 360	$n_{3}$ = 120									
TS1	0.0366	0.0366	0.0479	0.0948	0.0404	0.0491	0.0495	0.0385	0.0474	0.0422	0.0487
TS2	0.0215	0.0217	0.0278	0.0764	0.0248	0.0274	0.0272	0.0254	0.0406	0.0262	0.0272
TS3	0.0526	0.0534	0.0783	0.0704	0.0532	0.0746	0.0770	0.0598	0.0626	0.0614	0.0734
TS4	0.0442	0.0448	0.0697	0.1648	0.0617	0.0764	0.0785	0.0623	0.0697	0.0657	0.0760
TS5	0.0346	0.0352	0.0516	0.1258	0.0421	0.0523	0.0527	0.0413	0.0454	0.0432	0.0510
TS6	0.0343	0.0345	0.0554	0.1068	0.0450	0.0505	0.0521	0.0461	0.0613	0.0515	0.0510
TS7	0.1211	0.1228	0.2628	0.1484	0.1067	0.2076	0.1827	0.1231	0.1556	0.1139	0.1657
TS8	0.1178	0.1189	0.2581	0.2186	0.1195	0.1707	0.1757	0.1264	0.1789	0.1242	0.1522
TS9	0.1229	0.1254	0.1577	0.2102	0.1204	0.1700	0.1755	0.1265	0.1554	0.1184	0.1511
TS10	0.1092	0.1095	0.3014	0.3658	0.1284	0.2354	0.3975	0.1252	0.4010	0.1072	0.1996

### Appendix A.3. Bafu Time Series: Numerical Results

**Table A34:** BAFU time series: Sampling without replacement (ρ=5%).

Time	DLM+BIC	DLM+EBIC	CDRec	GROUSE	ROSL	SoftImp	SVDImp	SVT	TeNMF	DynaMMo	TRMF
Series	$n_{u}$ = 240	$n_{u}$ = 240									
	s = 3	s = 3									
	$n_{3}$ = 180	$n_{3}$ = 180									
TS1	0.1704	0.1704	0.3304	0.3572	0.2872	0.3269	0.3342	0.3709	0.3345	0.2342	0.3344
TS2	0.0825	0.0825	0.5205	0.5891	0.4924	0.4801	0.4912	0.4915	0.4937	0.3400	0.4895
TS3	0.1197	0.1197	0.3776	0.3963	0.3414	0.3725	0.3747	0.3629	0.3761	0.3226	0.3743
TS4	0.0942	0.0942	0.3628	0.6117	0.2387	0.3653	0.3678	0.4574	0.3784	0.2704	0.3731
TS5	0.1469	0.1469	0.4621	0.5363	0.4300	0.4254	0.4427	0.4517	0.4463	0.3087	0.4421
TS6	0.1079	0.1079	0.2530	0.3423	0.2367	0.2460	0.2483	0.2603	0.2508	0.1954	0.2478
TS7	0.1068	0.1068	0.2796	0.3601	0.2328	0.2765	0.2796	0.2886	0.2780	0.2192	0.2791
TS8	0.0730	0.0730	0.6812	0.7431	0.6206	0.7073	0.7489	0.6990	0.7790	0.3866	0.7501
TS9	0.1179	0.1179	0.3826	0.3724	0.2617	0.3607	0.3651	0.3892	0.3650	0.2709	0.3656
TS10	0.0894	0.0894	0.5959	1.9242	0.4447	0.5132	0.5262	0.5334	0.5299	0.3764	0.5273

**Table A35:** BAFU time series: Sampling without replacement (ρ=10%).

Time	DLM+BIC	DLM+EBIC	CDRec	GROUSE	ROSL	SoftImp	SVDImp	SVT	TeNMF	DynaMMo	TRMF
Series	$n_{u}$ = 240	$n_{u}$ = 240									
	s = 3	s = 3									
	$n_{3}$ = 180	$n_{3}$ = 180									
TS1	0.1076	0.1076	0.3339	0.5545	0.2896	0.3254	0.3306	0.3908	0.3388	0.2224	0.3375
TS2	0.0364	0.0364	0.5189	0.6546	0.4649	0.4729	0.4882	0.4953	0.4872	0.3321	0.4880
TS3	0.0378	0.0378	0.4019	0.4255	0.3466	0.3926	0.3964	0.3765	0.3959	0.3400	0.3966
TS4	0.0700	0.0700	0.4214	0.8424	0.2420	0.4147	0.4240	0.4678	0.4425	0.2982	0.4358
TS5	0.1011	0.1011	0.4366	0.6621	0.4093	0.4072	0.4186	0.4575	0.4251	0.2809	0.4208
TS6	0.0327	0.0327	0.2737	0.3570	0.2173	0.2541	0.2598	0.2743	0.2575	0.1986	0.2606
TS7	0.0606	0.0606	0.3218	0.4167	0.2261	0.3063	0.3147	0.3137	0.3049	0.2332	0.3154
TS8	0.0870	0.0870	0.6619	0.6753	0.5922	0.6847	0.7308	0.6860	0.7701	0.3650	0.7368
TS9	0.0549	0.0549	0.4081	0.6734	0.2851	0.3798	0.3870	0.3995	0.3906	0.2882	0.3911
TS10	0.0720	0.0720	0.5944	0.7594	0.4215	0.5188	0.5276	0.5593	0.5409	0.3822	0.5362

**Table A36:** BAFU time series: Sampling without replacement (ρ=15%).

Time	DLM+BIC	DLM+EBIC	CDRec	GROUSE	ROSL	SoftImp	SVDImp	SVT	TeNMF	DynaMMo	TRMF
Series	$n_{u}$ = 240	$n_{u}$ = 180									
	s = 3	s = 3									
	$n_{3}$ = 120	$n_{3}$ = 120									
TS1	0.1329	0.1058	0.3601	0.4473	0.2770	0.3350	0.3476	0.3984	0.3521	0.2172	0.3547
TS2	0.0775	0.0645	0.5329	1.2661	0.4723	0.4744	0.4890	0.5038	0.4945	0.3341	0.4872
TS3	0.1090	0.1054	0.4081	0.8585	0.3472	0.4026	0.4039	0.4063	0.4072	0.3503	0.4034
TS4	0.0945	0.1008	0.4453	2.2711	0.2365	0.4262	0.4393	0.4795	0.4612	0.3035	0.4550
TS5	0.1286	0.1258	0.5029	0.6097	0.4294	0.4402	0.4625	0.4699	0.4702	0.3017	0.4683
TS6	0.0653	0.0571	0.2729	1.4182	0.2143	0.2601	0.2620	0.2919	0.2675	0.2000	0.2616
TS7	0.0810	0.0701	0.2980	0.8205	0.2129	0.2952	0.2977	0.3240	0.2979	0.2251	0.2971
TS8	0.0965	0.0805	0.6511	1.3404	0.5688	0.6831	0.7466	0.6785	0.7907	0.3565	0.7574
TS9	0.1024	0.0854	0.4346	0.4647	0.2851	0.3932	0.4041	0.4216	0.4072	0.2928	0.4090
TS10	0.0810	0.0747	0.6314	2.2495	0.4308	0.5305	0.5470	0.5585	0.5695	0.3798	0.5579

**Table A37:** BAFU time series: Sampling without replacement (ρ=20%). Because the values of the errors computed for **GROUSE** are very large, we prefer to replace them with Inf.

Time	DLM+BIC	DLM+EBIC	CDRec	GROUSE	ROSL	SoftImp	SVDImp	SVT	TeNMF	DynaMMo	TRMF
Series	$n_{u}$ = 180	$n_{u}$ = 180									
	s = 3	s = 3									
	$n_{3}$ = 120	$n_{3}$ = 120									
TS1	0.0848	0.0848	0.3876	Inf	0.2684	0.3513	0.3714	0.4112	0.4034	0.2159	0.3830
TS2	0.0622	0.0622	0.5501	Inf	0.4767	0.4923	0.5119	0.5143	0.5237	0.3430	0.5095
TS3	0.0823	0.0823	0.4079	Inf	0.3397	0.4034	0.4067	0.4083	2.4134	0.3472	0.4060
TS4	0.0930	0.0930	0.4864	Inf	0.2145	0.4360	0.4641	0.4856	0.5733	0.2982	0.4955
TS5	0.1214	0.1214	0.5083	Inf	0.4120	0.4365	0.4635	0.4733	0.5047	0.2838	0.4737
TS6	0.0623	0.0623	0.2780	Inf	0.2165	0.2695	0.2745	0.3042	0.3623	0.2028	0.2735
TS7	0.0646	0.0646	0.3186	Inf	0.2205	0.3092	0.3170	0.3338	0.3933	0.2352	0.3164
TS8	0.0898	0.0898	0.7019	Inf	0.5843	0.7152	0.7961	0.6944	0.8595	0.3701	0.8199
TS9	0.0672	0.0672	0.4495	Inf	0.2745	0.3946	0.4113	0.4309	0.4000	0.2842	0.4207
TS10	0.0676	0.0676	0.6526	Inf	0.4336	0.5297	0.5537	0.5622	0.6377	0.3724	0.5729

**Table A38:** BAFU time series: Polya model (ρ=5%).

Time	DLM+BIC	DLM+EBIC	CDRec	GROUSE	ROSL	SoftImp	SVDImp	SVT	TeNMF	DynaMMo	TRMF
Series	$n_{u}$ = 120	$n_{u}$ = 120									
	s = 3	s = 3									
	$n_{3}$ = 60	$n_{3}$ = 60									
TS1	1.8813	1.8813	0.2725	0.3116	0.3384	0.2649	0.2568	0.3787	0.2577	0.1882	0.2573
TS2	0.5524	0.5524	0.4541	0.6188	0.4407	0.4321	0.4408	0.4644	0.4514	0.3381	0.4395
TS3	1.9094	1.9094	0.3680	0.3704	0.3086	0.3600	0.3589	0.3658	0.3621	0.3227	0.3588
TS4	0.7125	0.7125	0.3886	0.6716	0.2287	0.3939	0.4003	0.4443	0.4057	0.3049	0.4012
TS5	1.7162	1.7162	0.4406	0.4870	0.4358	0.4070	0.4239	0.4451	0.4283	0.3496	0.4241
TS6	0.6264	0.6264	0.2784	0.3086	0.2446	0.2697	0.2758	0.2572	0.2755	0.2463	0.2753
TS7	0.6197	0.6197	0.3021	0.3815	0.2507	0.2946	0.2956	0.3342	0.2965	0.2657	0.2952
TS8	0.3984	0.3984	0.8263	0.8333	0.7637	0.8557	0.8992	0.8056	0.9287	0.6502	0.8988
TS9	1.2789	1.2789	0.4155	0.6329	0.3448	0.3654	0.3937	0.3321	0.3963	0.3308	0.3915
TS10	0.3720	0.3720	0.5951	0.6249	0.4378	0.5456	0.5506	0.5692	0.5538	0.4532	0.5499

**Table A39:** BAFU time series: Polya model (ρ=10%).

Time	DLM+BIC	DLM+EBIC	CDRec	GROUSE	ROSL	SoftImp	SVDImp	SVT	TeNMF	DynaMMo	TRMF
Series	$n_{u}$ = 120	$n_{u}$ = 120									
	s = 3	s = 3									
	$n_{3}$ = 60	$n_{3}$ = 60									
TS1	4.2100	4.2100	0.3018	0.3506	0.3016	0.2970	0.2954	0.3871	0.2988	0.2538	0.3006
TS2	0.4861	0.4861	0.4657	0.5445	0.4192	0.4508	0.4665	0.4758	0.4497	0.3634	0.4647
TS3	4.5739	4.5739	0.4404	0.4795	0.3913	0.4421	0.4436	0.4246	0.4451	0.4093	0.4435
TS4	0.4285	0.4285	0.3580	0.6293	0.2401	0.3632	0.3665	0.4752	0.3752	0.2898	0.3703
TS5	0.4976	0.4976	0.4515	0.5132	0.3945	0.4114	0.4392	0.4224	0.4384	0.3370	0.4417
TS6	4.9249	4.9249	0.2870	0.3442	0.2310	0.2707	0.2739	0.2789	0.2800	0.2448	0.2735
TS7	4.5368	4.5368	0.3370	0.4155	0.2269	0.3368	0.3428	0.3336	0.3398	0.2930	0.3424
TS8	0.5594	0.5594	0.6441	0.7098	0.5825	0.6651	0.7058	0.7008	0.7352	0.4740	0.7090
TS9	4.3944	4.3944	0.3786	0.3910	0.2529	0.3520	0.3577	0.3971	0.3601	0.2972	0.3617
TS10	0.4510	0.4510	0.6388	1.9624	0.4745	0.5218	0.5515	0.5197	0.5564	0.4364	0.5498

**Table A40:** BAFU time series: Polya model (ρ=15%).

Time	DLM+BIC	DLM+EBIC	CDRec	GROUSE	ROSL	SoftImp	SVDImp	SVT	TeNMF	DynaMMo	TRMF
Series	$n_{u}$ = 120	$n_{u}$ = 120									
	s = 3	s = 3									
	$n_{3}$ = 60	$n_{3}$ = 60									
TS1	12.3811	12.3811	0.3236	0.3763	0.3152	0.3376	0.3287	0.4315	0.3247	0.2398	0.3261
TS2	4.1211	4.1211	0.5430	0.5602	0.4583	0.5079	0.5323	0.5080	0.5278	0.4073	0.5299
TS3	13.2898	13.2898	0.3849	0.4221	0.3286	0.3867	0.3863	0.3998	0.3899	0.3580	0.3862
TS4	3.5273	3.5273	0.4835	0.8293	0.2489	0.4534	0.4762	0.4879	0.4877	0.3812	0.4977
TS5	3.7108	3.7108	0.4263	0.5397	0.4142	0.4016	0.4068	0.4686	0.4083	0.3086	0.4053
TS6	13.1381	13.1381	0.2707	0.5157	0.2118	0.2693	0.2764	0.2772	0.2752	0.2313	0.2759
TS7	11.1314	11.1314	0.3065	0.3823	0.2386	0.3057	0.3125	0.3161	0.3099	0.2633	0.3114
TS8	2.9338	2.9338	0.6709	0.8033	0.5345	0.6981	0.8071	0.6487	0.8634	0.4909	0.8371
TS9	12.4301	12.4301	0.4007	0.4940	0.2337	0.3622	0.3684	0.4105	0.3715	0.2962	0.3711
TS10	4.2552	4.2552	0.6245	1.7983	0.3975	0.5131	0.5368	0.5533	0.5498	0.4139	0.5506

**Table A41:** BAFU time series: Polya model (ρ=20%).

Time	DLM+BIC	DLM+EBIC	CDRec	GROUSE	ROSL	SoftImp	SVDImp	SVT	TeNMF	DynaMMo	TRMF
Series	$n_{u}$ = 180	$n_{u}$ = 180									
	s = 3	s = 3									
	$n_{3}$ = 120	$n_{3}$ = 120									
TS1	2.3474	2.3474	0.3607	2.0702	0.2869	0.3333	0.3384	0.4170	0.3543	0.2509	0.3502
TS2	1.5574	1.5574	0.5659	1.5948	0.4837	0.5223	0.5476	0.5241	0.5439	0.4218	0.5455
TS3	1.6992	1.6992	0.3851	0.6471	0.3171	0.3798	0.3812	0.4010	0.3847	0.3442	0.3819
TS4	1.3567	1.3567	0.4024	4.2599	0.2254	0.3877	0.3987	0.4711	0.4118	0.3025	0.4122
TS5	1.7202	1.7202	0.5140	3.7538	0.4206	0.4392	0.4620	0.4774	0.4790	0.3378	0.4749
TS6	1.1294	1.1294	0.2815	3.0014	0.2368	0.2755	0.2773	0.3103	0.2796	0.2338	0.2767
TS7	1.8391	1.8391	0.2799	0.3789	0.2008	0.2799	0.2777	0.3361	0.2775	0.2354	0.2784
TS8	2.0499	2.0499	0.6696	0.7806	0.6095	0.6933	0.7421	0.7131	0.7722	0.4887	0.7541
TS9	2.3553	2.3553	0.4158	0.5742	0.2695	0.3950	0.3994	0.4413	0.4073	0.3210	0.4099
TS10	2.1197	2.1197	0.6110	4.8646	0.4281	0.5220	0.5354	0.5611	0.5433	0.4226	0.5419

### Appendix A.4. Temperature Time Series: Numerical Results

**Table A42:** Temperature time series: The clustering of the time series when the missing data are simulated by sampling without replacement (SWP) or by the Polya model (Polya).

Simulation Method	Percentage Missing Data	Group1	Group2	Group3	Group4	Group5
SWP	ρ=5%	{7, 10, 11, 12, 13, 15, 18, 19, 20, 25}	{1, 14, 16, 17, 21, 22, 23, 24, 26, 27}	{2, 3, 9, 31, 39, 45, 46, 47, 48, 49}	{28, 29, 30, 32, 33, 34, 36, 37, 38, 41}	{4, 5, 6, 8, 35, 40, 42, 43, 44, 50}
SWP	ρ=10%	{7, 9, 11, 12, 13, 15, 18, 19, 20, 25}	{3, 4, 6, 14, 16, 17, 23, 24, 26, 27}	{1, 2, 10, 21, 22, 45, 46, 47, 48, 49}	{29, 30, 32, 33, 34, 35, 36, 37, 38, 41}	{5, 8, 28, 31, 39, 40, 42, 43, 44, 50}
SWP	ρ=15%	{3, 7, 9, 10, 11, 15, 18, 19, 20, 25}	{4, 6, 14, 16, 17, 21, 23, 24, 26, 27}	{1, 2, 12, 22, 43, 45, 46, 47, 48, 49}	{28, 29, 30, 32, 33, 34, 36, 37, 38, 41}	{5, 8, 13, 31, 35, 39, 40, 42, 44, 50}
SWP	ρ=20%	{7, 9, 10, 11, 13, 18, 19, 20, 22, 25}	{3, 4, 6, 14, 16, 17, 23, 24, 26, 27}	{1, 2, 12, 15, 43, 45, 46, 47, 48, 49}	{29, 30, 32, 33, 34, 35, 36, 37, 38, 41}	{5, 8, 21, 28, 31, 39, 40, 42, 44, 50}
Polya	ρ=5%	{3, 7, 9, 11, 12, 13, 18, 19, 22, 25}	{6, 14, 16, 23, 43, 45, 46, 47, 48, 49}	{1, 2, 10, 15, 17, 20, 21, 24, 26, 27}	{29, 30, 32, 33, 34, 35, 36, 37, 38, 41}	{4, 5, 8, 28, 31, 39, 40, 42, 44, 50}
Polya	ρ=10%	{3, 7, 9, 11, 12, 13, 15, 18, 20, 25}	{2, 10, 14, 16, 17, 21, 22, 23, 24, 26}	{1, 6, 19, 27, 39, 45, 46, 47, 48, 49}	{28, 29, 30, 31, 32, 33, 35, 37, 38, 41}	{4, 5, 8, 34, 36, 40, 42, 43, 44, 50}
Polya	ρ=15%	{6, 7, 9, 14, 16, 17, 18, 20, 22, 23}	{1, 2, 3, 10, 11, 12, 13, 15, 19, 25}	{4, 21, 24, 26, 27, 45, 46, 47, 48, 49}	{28, 29, 30, 32, 33, 34, 36, 37, 38, 41}	{5, 8, 31, 35, 39, 40, 42, 43, 44, 50}
Polya	ρ=20%	{3, 6, 7, 9, 10, 15, 16, 19, 24, 26}	{1, 11, 12, 14, 25, 43, 45, 46, 47, 49}	{2, 4, 5, 13, 18, 20, 21, 22, 23, 27}	{28, 29, 30, 31, 32, 34, 35, 36, 37, 41}	{8, 17, 33, 38, 39, 40, 42, 44, 48, 50}

**Table A43:** Temperature time series: Sampling without replacement (ρ=5%).

Time	DLM+BIC	DLM+EBIC	CDRec	GROUSE	ROSL	SoftImp	SVDImp	SVT	TeNMF	DynaMMo	TRMF
Series	$n_{u}$ = 180	$n_{u}$ = 60									
	s = 2	s = 2									
	$n_{3}$ = 120	$n_{3}$ = 60									
TS1	0.1524	0.1552	0.1989	0.2179	0.2102	0.2025	0.2006	0.9385	0.2136	0.1924	0.2003
TS2	0.1329	0.1325	0.1579	0.2326	0.1562	0.1742	0.1731	1.0513	0.1822	0.1627	0.1724
TS3	0.1617	0.1624	0.1355	0.1842	0.1383	0.1505	0.1474	1.0966	0.1627	0.1380	0.1469
TS4	0.1698	0.1689	0.2137	0.2088	0.2154	0.2077	0.2072	0.2554	0.1949	0.2096	0.2073
TS5	0.1618	0.1605	0.2241	0.2315	0.2262	0.2126	0.2125	0.2623	0.1982	0.2174	0.2126
TS6	0.1519	0.1506	0.1865	0.2166	0.1917	0.1699	0.1673	0.2057	0.1605	0.1776	0.1682
TS7	0.1349	0.1375	0.0910	0.1407	0.1003	0.1001	0.0979	1.0619	0.1150	0.0914	0.0972
TS8	0.2055	0.2040	0.2600	0.2668	0.2558	0.2494	0.2488	0.2971	0.2310	0.2553	0.2492
TS9	0.1195	0.1181	0.0917	0.1331	0.0958	0.0987	0.0969	1.1092	0.1067	0.0907	0.0963
TS10	0.1397	0.1422	0.1123	0.1650	0.1113	0.1183	0.1150	1.1267	0.1237	0.1110	0.1148
TS11	0.1307	0.1359	0.0851	0.1573	0.0908	0.1018	0.0981	1.1347	0.1173	0.0884	0.0975
TS12	0.1536	0.1510	0.1030	0.1506	0.1084	0.1174	0.1142	1.0559	0.1361	0.1084	0.1139
TS13	0.1256	0.1299	0.1105	0.1623	0.1137	0.1387	0.1317	1.0398	0.1485	0.1197	0.1315
TS14	0.1404	0.1455	0.1359	0.1684	0.1038	0.1573	0.1564	1.0513	0.1526	0.1534	0.1563
TS15	0.1636	0.1678	0.1194	0.1516	0.1221	0.1213	0.1209	1.1366	0.1309	0.1169	0.1204
TS16	0.1409	0.1424	0.1233	0.1613	0.0865	0.1544	0.1532	1.0450	0.1514	0.1471	0.1531
TS17	0.1840	0.1936	0.1617	0.2006	0.1478	0.1762	0.1755	0.9900	0.1735	0.1721	0.1755
TS18	0.1421	0.1427	0.1010	0.1393	0.1078	0.1152	0.1132	1.1152	0.1320	0.1051	0.1124
TS19	0.1440	0.1361	0.1031	0.1556	0.1139	0.1197	0.1168	1.1451	0.1436	0.1056	0.1159
TS20	0.1757	0.1905	0.1387	0.1874	0.1460	0.1523	0.1472	1.0546	0.1670	0.1428	0.1469
TS21	0.1671	0.1715	0.1919	0.2121	0.1748	0.2145	0.2146	0.9775	0.2147	0.2051	0.2141
TS22	0.1489	0.1538	0.1260	0.1721	0.0923	0.1611	0.1597	1.0943	0.1665	0.1488	0.1592
TS23	0.1553	0.1630	0.1386	0.1640	0.0987	0.1688	0.1692	1.1147	0.1651	0.1643	0.1689
TS24	0.1757	0.1784	0.1478	0.1722	0.1063	0.1781	0.1769	1.0764	0.1707	0.1729	0.1769
TS25	0.1411	0.1450	0.1106	0.1468	0.1133	0.1284	0.1255	1.0740	0.1404	0.1160	0.1248
TS26	0.1799	0.1824	0.1730	0.1914	0.1623	0.1934	0.1918	0.9928	0.1935	0.1859	0.1916
TS27	0.1487	0.1514	0.1755	0.1811	0.1569	0.1908	0.1896	0.9844	0.1881	0.1844	0.1895
TS28	0.1725	0.1805	0.1777	0.2053	0.1793	0.1597	0.1614	0.2268	0.1740	0.1660	0.1610
TS29	0.1302	0.1354	0.1202	0.1771	0.1285	0.1143	0.1135	0.2009	0.1196	0.1162	0.1136
TS30	0.1579	0.1580	0.1872	0.2744	0.1952	0.1773	0.1774	0.2720	0.1843	0.1804	0.1773
TS31	0.1769	0.1816	0.2123	0.2385	0.2314	0.1956	0.1943	0.2707	0.2167	0.2001	0.1943
TS32	0.1473	0.1491	0.1658	0.2583	0.1720	0.1495	0.1473	0.2665	0.1614	0.1530	0.1473
TS33	0.1721	0.1739	0.1663	0.2423	0.1674	0.1620	0.1611	0.2523	0.1597	0.1643	0.1611
TS34	0.1156	0.1155	0.1593	0.2159	0.1638	0.1548	0.1512	0.2355	0.1486	0.1527	0.1515
TS35	0.2026	0.1976	0.2214	0.2618	0.2360	0.2108	0.2060	0.2951	0.2280	0.2137	0.2067
TS36	0.1139	0.1079	0.1358	0.2225	0.1462	0.1296	0.1259	0.2429	0.1280	0.1276	0.1259
TS37	0.1341	0.1305	0.1353	0.2086	0.1415	0.1316	0.1272	0.2402	0.1292	0.1305	0.1277
TS38	0.1289	0.1336	0.1493	0.2241	0.1507	0.1489	0.1451	0.2659	0.1437	0.1431	0.1449
TS39	0.1706	0.1722	0.1951	0.2536	0.2072	0.1893	0.1897	0.2804	0.1953	0.1906	0.1894
TS40	0.1792	0.1775	0.2176	0.2640	0.2348	0.2134	0.2121	0.2998	0.2208	0.2133	0.2121
TS41	0.1511	0.1472	0.1487	0.2354	0.1594	0.1444	0.1411	0.2591	0.1409	0.1430	0.1412
TS42	0.1490	0.1468	0.1717	0.2219	0.1684	0.1719	0.1703	0.2531	0.1659	0.1693	0.1701
TS43	0.1222	0.1177	0.1488	0.2326	0.1445	0.1491	0.1491	0.1823	0.1598	0.1487	0.1489
TS44	0.1522	0.1539	0.2115	0.2405	0.2120	0.2115	0.2116	0.2525	0.2111	0.2111	0.2114
TS45	0.1266	0.1306	0.1771	0.2565	0.2259	0.1346	0.1243	0.9484	0.1554	0.1384	0.1243
TS46	0.0950	0.0964	0.2013	0.2881	0.2473	0.1546	0.1487	0.9922	0.1852	0.1469	0.1477
TS47	0.0937	0.0970	0.2318	0.2668	0.2382	0.2168	0.2105	0.9968	0.1927	0.2209	0.2117
TS48	0.1236	0.1252	0.2821	0.3313	0.2893	0.2608	0.2621	1.0195	0.2385	0.2677	0.2620
TS49	0.0987	0.0996	0.1962	0.2844	0.2462	0.1427	0.1394	0.9890	0.1658	0.1396	0.1377
TS50	0.1010	0.0976	0.1658	0.2344	0.1679	0.1632	0.1632	0.1942	0.1831	0.1641	0.1631

**Table A44:** Temperature time series: Sampling without replacement (ρ=10%).

Time	DLM+BIC	DLM+EBIC	CDRec	GROUSE	ROSL	SoftImp	SVDImp	SVT	TeNMF	DynaMMo	TRMF
Series	$n_{u}$ = 180	$n_{u}$ = 60									
	s = 2	s = 2									
	$n_{3}$ = 180	$n_{3}$ = 60									
TS1	0.1544	0.1572	0.2007	0.2220	0.2048	0.2094	0.2090	0.9293	0.2116	0.1904	0.2083
TS2	0.1516	0.1519	0.1651	0.2186	0.1610	0.1745	0.1744	1.0055	0.1835	0.1617	0.1739
TS3	0.1773	0.1792	0.1807	0.2151	0.1837	0.1829	0.1824	0.9706	0.1851	0.1747	0.1817
TS4	0.1651	0.1628	0.1710	0.2234	0.1701	0.1827	0.1803	0.9035	0.1906	0.1736	0.1794
TS5	0.1460	0.1471	0.2045	0.2043	0.2038	0.1963	0.1967	0.1816	0.2208	0.2039	0.1968
TS6	0.1500	0.1530	0.1357	0.2102	0.1358	0.1480	0.1446	0.9604	0.1508	0.1356	0.1440
TS7	0.1345	0.1367	0.1048	0.1382	0.0978	0.1240	0.1212	1.0473	0.1264	0.1062	0.1211
TS8	0.2213	0.2179	0.2648	0.2731	0.2629	0.2545	0.2541	0.1735	0.2754	0.2663	0.2543
TS9	0.1342	0.1431	0.1072	0.1326	0.1033	0.1197	0.1189	1.0660	0.1226	0.1062	0.1186
TS10	0.1421	0.1406	0.1171	0.1671	0.1091	0.1278	0.1274	1.0677	0.1297	0.1163	0.1271
TS11	0.1580	0.1558	0.1066	0.1616	0.0987	0.1306	0.1295	1.0810	0.1365	0.1124	0.1291
TS12	0.1641	0.1694	0.1269	0.1624	0.1186	0.1546	0.1544	1.0111	0.1620	0.1362	0.1539
TS13	0.1373	0.1401	0.1229	0.1709	0.1143	0.1527	0.1514	1.0133	0.1572	0.1342	0.1509
TS14	0.1505	0.1495	0.1021	0.1681	0.1024	0.1113	0.1066	0.9968	0.1168	0.1085	0.1061
TS15	0.1680	0.1627	0.1285	0.1661	0.1214	0.1503	0.1501	1.0545	0.1553	0.1313	0.1497
TS16	0.1568	0.1581	0.1053	0.1661	0.0978	0.1071	0.1015	0.9768	0.1100	0.1107	0.1017
TS17	0.1653	0.1755	0.1422	0.2016	0.1359	0.1395	0.1369	0.9455	0.1434	0.1377	0.1367
TS18	0.1566	0.1600	0.1297	0.1658	0.1228	0.1570	0.1543	1.0287	0.1606	0.1394	0.1543
TS19	0.1535	0.1550	0.1174	0.1540	0.1109	0.1458	0.1432	1.0264	0.1507	0.1266	0.1430
TS20	0.1648	0.1708	0.1546	0.1935	0.1468	0.1784	0.1790	1.0372	0.1855	0.1637	0.1784
TS21	0.1897	0.1871	0.1915	0.2270	0.1938	0.2031	0.2029	0.9320	0.2064	0.1952	0.2020
TS22	0.1479	0.1506	0.1377	0.1541	0.1344	0.1468	0.1481	1.0559	0.1507	0.1349	0.1476
TS23	0.1646	0.1700	0.1104	0.1568	0.1077	0.1164	0.1105	0.9695	0.1190	0.1195	0.1106
TS24	0.1731	0.1773	0.1242	0.1749	0.1147	0.1214	0.1152	0.9855	0.1258	0.1301	0.1155
TS25	0.1612	0.1633	0.1282	0.1599	0.1210	0.1535	0.1540	1.0474	0.1587	0.1359	0.1534
TS26	0.1844	0.1820	0.1620	0.2073	0.1594	0.1591	0.1565	0.9468	0.1626	0.1537	0.1561
TS27	0.1498	0.1516	0.1523	0.1833	0.1492	0.1534	0.1492	0.9549	0.1536	0.1416	0.1489
TS28	0.1624	0.1692	0.1748	0.2180	0.1865	0.1671	0.1645	0.2565	0.1790	0.1710	0.1647
TS29	0.1289	0.1324	0.1343	0.1830	0.1402	0.1277	0.1268	0.2199	0.1268	0.1325	0.1269
TS30	0.1619	0.1665	0.1768	0.2256	0.1811	0.1703	0.1709	0.2370	0.1702	0.1756	0.1708
TS31	0.1700	0.1771	0.1863	0.2376	0.1993	0.1722	0.1700	0.2779	0.1835	0.1798	0.1700
TS32	0.1650	0.1689	0.1598	0.2692	0.1688	0.1544	0.1509	0.2773	0.1622	0.1577	0.1511
TS33	0.1685	0.1779	0.1771	0.2413	0.1793	0.1747	0.1748	0.2393	0.1709	0.1768	0.1746
TS34	0.1182	0.1237	0.1345	0.1941	0.1362	0.1335	0.1317	0.2112	0.1294	0.1330	0.1316
TS35	0.1741	0.1819	0.1831	0.2366	0.1926	0.1714	0.1695	0.2731	0.1841	0.1778	0.1695
TS36	0.1200	0.1183	0.1346	0.2074	0.1337	0.1315	0.1294	0.2075	0.1234	0.1323	0.1294
TS37	0.1201	0.1173	0.1335	0.1983	0.1316	0.1328	0.1295	0.2220	0.1248	0.1325	0.1297
TS38	0.1456	0.1469	0.1473	0.2075	0.1467	0.1476	0.1456	0.2260	0.1408	0.1467	0.1455
TS39	0.1654	0.1679	0.2035	0.2764	0.2190	0.2010	0.2002	0.2935	0.2034	0.2009	0.1999
TS40	0.1832	0.1886	0.2335	0.2686	0.2317	0.2272	0.2302	0.3098	0.2271	0.2298	0.2293
TS41	0.1456	0.1438	0.1417	0.2273	0.1418	0.1374	0.1370	0.2222	0.1373	0.1397	0.1365
TS42	0.1509	0.1463	0.1647	0.2132	0.1621	0.1641	0.1644	0.2032	0.1611	0.1640	0.1640
TS43	0.1167	0.1143	0.1586	0.2299	0.1563	0.1594	0.1588	0.1537	0.1687	0.1579	0.1586
TS44	0.1686	0.1627	0.1972	0.2239	0.1969	0.1955	0.1959	0.2402	0.1980	0.1962	0.1956
TS45	0.1290	0.1273	0.2319	0.2650	0.2403	0.2438	0.2425	0.9090	0.2482	0.2289	0.2425
TS46	0.0966	0.0941	0.2351	0.2828	0.2393	0.2419	0.2399	0.8823	0.2416	0.2288	0.2401
TS47	0.0987	0.1042	0.2286	0.2765	0.2460	0.1846	0.1746	0.9278	0.1730	0.2144	0.1753
TS48	0.1198	0.1173	0.2628	0.3214	0.2741	0.2138	0.2173	0.9536	0.2114	0.2444	0.2159
TS49	0.0936	0.0937	0.2379	0.2696	0.2596	0.1838	0.1779	0.9676	0.1742	0.2211	0.1784
TS50	0.0980	0.0994	0.1489	0.1955	0.1476	0.1472	0.1471	0.1505	0.1690	0.1481	0.1471

**Table A45:** Temperature time series: Sampling without replacement (ρ=15%).

Time	DLM+BIC	DLM+EBIC	CDRec	GROUSE	ROSL	SoftImp	SVDImp	SVT	TeNMF	DynaMMo	TRMF
Series	$n_{u}$ = 180	$n_{u}$ = 60									
	s = 2	s = 2									
	$n_{3}$ = 120	$n_{3}$ = 60									
TS1	0.1549	0.1573	0.1951	0.2130	0.1942	0.1970	0.1967	0.8871	0.2026	0.1929	0.1962
TS2	0.1582	0.1576	0.1796	0.1974	0.1759	0.1844	0.1825	0.8558	0.1887	0.1794	0.1823
TS3	0.1706	0.1660	0.1515	0.1822	0.1515	0.1571	0.1546	0.9243	0.1611	0.1517	0.1546
TS4	0.1720	0.1722	0.2022	0.2051	0.1890	0.2060	0.2059	0.8923	0.2121	0.2042	0.2057
TS5	0.1443	0.1479	0.2069	0.2136	0.2101	0.2049	0.2053	0.1714	0.2416	0.2054	0.2052
TS6	0.1590	0.1584	0.1606	0.2033	0.1502	0.1657	0.1642	0.8987	0.1696	0.1616	0.1639
TS7	0.1480	0.1507	0.1014	0.1553	0.1044	0.1084	0.1046	0.9741	0.1106	0.1029	0.1046
TS8	0.2093	0.2142	0.2577	0.2689	0.2601	0.2568	0.2569	0.1908	0.2961	0.2573	0.2569
TS9	0.1467	0.1445	0.1010	0.1416	0.1050	0.1050	0.1025	0.9368	0.1077	0.1000	0.1023
TS10	0.1475	0.1525	0.1198	0.1655	0.1218	0.1221	0.1211	0.9696	0.1240	0.1177	0.1206
TS11	0.1594	0.1593	0.0953	0.1498	0.0991	0.1039	0.0987	0.9553	0.1069	0.0986	0.0989
TS12	0.1760	0.1773	0.1136	0.1587	0.1167	0.1226	0.1169	0.8758	0.1242	0.1185	0.1172
TS13	0.1415	0.1407	0.1094	0.1708	0.1043	0.1194	0.1154	0.9429	0.1270	0.1111	0.1148
TS14	0.1579	0.1528	0.1432	0.1509	0.1226	0.1508	0.1496	0.9092	0.1569	0.1477	0.1494
TS15	0.1632	0.1680	0.1027	0.1619	0.1062	0.1091	0.1059	0.9665	0.1133	0.1055	0.1056
TS16	0.1739	0.1700	0.1438	0.1485	0.1284	0.1487	0.1468	0.8829	0.1498	0.1467	0.1469
TS17	0.1944	0.1966	0.1820	0.2172	0.1712	0.1839	0.1839	0.8435	0.1860	0.1832	0.1837
TS18	0.1761	0.1741	0.1136	0.1639	0.1111	0.1229	0.1194	0.9145	0.1312	0.1185	0.1191
TS19	0.1557	0.1521	0.0947	0.1464	0.0937	0.1028	0.1000	0.9539	0.1120	0.0981	0.0993
TS20	0.1690	0.1669	0.1415	0.1805	0.1363	0.1489	0.1468	0.9495	0.1565	0.1430	0.1462
TS21	0.1938	0.1883	0.2194	0.2206	0.2017	0.2214	0.2211	0.8411	0.2238	0.2179	0.2207
TS22	0.1559	0.1524	0.1300	0.1611	0.1310	0.1361	0.1321	0.9180	0.1369	0.1289	0.1320
TS23	0.1653	0.1637	0.1560	0.1562	0.1368	0.1631	0.1601	0.8855	0.1650	0.1584	0.1602
TS24	0.1799	0.1722	0.1607	0.1615	0.1416	0.1665	0.1646	0.8991	0.1695	0.1630	0.1646
TS25	0.1639	0.1606	0.1083	0.1634	0.1052	0.1182	0.1139	0.9391	0.1260	0.1125	0.1135
TS26	0.2007	0.2029	0.1889	0.1910	0.1788	0.1904	0.1892	0.8871	0.1902	0.1888	0.1893
TS27	0.1724	0.1726	0.1856	0.1751	0.1758	0.1876	0.1866	0.8344	0.1886	0.1856	0.1866
TS28	0.1815	0.1809	0.1832	0.2330	0.1987	0.1816	0.1791	0.2813	0.1998	0.1803	0.1789
TS29	0.1435	0.1388	0.1390	0.2040	0.1451	0.1374	0.1338	0.2248	0.1346	0.1362	0.1339
TS30	0.1645	0.1719	0.1795	0.2740	0.1857	0.1767	0.1752	0.2544	0.1756	0.1756	0.1748
TS31	0.1897	0.1886	0.1869	0.2524	0.2066	0.1825	0.1793	0.2849	0.1987	0.1816	0.1793
TS32	0.1556	0.1569	0.1432	0.2580	0.1534	0.1422	0.1377	0.2572	0.1557	0.1420	0.1380
TS33	0.1749	0.1743	0.1800	0.2473	0.1838	0.1785	0.1768	0.2440	0.1740	0.1783	0.1767
TS34	0.1235	0.1240	0.1303	0.1848	0.1273	0.1293	0.1293	0.1947	0.1284	0.1285	0.1289
TS35	0.1827	0.1886	0.1857	0.2577	0.1991	0.1817	0.1812	0.2756	0.1963	0.1825	0.1809
TS36	0.1167	0.1183	0.1276	0.2090	0.1249	0.1288	0.1256	0.1973	0.1239	0.1257	0.1256
TS37	0.1309	0.1319	0.1326	0.2054	0.1276	0.1319	0.1295	0.2093	0.1217	0.1307	0.1295
TS38	0.1439	0.1436	0.1468	0.2191	0.1423	0.1475	0.1450	0.2272	0.1376	0.1446	0.1447
TS39	0.1635	0.1614	0.2032	0.2704	0.2153	0.2013	0.2012	0.2839	0.2080	0.1987	0.2006
TS40	0.2107	0.2068	0.2387	0.2803	0.2475	0.2352	0.2363	0.3035	0.2415	0.2362	0.2358
TS41	0.1564	0.1573	0.1447	0.2293	0.1437	0.1434	0.1427	0.2308	0.1440	0.1419	0.1423
TS42	0.1540	0.1498	0.1699	0.2141	0.1631	0.1704	0.1703	0.1983	0.1710	0.1694	0.1699
TS43	0.1283	0.1297	0.1540	0.3230	0.2087	0.1518	0.1437	0.8887	0.1357	0.1498	0.1442
TS44	0.1650	0.1641	0.1951	0.2278	0.1940	0.1950	0.1950	0.2263	0.1993	0.1945	0.1947
TS45	0.1292	0.1356	0.1438	0.2449	0.2026	0.1400	0.1346	0.8870	0.1242	0.1400	0.1348
TS46	0.0974	0.0997	0.1538	0.2943	0.2082	0.1472	0.1428	0.8133	0.1369	0.1425	0.1426
TS47	0.1167	0.1166	0.1372	0.2480	0.1957	0.1352	0.1284	0.8536	0.1196	0.1301	0.1283
TS48	0.1295	0.1228	0.2876	0.3350	0.2950	0.2877	0.2868	0.7810	0.2871	0.2843	0.2866
TS49	0.1134	0.1082	0.1588	0.2762	0.2107	0.1533	0.1488	0.8078	0.1373	0.1480	0.1485
TS50	0.1084	0.1070	0.1627	0.2334	0.1639	0.1644	0.1633	0.1491	0.1850	0.1637	0.1634

**Table A46:** Temperature time series: Sampling without replacement (ρ=20%).

Time	DLM+BIC	DLM+EBIC	CDRec	GROUSE	ROSL	SoftImp	SVDImp	SVT	TeNMF	DynaMMo	TRMF
Series	$n_{u}$ = 180	$n_{u}$ = 60									
	s = 2	s = 2									
	$n_{3}$ = 120	$n_{3}$ = 60									
TS1	0.1700	0.1661	0.2005	0.2187	0.1981	0.2057	0.2062	0.1779	0.2096	0.1958	0.2054
TS2	0.1676	0.1604	0.1709	0.1940	0.1733	0.1802	0.1788	0.1542	0.1853	0.1699	0.1782
TS3	0.1763	0.1740	0.1520	0.2020	0.1566	0.1615	0.1610	0.1815	0.1700	0.1507	0.1599
TS4	0.1861	0.1770	0.1821	0.2400	0.1801	0.1919	0.1912	0.2067	0.1986	0.1842	0.1902
TS5	0.1589	0.1619	0.2165	0.2248	0.2224	0.2114	0.2112	0.1650	0.2362	0.2179	0.2114
TS6	0.1564	0.1532	0.1309	0.2004	0.1352	0.1453	0.1430	0.1798	0.1528	0.1331	0.1420
TS7	0.1659	0.1566	0.1345	0.1483	0.1153	0.1391	0.1389	0.1239	0.1383	0.1354	0.1387
TS8	0.2192	0.2216	0.2527	0.2593	0.2572	0.2496	0.2500	0.1845	0.2780	0.2542	0.2501
TS9	0.1576	0.1548	0.1269	0.1344	0.1146	0.1333	0.1315	0.1304	0.1330	0.1265	0.1315
TS10	0.1535	0.1456	0.1301	0.1523	0.1191	0.1363	0.1344	0.1212	0.1359	0.1303	0.1344
TS11	0.1610	0.1566	0.1280	0.1472	0.1095	0.1360	0.1350	0.1168	0.1363	0.1297	0.1348
TS12	0.1928	0.1845	0.1518	0.1624	0.1322	0.1619	0.1600	0.1421	0.1630	0.1553	0.1599
TS13	0.1606	0.1545	0.1519	0.1782	0.1218	0.1625	0.1610	0.1214	0.1616	0.1556	0.1608
TS14	0.1732	0.1655	0.1031	0.1514	0.1112	0.1201	0.1154	0.1661	0.1281	0.1101	0.1148
TS15	0.1817	0.1733	0.1387	0.1759	0.1168	0.1460	0.1440	0.1286	0.1443	0.1406	0.1441
TS16	0.1740	0.1683	0.0930	0.1392	0.1025	0.1086	0.1041	0.1720	0.1174	0.1001	0.1036
TS17	0.1903	0.1873	0.1402	0.2201	0.1465	0.1452	0.1433	0.1785	0.1520	0.1411	0.1428
TS18	0.1763	0.1660	0.1585	0.1668	0.1320	0.1691	0.1677	0.1189	0.1694	0.1630	0.1676
TS19	0.1830	0.1771	0.1447	0.1575	0.1179	0.1558	0.1542	0.1223	0.1564	0.1490	0.1540
TS20	0.1880	0.1766	0.1681	0.1784	0.1399	0.1777	0.1757	0.1271	0.1772	0.1719	0.1757
TS21	0.1869	0.1860	0.2151	0.2380	0.2111	0.2219	0.2227	0.2174	0.2266	0.2161	0.2218
TS22	0.1645	0.1574	0.1507	0.1548	0.1400	0.1541	0.1529	0.1751	0.1532	0.1481	0.1527
TS23	0.1776	0.1721	0.1188	0.1668	0.1247	0.1349	0.1290	0.1618	0.1413	0.1261	0.1287
TS24	0.1875	0.1808	0.1241	0.1755	0.1294	0.1352	0.1324	0.1639	0.1439	0.1305	0.1319
TS25	0.1662	0.1621	0.1491	0.1599	0.1185	0.1583	0.1568	0.1140	0.1579	0.1518	0.1566
TS26	0.1973	0.1841	0.1508	0.1775	0.1483	0.1578	0.1552	0.1854	0.1646	0.1483	0.1545
TS27	0.1839	0.1769	0.1565	0.1632	0.1531	0.1643	0.1623	0.1960	0.1696	0.1524	0.1614
TS28	0.1870	0.1896	0.2089	0.2639	0.2274	0.2010	0.1987	0.1715	0.2058	0.2046	0.1987
TS29	0.1387	0.1421	0.1409	0.2083	0.1523	0.1362	0.1351	0.1293	0.1353	0.1382	0.1349
TS30	0.1851	0.1862	0.1948	0.2514	0.2104	0.1931	0.1896	0.1666	0.1918	0.1941	0.1899
TS31	0.1913	0.1885	0.2028	0.2758	0.2234	0.1953	0.1936	0.1604	0.2030	0.1984	0.1935
TS32	0.1964	0.1914	0.1560	0.2801	0.1723	0.1499	0.1474	0.1439	0.1558	0.1524	0.1473
TS33	0.1623	0.1609	0.1715	0.2566	0.1771	0.1705	0.1698	0.1485	0.1696	0.1711	0.1695
TS34	0.1788	0.1789	0.1422	0.1976	0.1459	0.1416	0.1387	0.1126	0.1344	0.1409	0.1389
TS35	0.1329	0.1333	0.1957	0.2825	0.2177	0.1897	0.1841	0.1677	0.1995	0.1913	0.1846
TS36	0.1200	0.1213	0.1252	0.2184	0.1279	0.1243	0.1217	0.1013	0.1198	0.1235	0.1217
TS37	0.1361	0.1345	0.1332	0.2026	0.1368	0.1320	0.1292	0.1065	0.1223	0.1323	0.1294
TS38	0.1468	0.1453	0.1489	0.2150	0.1511	0.1473	0.1461	0.1489	0.1411	0.1468	0.1457
TS39	0.1687	0.1627	0.1976	0.2818	0.2124	0.1931	0.1944	0.1785	0.1941	0.1944	0.1937
TS40	0.2134	0.2126	0.2362	0.2899	0.2496	0.2300	0.2300	0.2041	0.2318	0.2323	0.2296
TS41	0.1573	0.1568	0.1455	0.2572	0.1552	0.1444	0.1418	0.1638	0.1403	0.1435	0.1416
TS42	0.1722	0.1720	0.1703	0.2514	0.1666	0.1718	0.1703	0.1807	0.1695	0.1706	0.1702
TS43	0.1378	0.1293	0.2346	0.2812	0.2243	0.2311	0.2275	0.1760	0.2200	0.2321	0.2284
TS44	0.1670	0.1662	0.2036	0.2486	0.2028	0.2044	0.2034	0.1985	0.2045	0.2036	0.2033
TS45	0.1320	0.1253	0.2351	0.2599	0.2285	0.2256	0.2232	0.1699	0.2123	0.2308	0.2239
TS46	0.1176	0.1105	0.1843	0.2631	0.2265	0.1624	0.1546	0.2436	0.1510	0.1704	0.1554
TS47	0.1208	0.1139	0.1782	0.2492	0.2110	0.1539	0.1492	0.2246	0.1434	0.1641	0.1497
TS48	0.1376	0.1301	0.2217	0.2916	0.2619	0.1923	0.1920	0.2192	0.1958	0.2047	0.1909
TS49	0.1272	0.1120	0.1709	0.2465	0.2197	0.1455	0.1356	0.1684	0.1351	0.1573	0.1362
TS50	0.1077	0.1054	0.1528	0.2274	0.1497	0.1518	0.1517	0.1316	0.1806	0.1517	0.1515

**Table A47:** Temperature time series: Polya model (ρ=5%).

Time	DLM+BIC	DLM+EBIC	CDRec	GROUSE	ROSL	SoftImp	SVDImp	SVT	TeNMF	DynaMMo	TRMF
Series	$n_{u}$ = 180	$n_{u}$ = 60									
	s = 2	s = 2									
	$n_{3}$ = 180	$n_{3}$ = 60									
TS1	0.6251	0.7918	0.1943	0.2233	0.2009	0.2132	0.2064	0.9306	0.2128	0.2009	0.2069
TS2	0.2943	0.3902	0.1540	0.1974	0.1536	0.1791	0.1760	1.0627	0.1830	0.1688	0.1756
TS3	0.4732	0.6096	0.1015	0.1813	0.1093	0.1570	0.1490	0.9682	0.1661	0.1336	0.1490
TS4	0.2650	0.2718	0.2730	0.3063	0.2746	0.2704	0.2707	0.2674	0.2754	0.2727	0.2705
TS5	0.1781	0.1844	0.2208	0.2332	0.2266	0.2056	0.2036	0.2069	0.2127	0.2102	0.2039
TS6	0.5373	0.5793	0.1722	0.1755	0.1532	0.1799	0.1761	0.9048	0.1721	0.1771	0.1767
TS7	0.4028	0.4209	0.1082	0.2025	0.1162	0.1423	0.1329	1.1533	0.1517	0.1235	0.1335
TS8	0.2036	0.2149	0.2099	0.2207	0.2159	0.2021	0.2013	0.2494	0.2069	0.2056	0.2014
TS9	0.4897	0.5682	0.1148	0.1710	0.1202	0.1436	0.1468	1.1226	0.1570	0.1374	0.1458
TS10	0.4729	0.4579	0.1092	0.1250	0.0882	0.1146	0.1162	0.9518	0.1137	0.1176	0.1158
TS11	0.3803	0.3733	0.0943	0.1767	0.0971	0.1243	0.1203	1.0857	0.1425	0.1079	0.1196
TS12	0.2517	0.2599	0.1299	0.1827	0.1375	0.1548	0.1548	0.9963	0.1627	0.1499	0.1543
TS13	0.4682	0.5351	0.1179	0.1914	0.1139	0.1583	0.1491	0.9866	0.1687	0.1337	0.1490
TS14	0.4205	0.5867	0.1307	0.1512	0.1120	0.1583	0.1545	1.0673	0.1549	0.1513	0.1547
TS15	0.5850	0.6819	0.1098	0.1499	0.1096	0.1174	0.1136	1.0315	0.1187	0.1103	0.1134
TS16	0.5879	0.6217	0.1235	0.1366	0.1062	0.1449	0.1441	1.0078	0.1398	0.1451	0.1439
TS17	0.4242	0.5019	0.1849	0.2347	0.1711	0.2067	0.2010	0.9928	0.2015	0.2026	0.2019
TS18	0.4599	0.4919	0.1423	0.1783	0.1514	0.1550	0.1558	1.0880	0.1671	0.1459	0.1546
TS19	0.4677	0.6058	0.1303	0.1676	0.1402	0.1385	0.1413	1.1543	0.1507	0.1384	0.1401
TS20	0.4885	0.5201	0.1885	0.2286	0.1773	0.2110	0.2136	0.9873	0.2137	0.2122	0.2129
TS21	0.3263	0.3047	0.2210	0.2354	0.2065	0.2375	0.2357	0.8911	0.2349	0.2330	0.2357
TS22	0.4590	0.5199	0.1673	0.1987	0.1728	0.1688	0.1717	1.0780	0.1705	0.1696	0.1710
TS23	0.4915	0.4595	0.1676	0.1732	0.1378	0.1743	0.1756	1.0248	0.1674	0.1804	0.1757
TS24	0.4070	0.4336	0.1209	0.1714	0.1067	0.1548	0.1608	1.0263	0.1558	0.1587	0.1596
TS25	0.5323	0.6726	0.1256	0.1731	0.1285	0.1520	0.1465	0.9823	0.1613	0.1362	0.1461
TS26	0.6406	0.6595	0.1639	0.1749	0.1571	0.1850	0.1793	0.9415	0.1841	0.1782	0.1800
TS27	0.3552	0.3690	0.1631	0.1703	0.1523	0.1784	0.1741	0.9150	0.1726	0.1744	0.1746
TS28	0.2693	0.2527	0.2113	0.3084	0.2168	0.1974	0.1972	0.3247	0.2192	0.1974	0.1967
TS29	0.1879	0.1957	0.1746	0.2146	0.1680	0.1542	0.1517	0.2195	0.1557	0.1551	0.1513
TS30	0.2830	0.2954	0.2115	0.2766	0.2143	0.2033	0.1994	0.2651	0.2152	0.2017	0.1992
TS31	0.2338	0.2295	0.1887	0.2634	0.1926	0.1732	0.1738	0.2532	0.1925	0.1752	0.1734
TS32	0.2310	0.2488	0.1960	0.3254	0.1900	0.1760	0.1679	0.3153	0.1981	0.1759	0.1687
TS33	0.2297	0.2203	0.1969	0.2438	0.1959	0.1893	0.1875	0.2707	0.1815	0.1912	0.1875
TS34	0.1747	0.1808	0.1464	0.2108	0.1449	0.1436	0.1407	0.2297	0.1401	0.1384	0.1403
TS35	0.2705	0.2626	0.2214	0.3245	0.2215	0.2026	0.1998	0.2921	0.2242	0.1998	0.1995
TS36	0.2245	0.2209	0.1539	0.2702	0.1509	0.1498	0.1429	0.2400	0.1384	0.1449	0.1431
TS37	0.2109	0.1927	0.1591	0.2398	0.1522	0.1533	0.1491	0.2312	0.1444	0.1516	0.1493
TS38	0.2358	0.2436	0.1802	0.2506	0.1712	0.1813	0.1783	0.2781	0.1705	0.1753	0.1773
TS39	0.2711	0.2144	0.2083	0.3125	0.2163	0.2002	0.1970	0.3206	0.2065	0.1990	0.1969
TS40	0.2585	0.2626	0.2535	0.3093	0.2538	0.2442	0.2422	0.3653	0.2555	0.2413	0.2417
TS41	0.2384	0.2319	0.1626	0.3083	0.1647	0.1576	0.1529	0.2621	0.1627	0.1518	0.1523
TS42	0.2134	0.2191	0.2050	0.2890	0.2039	0.2094	0.2091	0.2479	0.2196	0.2068	0.2081
TS43	0.5072	0.5703	0.1586	0.2081	0.2054	0.1067	0.0991	1.0210	0.1118	0.1086	0.0990
TS44	0.2326	0.2270	0.2357	0.2753	0.2332	0.2359	0.2356	0.2979	0.2400	0.2355	0.2353
TS45	0.4533	0.4960	0.1663	0.2346	0.2107	0.1302	0.1247	0.9224	0.1378	0.1339	0.1252
TS46	0.5780	0.6142	0.2637	0.2916	0.2848	0.2214	0.2052	0.9132	0.2012	0.2216	0.2078
TS47	0.3146	0.2868	0.1754	0.2387	0.2408	0.1238	0.1292	0.9815	0.1555	0.1147	0.1264
TS48	0.3095	0.5182	0.2610	0.2865	0.2519	0.2573	0.2681	0.9740	0.2557	0.2720	0.2670
TS49	0.4215	0.4902	0.2286	0.2973	0.2802	0.1430	0.1569	1.0935	0.1679	0.1707	0.1547
TS50	0.1399	0.1387	0.1713	0.2188	0.1650	0.1724	0.1752	0.1945	0.1975	0.1733	0.1747

**Table A48:** Temperature time series: Polya model (ρ=10%).

Time	DLM+BIC	DLM+EBIC	CDRec	GROUSE	ROSL	SoftImp	SVDImp	SVT	TeNMF	DynaMMo	TRMF
Series	$n_{u}$ = 180	$n_{u}$ = 60									
	s = 2	s = 2									
	$n_{3}$ = 180	$n_{3}$ = 60									
TS1	0.3693	0.3592	0.1844	0.1876	0.1907	0.1811	0.1810	0.8298	0.1801	0.1788	0.1805
TS2	0.4563	0.4058	0.1969	0.2104	0.1990	0.2011	0.2017	0.7576	0.2029	0.1938	0.2011
TS3	0.3496	0.3540	0.1394	0.1744	0.1377	0.1511	0.1484	0.8102	0.1383	0.1382	0.1456
TS4	0.2530	0.2669	0.2639	0.2613	0.2647	0.2595	0.2563	0.1696	0.3009	0.2641	0.2580
TS5	0.2629	0.2548	0.2641	0.2647	0.2633	0.2578	0.2562	0.1836	0.4436	0.2641	0.2576
TS6	0.4133	0.3936	0.1534	0.1656	0.1572	0.1555	0.1507	0.7901	0.1516	0.1481	0.1509
TS7	0.3627	0.3189	0.1075	0.1567	0.1135	0.1312	0.1268	0.8213	0.1085	0.1067	0.1237
TS8	0.3219	0.3445	0.3393	0.3422	0.3367	0.3352	0.3318	0.2194	0.5229	0.3422	0.3341
TS9	0.3771	0.3532	0.1194	0.1661	0.1247	0.1270	0.1236	0.8801	0.1202	0.1125	0.1220
TS10	0.3459	0.3205	0.1102	0.1753	0.1046	0.1420	0.1458	0.8275	0.1350	0.1241	0.1441
TS11	0.3531	0.3791	0.0923	0.1396	0.0946	0.1045	0.1042	0.8625	0.0897	0.0898	0.1003
TS12	0.3414	0.3407	0.1134	0.1704	0.1136	0.1312	0.1319	0.8645	0.1124	0.1198	0.1284
TS13	0.2963	0.2717	0.1183	0.1780	0.1177	0.1308	0.1315	0.8780	0.1141	0.1207	0.1276
TS14	0.3476	0.3094	0.1234	0.1681	0.1060	0.1515	0.1534	0.8265	0.1449	0.1377	0.1531
TS15	0.4053	0.3606	0.1147	0.1770	0.1182	0.1218	0.1160	0.8294	0.1081	0.1095	0.1142
TS16	0.3184	0.3085	0.1285	0.1561	0.1119	0.1537	0.1534	0.8594	0.1485	0.1425	0.1533
TS17	0.3135	0.3322	0.1731	0.2423	0.1648	0.1882	0.1884	0.8014	0.1870	0.1776	0.1881
TS18	0.4253	0.3682	0.1251	0.1788	0.1235	0.1290	0.1288	0.8529	0.1165	0.1245	0.1251
TS19	0.3515	0.3697	0.1126	0.1563	0.1106	0.1113	0.1102	0.8915	0.1022	0.1087	0.1072
TS20	0.3317	0.3151	0.1420	0.1944	0.1413	0.1434	0.1463	0.8751	0.1440	0.1420	0.1426
TS21	0.4300	0.4119	0.2116	0.2274	0.2044	0.2323	0.2307	0.7158	0.2257	0.2206	0.2303
TS22	0.3342	0.3060	0.1189	0.1650	0.1025	0.1612	0.1608	0.8440	0.1505	0.1381	0.1597
TS23	0.3840	0.3771	0.1381	0.1732	0.1219	0.1795	0.1840	0.8045	0.1734	0.1600	0.1818
TS24	0.4084	0.3870	0.1456	0.1624	0.1301	0.1649	0.1646	0.8159	0.1609	0.1595	0.1651
TS25	0.3159	0.2679	0.1177	0.1782	0.1196	0.1330	0.1283	0.8682	0.1125	0.1208	0.1255
TS26	0.4266	0.4381	0.1902	0.1892	0.1813	0.2102	0.2092	0.8313	0.2077	0.1950	0.2095
TS27	0.3137	0.3227	0.1750	0.1700	0.1667	0.1825	0.1830	0.8167	0.1850	0.1770	0.1831
TS28	0.2840	0.2828	0.2173	0.2779	0.2254	0.2033	0.1974	0.3132	0.2272	0.2095	0.1984
TS29	0.2072	0.2065	0.1555	0.2206	0.1580	0.1481	0.1413	0.2582	0.1526	0.1511	0.1428
TS30	0.2408	0.2421	0.1954	0.3166	0.1916	0.1819	0.1780	0.2563	0.1928	0.1886	0.1788
TS31	0.2360	0.2337	0.1905	0.2432	0.1956	0.1783	0.1727	0.3000	0.2060	0.1835	0.1735
TS32	0.2255	0.2179	0.1498	0.2526	0.1566	0.1483	0.1406	0.2679	0.1985	0.1469	0.1417
TS33	0.2501	0.2496	0.1818	0.2439	0.1828	0.1762	0.1718	0.2392	0.1748	0.1778	0.1725
TS34	0.2295	0.2263	0.1785	0.2856	0.1788	0.1771	0.1726	0.2655	0.1745	0.1747	0.1730
TS35	0.2629	0.2563	0.2115	0.2769	0.2116	0.1915	0.1885	0.2948	0.2165	0.2019	0.1892
TS36	0.2345	0.2299	0.1623	0.2781	0.1577	0.1663	0.1604	0.2683	0.1644	0.1615	0.1608
TS37	0.2384	0.2252	0.1781	0.2827	0.1762	0.1790	0.1753	0.2824	0.1712	0.1761	0.1754
TS38	0.2230	0.2282	0.1751	0.2573	0.1720	0.1759	0.1719	0.2601	0.1771	0.1733	0.1721
TS39	0.2875	0.2763	0.2378	0.2927	0.2488	0.2415	0.2431	0.3066	0.2518	0.2384	0.2422
TS40	0.2605	0.2668	0.2279	0.2840	0.2402	0.2258	0.2217	0.3009	0.2319	0.2234	0.2220
TS41	0.2036	0.2020	0.1325	0.2398	0.1344	0.1317	0.1315	0.2337	0.1710	0.1315	0.1312
TS42	0.2152	0.2197	0.1841	0.2566	0.1837	0.1851	0.1831	0.2269	0.2546	0.1829	0.1830
TS43	0.1792	0.1725	0.1742	0.2696	0.1724	0.1739	0.1741	0.1688	0.2284	0.1739	0.1739
TS44	0.2499	0.2329	0.2136	0.2530	0.2190	0.2169	0.2135	0.2688	0.2105	0.2138	0.2138
TS45	0.3143	0.3025	0.2387	0.2776	0.2644	0.1747	0.1562	0.7194	0.2291	0.2207	0.1639
TS46	0.4260	0.4310	0.2310	0.2562	0.2496	0.2168	0.1980	0.7686	0.2559	0.2304	0.2044
TS47	0.2605	0.2345	0.1963	0.2747	0.2183	0.1387	0.1271	0.8120	0.1944	0.1758	0.1314
TS48	0.3240	0.2980	0.2672	0.3463	0.2655	0.2629	0.2712	0.8116	0.3138	0.2607	0.2702
TS49	0.2397	0.2319	0.2503	0.3016	0.2781	0.1679	0.1474	0.6945	0.2561	0.2203	0.1534
TS50	0.1871	0.1799	0.2012	0.2204	0.1988	0.1991	0.2000	0.2055	0.2692	0.1997	0.1997

**Table A49:** Temperature time series: Polya model (ρ=15%).

Time	DLM+BIC	DLM+EBIC	CDRec	GROUSE	ROSL	SoftImp	SVDImp	SVT	TeNMF	DynaMMo	TRMF
Series	$n_{u}$ = 180	$n_{u}$ = 60									
	s = 2	s = 2									
	$n_{3}$ = 180	$n_{3}$ = 60									
TS1	0.4339	0.5828	0.2001	0.2211	0.2028	0.2112	0.2080	0.8866	0.2153	0.1943	0.2068
TS2	0.4861	0.5423	0.1769	0.2345	0.1785	0.1940	0.1929	0.8655	0.2048	0.1795	0.1911
TS3	0.3499	0.4787	0.1473	0.1664	0.1476	0.1621	0.1595	0.8852	0.1611	0.1530	0.1590
TS4	0.3489	0.4905	0.1800	0.2429	0.1887	0.2045	0.2003	0.8673	0.2257	0.1848	0.1965
TS5	0.2042	0.2003	0.2190	0.2175	0.2208	0.2093	0.2094	0.2082	0.2206	0.2184	0.2105
TS6	0.4257	0.5894	0.1495	0.1967	0.1514	0.1586	0.1569	0.8391	0.1735	0.1487	0.1546
TS7	0.4360	0.5145	0.1322	0.1721	0.1352	0.1534	0.1560	0.9712	0.1609	0.1408	0.1540
TS8	0.3043	0.3050	0.2967	0.2975	0.2944	0.2873	0.2884	0.2804	0.3123	0.2949	0.2885
TS9	0.3406	0.4121	0.1210	0.1930	0.1218	0.1333	0.1309	0.9069	0.1519	0.1185	0.1271
TS10	0.3564	0.5402	0.1239	0.1607	0.1248	0.1374	0.1334	0.8877	0.1333	0.1252	0.1333
TS11	0.4085	0.5336	0.1188	0.1594	0.1199	0.1392	0.1383	0.9475	0.1395	0.1297	0.1376
TS12	0.3791	0.6014	0.1412	0.1684	0.1451	0.1655	0.1665	0.9177	0.1673	0.1549	0.1649
TS13	0.3181	0.5000	0.1317	0.1706	0.1355	0.1564	0.1588	0.9221	0.1686	0.1454	0.1562
TS14	0.3551	0.5168	0.1121	0.1988	0.1119	0.1258	0.1161	0.9265	0.1490	0.1151	0.1132
TS15	0.4276	0.5804	0.1269	0.1428	0.1258	0.1395	0.1383	0.8914	0.1339	0.1338	0.1385
TS16	0.4087	0.5777	0.1354	0.1771	0.1321	0.1574	0.1558	0.8897	0.1490	0.1445	0.1556
TS17	0.4902	0.5529	0.1614	0.2193	0.1544	0.1600	0.1548	0.9022	0.1761	0.1522	0.1533
TS18	0.3965	0.4782	0.1398	0.1791	0.1391	0.1698	0.1674	0.9082	0.1719	0.1545	0.1658
TS19	0.4392	0.5995	0.1355	0.1552	0.1335	0.1570	0.1539	0.8804	0.1515	0.1460	0.1538
TS20	0.4086	0.4627	0.1939	0.2333	0.1888	0.1966	0.1901	0.8963	0.2039	0.1818	0.1892
TS21	0.4827	0.6328	0.1926	0.2281	0.1991	0.2062	0.2035	0.8162	0.2202	0.1980	0.2010
TS22	0.3273	0.4612	0.1280	0.1943	0.1249	0.1375	0.1313	0.9035	0.1589	0.1315	0.1295
TS23	0.3809	0.4469	0.1187	0.1702	0.1138	0.1229	0.1189	0.8567	0.1402	0.1196	0.1167
TS24	0.5249	0.6659	0.1540	0.1616	0.1516	0.1630	0.1624	0.8895	0.1531	0.1577	0.1629
TS25	0.4436	0.5933	0.1367	0.1677	0.1375	0.1654	0.1622	0.8609	0.1646	0.1474	0.1611
TS26	0.4269	0.6063	0.1664	0.2127	0.1582	0.1596	0.1561	0.8550	0.1681	0.1575	0.1559
TS27	0.4137	0.5378	0.1576	0.1881	0.1510	0.1618	0.1560	0.8544	0.1707	0.1492	0.1549
TS28	0.2554	0.2553	0.2252	0.2692	0.2272	0.2155	0.2150	0.2972	0.2226	0.2208	0.2156
TS29	0.2054	0.2045	0.1595	0.2174	0.1581	0.1488	0.1497	0.2422	0.1498	0.1557	0.1502
TS30	0.2336	0.2205	0.1866	0.2787	0.1903	0.1778	0.1753	0.2468	0.1748	0.1827	0.1760
TS31	0.2460	0.2505	0.2382	0.2822	0.2409	0.2278	0.2262	0.3078	0.2329	0.2327	0.2271
TS32	0.2611	0.2637	0.1906	0.2869	0.1872	0.1773	0.1707	0.2840	0.1812	0.1830	0.1726
TS33	0.2392	0.2382	0.1882	0.2599	0.1882	0.1852	0.1828	0.2336	0.1838	0.1871	0.1832
TS34	0.1929	0.1867	0.1511	0.2235	0.1481	0.1488	0.1463	0.1861	0.1461	0.1484	0.1466
TS35	1.0000	1.0000	1.0000	0.9269	1.0000	1.0000	1.0000	1.0000	4.0651	1.0000	1.0000
TS36	0.1876	0.1779	0.1342	0.2308	0.1342	0.1376	0.1347	0.1981	0.1354	0.1349	0.1349
TS37	0.2198	0.2173	0.1608	0.2440	0.1575	0.1573	0.1532	0.2123	0.1507	0.1572	0.1537
TS38	0.2540	0.2536	0.1703	0.2650	0.1698	0.1703	0.1650	0.2384	0.1614	0.1681	0.1654
TS39	0.2475	0.2617	0.2194	0.2657	0.2270	0.2169	0.2163	0.2996	0.2207	0.2170	0.2161
TS40	0.2917	0.2782	0.2776	0.3050	0.2764	0.2719	0.2726	0.3139	0.2692	0.2741	0.2726
TS41	0.2401	0.2315	0.1682	0.2897	0.1646	0.1666	0.1639	0.2384	0.1665	0.1655	0.1638
TS42	0.2552	0.2513	0.2092	0.2774	0.2038	0.2104	0.2078	0.2292	0.2027	0.2086	0.2079
TS43	0.1933	0.1893	0.1694	0.2476	0.1691	0.1712	0.1696	0.1564	0.1766	0.1701	0.1698
TS44	0.2317	0.2239	0.2174	0.2455	0.2178	0.2179	0.2176	0.2194	0.2213	0.2176	0.2175
TS45	0.4129	0.6143	0.2112	0.2701	0.2165	0.1677	0.1638	0.8325	0.1503	0.1915	0.1687
TS46	0.3517	0.5053	0.2306	0.2863	0.2500	0.2414	0.2397	0.8192	0.2194	0.2369	0.2413
TS47	0.3216	0.4171	0.2167	0.2663	0.2241	0.2156	0.2147	0.8525	0.2053	0.2150	0.2157
TS48	0.3965	0.6059	0.2667	0.3230	0.2723	0.2060	0.2127	0.7804	0.2045	0.2475	0.2183
TS49	0.3298	0.5534	0.2422	0.2774	0.2462	0.1822	0.1752	0.9000	0.1512	0.2195	0.1833
TS50	0.1966	0.1940	0.1974	0.2378	0.1983	0.1977	0.1979	0.1894	0.2106	0.1972	0.1977

**Table A50:** Temperature time series: Polya model (ρ=20%).

Time	DLM+BIC	DLM+EBIC	CDRec	GROUSE	ROSL	SoftImp	SVDImp	SVT	TeNMF	DynaMMo	TRMF
Series	$n_{u}$ = 180	$n_{u}$ = 60									
	s = 2	s = 2									
	$n_{3}$ = 180	$n_{3}$ = 60									
TS1	0.5009	0.3336	0.2148	0.2296	0.2221	0.2274	0.2254	0.2066	0.2266	0.2220	0.2253
TS2	0.4938	0.4107	0.1629	0.2285	0.1617	0.1900	0.1870	0.1576	0.1872	0.1842	0.1872
TS3	0.4288	0.3421	0.1707	0.1913	0.1694	0.1767	0.1775	0.2316	0.1766	0.1782	0.1773
TS4	0.5579	0.4574	0.2415	0.2603	0.2399	0.2250	0.2270	0.1722	0.2159	0.2320	0.2273
TS5	0.5366	0.4122	0.2320	0.2374	0.2339	0.2051	0.2058	0.1667	0.1940	0.2105	0.2059
TS6	0.4775	0.3953	0.1579	0.1903	0.1533	0.1771	0.1755	0.2069	0.1742	0.1756	0.1758
TS7	0.4476	0.4029	0.1160	0.1748	0.1269	0.1475	0.1427	0.2055	0.1532	0.1348	0.1422
TS8	0.2467	0.2492	0.2860	0.2822	0.2887	0.2675	0.2704	0.2028	0.2633	0.2714	0.2700
TS9	0.4822	0.4063	0.1144	0.1629	0.1248	0.1501	0.1439	0.1343	0.1470	0.1377	0.1441
TS10	0.4772	0.3742	0.1203	0.1846	0.1176	0.1484	0.1516	0.1520	0.1469	0.1551	0.1515
TS11	0.5225	0.4084	0.1054	0.1498	0.1101	0.1319	0.1274	0.1230	0.1356	0.1195	0.1270
TS12	0.5472	0.4172	0.1293	0.1875	0.1355	0.1738	0.1702	0.1709	0.1799	0.1591	0.1690
TS13	0.5398	0.4517	0.1080	0.1582	0.1064	0.1565	0.1480	0.1166	0.1547	0.1381	0.1480
TS14	0.5140	0.3466	0.1187	0.1447	0.1132	0.1531	0.1511	0.1456	0.1471	0.1537	0.1517
TS15	0.4963	0.4122	0.1474	0.1605	0.1393	0.1450	0.1455	0.1811	0.1418	0.1522	0.1461
TS16	0.3932	0.3368	0.1210	0.1437	0.1132	0.1456	0.1441	0.1488	0.1395	0.1484	0.1447
TS17	0.2360	0.2254	0.2388	0.2620	0.2421	0.2141	0.2173	0.1764	0.2087	0.2213	0.2171
TS18	0.4746	0.3250	0.1280	0.1712	0.1292	0.1671	0.1620	0.1312	0.1706	0.1511	0.1614
TS19	0.4566	0.3406	0.1440	0.1652	0.1387	0.1501	0.1506	0.1853	0.1504	0.1539	0.1507
TS20	0.5272	0.4319	0.1536	0.2015	0.1565	0.1853	0.1845	0.1811	0.1936	0.1746	0.1832
TS21	0.5419	0.4432	0.2130	0.2264	0.2053	0.2323	0.2313	0.2454	0.2274	0.2322	0.2318
TS22	0.3099	0.2875	0.1183	0.1495	0.1111	0.1431	0.1421	0.1437	0.1385	0.1452	0.1426
TS23	0.2725	0.2602	0.1393	0.1723	0.1452	0.1610	0.1523	0.1515	0.1605	0.1462	0.1527
TS24	0.4164	0.3403	0.1498	0.1738	0.1419	0.1674	0.1664	0.1953	0.1613	0.1713	0.1672
TS25	0.4002	0.3084	0.1228	0.1740	0.1257	0.1679	0.1651	0.1419	0.1737	0.1544	0.1641
TS26	0.4901	0.4492	0.1679	0.1722	0.1606	0.1707	0.1695	0.1521	0.1670	0.1754	0.1703
TS27	0.4729	0.4026	0.1638	0.1684	0.1569	0.1713	0.1660	0.1477	0.1648	0.1693	0.1669
TS28	0.2398	0.2282	0.2002	0.2347	0.1972	0.1837	0.1821	0.1512	0.2000	0.1845	0.1820
TS29	0.2641	0.2195	0.1712	0.2387	0.1758	0.1671	0.1636	0.1554	0.1665	0.1674	0.1641
TS30	0.2525	0.2515	0.1866	0.2906	0.1859	0.1787	0.1794	0.1918	0.1842	0.1804	0.1789
TS31	0.2681	0.2717	0.2562	0.3083	0.2556	0.2312	0.2277	0.1945	0.2562	0.2286	0.2273
TS32	0.2269	0.2203	0.1957	0.3144	0.1894	0.1804	0.1751	0.1922	0.1931	0.1777	0.1755
TS33	0.3122	0.2999	0.2276	0.3011	0.2297	0.2200	0.2182	0.2434	0.2209	0.2201	0.2181
TS34	0.3253	0.3288	0.1773	0.2371	0.1731	0.1768	0.1723	0.1539	0.1704	0.1719	0.1726
TS35	0.2186	0.2097	0.2428	0.3238	0.2514	0.2318	0.2289	0.2066	0.2447	0.2329	0.2292
TS36	0.2240	0.2038	0.1667	0.2424	0.1581	0.1604	0.1581	0.1508	0.1562	0.1592	0.1582
TS37	0.2306	0.2244	0.1574	0.2172	0.1493	0.1512	0.1477	0.1301	0.1468	0.1485	0.1479
TS38	0.2622	0.2514	0.1600	0.2326	0.1506	0.1590	0.1568	0.1423	0.1521	0.1572	0.1568
TS39	0.2885	0.2747	0.2216	0.2966	0.2253	0.2124	0.2115	0.1965	0.2220	0.2103	0.2109
TS40	0.2875	0.2890	0.2452	0.2812	0.2511	0.2420	0.2441	0.2234	0.2468	0.2425	0.2433
TS41	0.2280	0.2162	0.1660	0.2493	0.1652	0.1647	0.1651	0.1879	0.1635	0.1653	0.1648
TS42	0.2042	0.1922	0.1779	0.2339	0.1725	0.1774	0.1778	0.1754	0.1770	0.1772	0.1774
TS43	0.5196	0.4350	0.2014	0.2985	0.2322	0.1589	0.1390	0.2035	0.1674	0.1327	0.1382
TS44	0.2459	0.2355	0.2244	0.2541	0.2226	0.2238	0.2247	0.2003	0.2227	0.2237	0.2244
TS45	0.5464	0.3667	0.2240	0.2521	0.2297	0.1835	0.1914	0.1763	0.1913	0.2049	0.1920
TS46	0.4855	0.3586	0.2847	0.3087	0.2973	0.2273	0.2295	0.1972	0.2375	0.2449	0.2306
TS47	0.4797	0.4015	0.2107	0.2633	0.2337	0.1520	0.1475	0.2446	0.1782	0.1343	0.1449
TS48	0.2016	0.1743	0.1813	0.2179	0.1846	0.1841	0.1838	0.1462	0.1905	0.1833	0.1838
TS49	0.4279	0.3201	0.2499	0.2872	0.2682	0.1933	0.1866	0.2612	0.2278	0.1672	0.1835
TS50	0.1969	0.1836	0.1863	0.2320	0.1879	0.1853	0.1847	0.1396	0.1979	0.1846	0.1847

### Appendix A.5. Air Time Series: Numerical Results

#### Appendix A.5.1. Results obtained by using DLM

**Table A51:** Air time series: Sampling without replacement (ρ=5%).

Time	DLM+BIC	DLM+EBIC	CDRec	GROUSE	ROSL	SoftImp	SVDImp	SVT	TeNMF	DynaMMo	TRMF
Series	$n_{u}$ = 480	$n_{u}$ = 240									
	s = 3	s = 2									
	$n_{3}$ = 360	$n_{3}$ = 120									
TS1	0.2548	0.2434	0.3617	0.4714	0.3234	0.3569	0.3590	0.2071	0.3588	0.3554	0.3574
TS2	0.3420	0.3063	0.3064	0.5013	0.3400	0.3105	0.3126	0.0752	0.3036	0.3183	0.3108
TS3	0.2761	0.2445	0.3560	0.3704	0.4506	0.3646	0.3655	0.1843	0.3679	0.3399	0.3636
TS4	0.1893	0.2390	0.3125	1.0617	0.2286	0.3138	0.3153	0.2197	0.3143	0.2931	0.3136
TS5	0.2184	0.2281	0.3413	0.4287	0.4287	0.3391	0.3408	0.2255	0.3457	0.3185	0.3388
TS6	1.0038	0.9497	1.4207	0.9757	1.1487	1.2212	1.3292	0.9916	3.5458	1.1304	1.2493
TS7	0.9136	0.9201	3.9768	1.0591	1.8576	1.7752	2.1261	1.0322	2.0703	1.5106	1.8435
TS8	1.1018	1.0660	1.3826	1.5127	1.1369	1.6344	1.9063	1.0019	2.2160	1.4667	1.6949
TS9	0.2104	0.1867	0.2512	0.4374	0.3002	0.2473	0.2466	0.0605	0.2639	0.2456	0.2463
TS10	0.2302	0.2036	0.3029	0.3264	0.4377	0.3157	0.3090	0.1718	0.3123	0.3171	0.3119

**Table A52:** Air time series: Sampling without replacement (ρ=10%).

Time	DLM+BIC	DLM+EBIC	CDRec	GROUSE	ROSL	SoftImp	SVDImp	SVT	TeNMF	DynaMMo	TRMF
Series	$n_{u}$ = 360	$n_{u}$ = 240									
	s = 2	s = 2									
	$n_{3}$ = 240	$n_{3}$ = 120									
TS1	0.2260	0.2333	0.3531	0.4601	0.2724	0.3561	0.3544	0.1823	0.3637	0.3549	0.3548
TS2	0.2335	0.2322	0.2316	0.4501	0.2688	0.2351	0.2325	0.0997	0.2591	0.2399	0.2336
TS3	0.3041	0.3473	0.4930	0.4897	0.5394	0.4564	0.4605	0.1623	0.4518	0.4550	0.4576
TS4	0.2552	0.2373	0.3358	0.8513	0.4182	0.3435	0.3444	0.2040	0.3328	0.3214	0.3422
TS5	0.2240	0.2326	0.3017	0.5312	0.2902	0.2964	0.2974	0.2202	0.3070	0.2749	0.2961
TS6	0.9566	1.0421	1.8548	1.0113	0.9943	1.2095	1.5597	0.9890	3.0117	1.1014	1.2741
TS7	0.9877	0.9812	2.6434	0.9722	1.1576	1.2856	2.1441	0.9749	1.1235	1.0334	1.4263
TS8	1.0215	1.0439	1.3234	1.4777	1.0571	1.3343	1.4864	1.0025	1.4564	1.2735	1.3716
TS9	0.3168	0.2966	0.3150	0.7511	0.3492	0.2996	0.2967	0.0852	0.3049	0.2981	0.2984
TS10	0.3106	0.3138	0.4025	0.4248	0.5356	0.3931	0.3938	0.1614	0.3997	0.3956	0.3933

**Table A53:** Air time series: Sampling without replacement (ρ=15%).

Time	DLM+BIC	DLM+EBIC	CDRec	GROUSE	ROSL	SoftImp	SVDImp	SVT	TeNMF	DynaMMo	TRMF
Series	$n_{u}$ = 360	$n_{u}$ = 240									
	s = 2	s = 2									
	$n_{3}$ = 240	$n_{3}$ = 120									
TS1	0.2789	0.2906	0.3783	0.4658	0.3104	0.3636	0.3599	0.1866	0.3667	0.3662	0.3612
TS2	0.2654	0.2718	0.3353	0.5307	0.3985	0.3382	0.3417	0.1603	0.3347	0.3080	0.3377
TS3	0.2560	0.2453	0.3597	0.3793	0.3467	0.3696	0.3670	0.2484	0.3746	0.3576	0.3671
TS4	0.2605	0.2481	0.3754	1.3561	0.3773	0.3607	0.3636	0.2088	0.3749	0.3366	0.3599
TS5	0.2482	0.2572	0.3351	0.7791	0.3819	0.3330	0.3312	0.2633	0.3396	0.2984	0.3293
TS6	0.9320	0.9828	2.2586	1.0004	1.0343	1.5093	1.8791	0.9892	5.2137	1.3284	1.5798
TS7	0.9993	0.9758	1.6955	0.9870	1.6647	1.4416	1.6555	1.0016	1.8835	1.3103	1.4878
TS8	1.0081	0.9891	1.1758	1.2829	0.9740	1.2317	1.3925	0.9657	1.5170	1.1005	1.2599
TS9	0.2195	0.2244	0.3067	0.7159	0.3621	0.3075	0.3041	0.1375	0.3257	0.2871	0.3035
TS10	0.2888	0.2849	0.3855	0.4184	0.3424	0.3783	0.3787	0.2419	0.3801	0.3696	0.3776

**Table A54:** Air time series: Sampling without replacement (ρ=20%).

Time	DLM+BIC	DLM+EBIC	CDRec	GROUSE	ROSL	SoftImp	SVDImp	SVT	TeNMF	DynaMMo	TRMF
Series	$n_{u}$ = 240	$n_{u}$ = 240									
	s = 2	s = 2									
	$n_{3}$ = 120	$n_{3}$ = 120									
TS1	0.3360	0.3360	0.3692	0.4555	0.3136	0.3810	0.3755	0.3303	0.4384	0.3778	0.3728
TS2	0.2771	0.2771	0.3085	0.5713	0.3738	0.3314	0.3212	0.2395	0.4126	0.3092	0.3201
TS3	0.2942	0.2942	0.3581	0.3945	0.3290	0.3692	0.3695	0.2403	0.4176	0.3597	0.3632
TS4	0.2836	0.2836	0.4308	1.3230	0.4028	0.4374	0.4359	0.3515	0.5021	0.4230	0.4306
TS5	0.2735	0.2735	0.3024	0.5052	0.3423	0.3137	0.3156	0.3001	0.3826	0.2884	0.3078
TS6	0.8516	0.8516	2.2529	1.0877	1.0043	1.3900	2.5798	0.9602	5.4448	1.1512	1.5534
TS7	0.9478	0.9478	1.5730	1.0077	1.3867	1.3696	1.4932	1.0146	1.6426	1.3149	1.3537
TS8	0.9640	0.9640	1.0901	0.9955	0.9847	0.7871	1.0914	0.9654	1.0942	0.7891	0.8220
TS9	0.3211	0.3211	0.2708	0.3434	0.3477	0.2952	0.2850	0.2102	0.3677	0.2868	0.2842
TS10	0.2677	0.2677	0.3346	1.7345	0.3645	0.3433	0.3452	0.2293	0.4042	0.3192	0.3371

**Table A55:** Air time series: Polya model (ρ=5%).

Time	DLM+BIC	DLM+EBIC	CDRec	GROUSE	ROSL	SoftImp	SVDImp	SVT	TeNMF	DynaMMo	TRMF
Series	$n_{u}$ = 480	$n_{u}$ = 240									
	s = 3	s = 2									
	$n_{3}$ = 360	$n_{3}$ = 120									
TS1	0.3826	1.7971	0.3738	0.4482	0.2164	0.3600	0.3719	0.0724	0.3520	0.3407	0.3632
TS2	0.2326	0.2681	0.2223	0.2891	0.2141	0.2053	0.1942	0.1253	0.1511	0.1911	0.1987
TS3	0.6191	0.5637	0.2999	0.3188	0.3309	0.3248	0.3083	0.3882	0.3216	0.3491	0.3172
TS4	0.5318	1.3091	0.3255	0.5860	0.2383	0.3132	0.3216	0.1006	0.2968	0.3009	0.3160
TS5	0.2660	0.2456	0.2488	0.7728	0.2641	0.2549	0.2535	0.2387	0.2604	0.2767	0.2567
TS6	1.0299	1.0093	3.1321	0.9776	1.1752	1.5709	3.3088	0.9938	6.8670	1.2302	1.9523
TS7	0.9189	0.8498	2.8033	1.2668	0.8178	2.4239	2.7178	1.1546	2.7998	2.1727	2.5071
TS8	0.9988	1.2039	1.0655	1.0028	0.9345	1.0900	1.1379	0.9486	1.1983	1.0391	1.1117
TS9	0.2070	0.1876	0.2192	0.3855	0.2914	0.2271	0.2192	0.1303	0.2234	0.2151	0.2226
TS10	0.3934	0.4543	0.4303	0.4356	0.4397	0.4249	0.4234	0.3837	0.4210	0.4310	0.4245

**Table A56:** Air time series: Polya model (ρ=10%).

Time	DLM+BIC	DLM+EBIC	CDRec	GROUSE	ROSL	SoftImp	SVDImp	SVT	TeNMF	DynaMMo	TRMF
Series	$n_{u}$ = 360	$n_{u}$ = 240									
	s = 2	s = 2									
	$n_{3}$ = 240	$n_{3}$ = 120									
TS1	0.3828	0.3347	0.4136	0.5378	0.2347	0.4123	0.4206	0.2225	0.4389	0.3996	0.4155
TS2	0.3711	0.4010	0.1689	0.3873	0.1351	0.1440	0.1546	0.0903	0.2016	0.1566	0.1454
TS3	0.8215	0.8423	0.2970	0.4160	0.3940	0.3153	0.3076	0.2893	0.2986	0.3579	0.3122
TS4	0.3707	0.3925	0.3467	1.1146	0.3237	0.3533	0.3467	0.1581	0.3484	0.3533	0.3501
TS5	0.2089	0.2271	0.2782	0.3910	0.2739	0.2792	0.2819	0.2224	0.3100	0.2386	0.2776
TS6	1.0110	0.9953	1.3488	1.0194	0.9858	1.0008	1.0256	0.9915	2.4224	1.0059	1.0280
TS7	0.9765	1.1335	2.3091	1.0113	1.6921	1.9491	2.3484	1.0056	2.0894	1.4880	2.0728
TS8	1.0720	1.1029	1.0466	0.9774	0.9568	1.2491	1.3582	0.9838	1.5895	1.1268	1.2811
TS9	0.3136	0.3209	0.2539	0.4361	0.3218	0.2571	0.2537	0.0700	0.2642	0.2729	0.2551
TS10	0.3709	0.3264	0.3302	1.9792	0.4622	0.3464	0.3405	0.2966	0.3350	0.3438	0.3430

**Table A57:** Air time series: Polya model (ρ=15%).

Time	DLM+BIC	DLM+EBIC	CDRec	GROUSE	ROSL	SoftImp	SVDImp	SVT	TeNMF	DynaMMo	TRMF
Series	$n_{u}$ = 360	$n_{u}$ = 240									
	s = 2	s = 2									
	$n_{3}$ = 240	$n_{3}$ = 120									
TS1	0.5192	0.4733	0.3916	0.5431	0.5083	0.4324	0.4194	0.1858	0.4159	0.4469	0.4312
TS2	0.3979	0.3647	0.3572	0.7137	0.4051	0.3642	0.3495	0.1543	0.3559	0.3561	0.3632
TS3	0.3484	0.3657	0.4326	0.8600	0.4319	0.4295	0.4286	0.3250	0.4326	0.4046	0.4272
TS4	0.3155	0.3036	0.3451	0.7049	0.3009	0.3407	0.3624	0.1837	0.3478	0.3181	0.3473
TS5	0.4166	0.4184	0.2469	0.5565	0.2835	0.2448	0.2252	0.2353	0.2449	0.2305	0.2434
TS6	0.9531	0.9484	6.9985	0.8475	1.0014	2.8075	2.9721	0.9874	6.3240	2.2525	3.1954
TS7	0.9852	0.9813	0.7994	0.9676	0.8712	0.8760	1.1677	0.9842	0.8289	0.9183	0.8507
TS8	0.9797	1.0471	1.0436	0.9849	0.9746	1.6015	4.5712	0.9945	1.2359	1.2937	1.5801
TS9	0.5720	0.5421	0.2066	0.6070	0.2762	0.2091	0.2048	0.1289	0.2055	0.2239	0.2115
TS10	0.2719	0.3183	0.3879	0.5683	0.3452	0.3915	0.3932	0.2944	0.3943	0.3655	0.3891

**Table A58:** Air time series: Polya model (ρ=20%).

Time	DLM+BIC	DLM+EBIC	CDRec	GROUSE	ROSL	SoftImp	SVDImp	SVT	TeNMF	DynaMMo	TRMF
Series	$n_{u}$ = 240	$n_{u}$ = 240									
	s = 2	s = 2									
	$n_{3}$ = 120	$n_{3}$ = 120									
TS1	0.4380	0.4380	0.3378	0.4660	0.3678	0.3601	0.3433	0.2087	0.3450	0.3356	0.3592
TS2	0.4580	0.4580	0.2310	0.3691	0.2472	0.2181	0.2248	0.2287	0.2299	0.2450	0.2213
TS3	0.5015	0.5015	0.4977	0.4887	0.4900	0.4948	0.4957	0.3542	0.4848	0.4744	0.4946
TS4	0.5611	0.5611	0.4398	1.7964	0.4264	0.3678	0.3801	0.2205	0.3815	0.3907	0.3601
TS5	0.3849	0.3849	0.2709	1.1670	0.2478	0.2388	0.2321	0.2716	0.2663	0.2181	0.2554
TS6	1.0190	1.0190	1.9752	1.0082	1.2282	1.6292	2.7796	1.0096	4.4991	1.3856	1.7661
TS7	0.9810	0.9810	1.6607	1.0010	1.0619	0.9491	1.5645	0.9550	1.8831	0.9120	1.4091
TS8	1.0111	1.0111	2.8630	1.0511	0.9931	1.1262	1.8424	1.0292	1.8384	1.1041	1.3968
TS9	0.2704	0.2704	0.1817	0.6438	0.1860	0.1714	0.1748	0.2195	0.1746	0.1711	0.1705
TS10	0.5976	0.5976	0.4249	0.6685	0.4688	0.4117	0.4171	0.3380	0.4139	0.3972	0.4119

#### Appendix A.5.2. Results obtained by using DLM_1_

**Table A59:** Air time series: Sampling without replacement (ρ=5%).

Time	DLM${}_{1}$+BIC	DLM${}_{1}$+EBIC	CDRec	GROUSE	ROSL	SoftImp	SVDImp	SVT	TeNMF	DynaMMo	TRMF
Series	$n_{u}$ = 240	$n_{u}$ = 240									
	s = 2	s = 2									
	$n_{3}$ = 120	$n_{3}$ = 120									
TS1	0.1806	0.1806	0.2506	0.3348	0.1741	0.2430	0.2425	0.1068	0.2444	0.2526	0.2417
TS2	0.1401	0.1401	0.1925	0.2669	0.1780	0.1831	0.1936	0.0617	0.1823	0.1922	0.1874
TS3	0.1751	0.1751	0.2380	0.2586	0.2180	0.2797	0.2696	0.1200	0.2696	0.2613	0.2740
TS4	0.1360	0.1360	0.2194	0.7478	0.1094	0.2206	0.2201	0.1213	0.2150	0.2187	0.2185
TS5	0.1662	0.1662	0.2814	0.3220	0.3208	0.2855	0.2950	0.1998	0.2951	0.2735	0.2889
TS6	0.9998	0.9998	1.6842	1.0094	1.2006	1.2715	1.4354	0.9667	5.1728	1.1170	1.3166
TS7	0.8990	0.8990	3.5650	0.9825	1.7711	1.6373	1.9732	1.0133	1.6122	1.4027	1.7003
TS8	0.9698	0.9698	1.3527	1.5856	1.1573	1.5115	1.7568	1.0206	1.9446	1.3934	1.5456
TS9	0.1176	0.1176	0.1747	0.3268	0.1938	0.1705	0.1751	0.0497	0.1822	0.1721	0.1720
TS10	0.1425	0.1425	0.2172	0.2286	0.2603	0.2428	0.2318	0.1174	0.2324	0.2372	0.2371

**Table A60:** Air time series: Sampling without replacement (ρ=10%).

Time	DLM${}_{1}$+BIC	DLM${}_{1}$+EBIC	CDRec	GROUSE	ROSL	SoftImp	SVDImp	SVT	TeNMF	DynaMMo	TRMF
Series	$n_{u}$ = 240	$n_{u}$ = 240									
	s = 2	s = 2									
	$n_{3}$ = 120	$n_{3}$ = 120									
TS1	0.1725	0.1725	0.2567	0.3463	0.1418	0.2525	0.2489	0.1222	0.2540	0.2565	0.2499
TS2	0.1333	0.1333	0.1638	0.3036	0.1534	0.1627	0.1697	0.0803	0.1818	0.1689	0.1654
TS3	0.1730	0.1730	0.2889	0.3066	0.3030	0.3053	0.2970	0.1151	0.2858	0.2950	0.3005
TS4	0.1836	0.1836	0.2435	0.6181	0.2055	0.2412	0.2361	0.1225	0.2283	0.2331	0.2374
TS5	0.1429	0.1429	0.2389	0.3746	0.2215	0.2400	0.2451	0.1958	0.2407	0.2277	0.2419
TS6	1.0393	1.0393	2.6292	1.0942	1.0602	1.5342	2.0574	0.9959	4.6636	1.3077	1.6549
TS7	0.9381	0.9381	3.1959	0.9985	1.2745	1.5201	2.5258	0.9903	1.3818	1.1982	1.7120
TS8	0.9889	0.9889	1.2521	1.3667	1.0564	1.3437	1.4676	0.9851	1.5470	1.2737	1.3722
TS9	0.1533	0.1533	0.2212	0.5367	0.2141	0.2093	0.2163	0.0676	0.2236	0.2133	0.2129
TS10	0.1817	0.1817	0.2771	0.2842	0.3162	0.2853	0.2785	0.1177	0.2775	0.2875	0.2818

**Table A61:** Air time series: Sampling without replacement (ρ=15%).

Time	DLM${}_{1}$+BIC	DLM${}_{1}$+EBIC	CDRec	GROUSE	ROSL	SoftImp	SVDImp	SVT	TeNMF	DynaMMo	TRMF
Series	$n_{u}$ = 240	$n_{u}$ = 240									
	s = 2	s = 2									
	$n_{3}$ = 120	$n_{3}$ = 120									
TS1	0.2000	0.2000	0.2627	0.3320	0.1606	0.2590	0.2538	0.1380	0.2542	0.2554	0.2557
TS2	0.1489	0.1489	0.2135	0.3397	0.2355	0.2088	0.2176	0.1046	0.2117	0.1966	0.2112
TS3	0.1662	0.1662	0.2463	0.2770	0.2004	0.2776	0.2679	0.1765	0.2674	0.2657	0.2725
TS4	0.1921	0.1921	0.2562	0.8070	0.1819	0.2523	0.2541	0.1472	0.2597	0.2354	0.2508
TS5	0.1664	0.1664	0.2268	0.4770	0.2426	0.2255	0.2258	0.2159	0.2248	0.2166	0.2247
TS6	0.9974	0.9974	2.1018	0.9549	1.0288	1.4152	1.6952	0.9765	4.4863	1.2572	1.4719
TS7	0.9814	0.9814	1.7166	0.9840	1.6269	1.4536	1.7008	1.0007	1.7777	1.3089	1.5167
TS8	0.9656	0.9656	1.3730	1.4243	0.9909	1.4701	1.7077	0.9724	1.8521	1.2954	1.5243
TS9	0.1342	0.1342	0.2000	0.4481	0.2163	0.1985	0.2002	0.0859	0.2114	0.1920	0.1977
TS10	0.1579	0.1579	0.2587	0.2765	0.1876	0.2745	0.2652	0.1660	0.2655	0.2674	0.2700

**Table A62:** Air time series: Sampling without replacement (ρ=20%).

Time	DLM${}_{1}$+BIC	DLM${}_{1}$+EBIC	CDRec	GROUSE	ROSL	SoftImp	SVDImp	SVT	TeNMF	DynaMMo	TRMF
Series	$n_{u}$ = 240	$n_{u}$ = 240									
	s = 2	s = 2									
	$n_{3}$ = 120	$n_{3}$ = 120									
TS1	0.2064	0.2064	0.2542	0.3372	0.1598	0.2636	0.2546	0.1995	0.2726	0.2551	0.2556
TS2	0.1789	0.1789	0.2050	0.3712	0.2300	0.2146	0.2183	0.1416	0.2370	0.2120	0.2120
TS3	0.2094	0.2094	0.2382	0.2942	0.1803	0.2732	0.2656	0.1745	0.2675	0.2596	0.2669
TS4	0.1789	0.1789	0.3056	0.9343	0.1834	0.3134	0.3134	0.2102	0.3442	0.2980	0.3084
TS5	0.1848	0.1848	0.2261	0.3850	0.2411	0.2320	0.2381	0.2443	0.2447	0.2161	0.2304
TS6	0.9728	0.9728	2.8200	1.0155	1.0470	1.6987	3.1953	0.9899	7.1408	1.4269	1.9390
TS7	0.9866	0.9866	1.7151	1.0016	1.4446	1.3435	1.6010	1.0005	1.6119	1.2741	1.3968
TS8	0.9862	0.9862	1.3078	1.0912	1.0288	1.0638	1.3445	0.9785	1.3771	1.0509	1.0954
TS9	0.1782	0.1782	0.1681	0.2486	0.1991	0.1780	0.1809	0.1144	0.1976	0.1790	0.1750
TS10	0.1732	0.1732	0.2216	1.0829	0.1733	0.2513	0.2426	0.1561	0.2614	0.2373	0.2443

**Table A63:** Air time series: Polya model (ρ=5%).

Time	DLM${}_{1}$+BIC	DLM${}_{1}$+EBIC	CDRec	GROUSE	ROSL	SoftImp	SVDImp	SVT	TeNMF	DynaMMo	TRMF
Series	$n_{u}$ = 240	$n_{u}$ = 240									
	s = 2	s = 2									
	$n_{3}$ = 120	$n_{3}$ = 120									
TS1	0.5088	0.5088	0.3054	0.3556	0.1543	0.2877	0.3027	0.0661	0.2917	0.2854	0.2929
TS2	0.7519	0.7519	0.1213	0.1598	0.1052	0.1039	0.1157	0.0932	0.0844	0.0988	0.1083
TS3	0.8221	0.8221	0.1604	0.1934	0.1930	0.1994	0.1776	0.2517	0.1812	0.2253	0.1903
TS4	0.4411	0.4411	0.2315	0.3886	0.1253	0.2182	0.2320	0.0964	0.1996	0.2182	0.2256
TS5	0.6020	0.6020	0.1900	0.3807	0.1911	0.1986	0.2080	0.1889	0.1912	0.2289	0.2073
TS6	1.0071	1.0071	2.8668	1.0065	1.1411	1.4642	2.9797	0.9849	5.7104	1.1792	1.7468
TS7	0.9319	0.9319	1.9880	1.0857	1.0463	1.6361	1.7784	1.0626	1.7713	1.5185	1.6580
TS8	1.0061	1.0061	1.0786	1.0624	0.9665	1.1298	1.1688	0.9244	1.2290	1.0793	1.1483
TS9	0.4222	0.4222	0.1433	0.2760	0.1677	0.1490	0.1496	0.1075	0.1452	0.1466	0.1494
TS10	0.6303	0.6303	0.2991	0.3052	0.3037	0.3121	0.3018	0.2534	0.2981	0.3271	0.3069

**Table A64:** Air time series: Polya model (ρ=10%).

Time	DLM${}_{1}$+BIC	DLM${}_{1}$+EBIC	CDRec	GROUSE	ROSL	SoftImp	SVDImp	SVT	TeNMF	DynaMMo	TRMF
Series	$n_{u}$ = 240	$n_{u}$ = 240									
	s = 2	s = 2									
	$n_{3}$ = 120	$n_{3}$ = 120									
TS1	0.5283	0.5283	0.2968	0.4329	0.1385	0.3013	0.3033	0.1355	0.3175	0.2955	0.3009
TS2	0.7349	0.7349	0.1048	0.2555	0.0912	0.0912	0.1056	0.0637	0.1049	0.1082	0.0966
TS3	0.5905	0.5905	0.2080	0.3072	0.2064	0.2457	0.2316	0.1917	0.2152	0.2713	0.2399
TS4	0.7175	0.7175	0.2812	0.8631	0.2248	0.2910	0.2812	0.1289	0.2703	0.2865	0.2866
TS5	0.4916	0.4916	0.2003	0.2616	0.2009	0.2026	0.2075	0.1839	0.2072	0.1920	0.2033
TS6	1.0023	1.0023	1.7805	1.2420	0.9580	1.1554	1.1975	0.9724	5.6081	1.1132	1.1913
TS7	1.0023	1.0023	2.2820	1.0069	1.6319	1.9925	2.3358	0.9956	2.0378	1.5421	2.1045
TS8	0.9631	0.9631	1.1310	1.0313	0.9756	1.3546	1.5001	0.9863	1.8440	1.2005	1.4007
TS9	0.5298	0.5298	0.2051	0.3888	0.2201	0.2000	0.2042	0.0515	0.2086	0.2124	0.2010
TS10	0.6310	0.6310	0.2323	1.1563	0.2154	0.2711	0.2568	0.2026	0.2424	0.2698	0.2640

**Table A65:** Air time series: Polya model (ρ=15%).

Time	DLM${}_{1}$+BIC	DLM${}_{1}$+EBIC	CDRec	GROUSE	ROSL	SoftImp	SVDImp	SVT	TeNMF	DynaMMo	TRMF
Series	$n_{u}$ = 240	$n_{u}$ = 240									
	s = 2	s = 2									
	$n_{3}$ = 120	$n_{3}$ = 120									
TS1	0.5622	0.5622	0.2928	0.4109	0.2667	0.3137	0.3077	0.1306	0.3112	0.3186	0.3143
TS2	0.6802	0.6802	0.2502	0.4414	0.2663	0.2522	0.2493	0.1197	0.2535	0.2564	0.2544
TS3	0.5189	0.5189	0.2528	0.4743	0.2174	0.2858	0.2791	0.2225	0.2749	0.2780	0.2800
TS4	0.7111	0.7111	0.2216	0.4279	0.1437	0.2177	0.2312	0.1214	0.2165	0.2058	0.2180
TS5	0.8108	0.8108	0.1851	0.3939	0.1936	0.1838	0.1729	0.2067	0.1865	0.1796	0.1839
TS6	0.9986	0.9986	5.6154	0.9793	1.0723	2.1926	2.8821	0.9895	4.2939	1.7693	2.4950
TS7	1.0017	1.0017	1.2842	0.9989	1.2749	1.0758	1.2904	0.9889	1.2721	1.1044	1.0767
TS8	0.9836	0.9836	1.1275	1.0765	0.9813	1.7929	4.9608	0.9957	1.3869	1.4514	1.7940
TS9	0.8254	0.8254	0.1522	0.3364	0.1829	0.1508	0.1519	0.0931	0.1527	0.1590	0.1542
TS10	0.6731	0.6731	0.2600	0.3910	0.1760	0.2918	0.2849	0.2078	0.2849	0.2807	0.2873

**Table A66:** Air time series: Polya model (ρ=20%).

Time	DLM${}_{1}$+BIC	DLM${}_{1}$+EBIC	CDRec	GROUSE	ROSL	SoftImp	SVDImp	SVT	TeNMF	DynaMMo	TRMF
Series	$n_{u}$ = 240	$n_{u}$ = 240									
	s = 2	s = 2									
	$n_{3}$ = 120	$n_{3}$ = 120									
TS1	0.7628	0.7628	0.2291	0.3083	0.2214	0.2533	0.2285	0.1716	0.2234	0.2340	0.2431
TS2	0.6356	0.6356	0.1377	0.2089	0.1377	0.1296	0.1346	0.1462	0.1551	0.1462	0.1331
TS3	0.5838	0.5838	0.3079	0.3065	0.2938	0.3259	0.3104	0.2386	0.3176	0.3229	0.3204
TS4	0.7595	0.7595	0.2215	0.7733	0.2441	0.2074	0.2108	0.1542	0.2137	0.2245	0.2045
TS5	0.5418	0.5418	0.1548	0.4797	0.1359	0.1499	0.1586	0.2103	0.1698	0.1475	0.1534
TS6	0.9891	0.9891	2.1416	0.9992	1.2055	1.5187	2.8073	0.9850	6.8255	1.3007	1.6908
TS7	0.9971	0.9971	1.8998	1.0315	1.2626	1.1150	1.8402	0.9926	2.3124	1.0955	1.6477
TS8	1.0063	1.0063	3.1007	1.2966	1.0073	1.1635	1.9844	0.9885	2.1020	1.1104	1.5626
TS9	0.6672	0.6672	0.1067	0.4928	0.1051	0.1025	0.1094	0.1300	0.1119	0.1013	0.1061
TS10	0.6357	0.6357	0.2671	0.4178	0.2772	0.2694	0.2620	0.2291	0.2751	0.2771	0.2678
